# Phylogeny-Related Variations in Venomics: A Test in a Subset of Habu Snakes (*Protobothrops*)

**DOI:** 10.3390/toxins15050350

**Published:** 2023-05-21

**Authors:** Hong-Yan Zhao, Na He, Yan Sun, Yong-Chen Wang, Hao-Bing Zhang, Hui-Hui Chen, Ya-Qi Zhang, Jian-Fang Gao

**Affiliations:** Herpetological Research Center, College of Life and Environmental Sciences, Hangzhou Normal University, Hangzhou 311121, China

**Keywords:** *Protobothrops*, venom proteome, biochemical activity, phylogenetic signal, interspecific variation

## Abstract

We conducted a comparative analysis to unveil the divergence among venoms from a subset of Old World habu snakes (*Protobothrops*) in terms of venomic profiles and toxicological and enzymatic activities. A total of 14 protein families were identified in the venoms from these habu snakes, and 11 of them were shared among these venoms. The venoms of five adult habu snakes were overwhelmingly dominated by SVMP (32.56 ± 13.94%), PLA_2_ (22.93 ± 9.26%), and SVSP (16.27 ± 4.79%), with a total abundance of over 65%, while the subadult *P. mangshanensis* had an extremely low abundance of PLA_2_ (1.23%) but a high abundance of CTL (51.47%), followed by SVMP (22.06%) and SVSP (10.90%). Apparent interspecific variations in lethality and enzymatic activities were also explored in habu snake venoms, but no variations in myotoxicity were found. Except for SVSP, the resemblance of the relatives within *Protobothrops* in other venom traits was estimated to deviate from Brownian motion evolution based on phylogenetic signals. A comparative analysis further validated that the degree of covariation between phylogeny and venom variation is evolutionarily labile and varies among clades of closely related snakes. Our findings indicate a high level of interspecific variation in the venom proteomes of habu snakes, both in the presence or absence and the relative abundance of venom protein families, and that these venoms might have evolved under a combination of adaptive and neutral mechanisms.

## 1. Introduction

Snake venom has been widely validated to be composed of enzymes and proteins without enzymatic activity that belong to a few protein families and are regarded as an innovative biochemical weapon to assist different venomous snakes in capturing and digesting different prey [1,2]. The general concept is that the evolution of snake venom was driven by natural selection to adapt to the diversity of diets during the radiation of snakes [3,4]. It has further been claimed that properties of snake venom are associated with the resource spectrum in the habitat of the snakes, and that a highly productive environment or phylogenetically diverse diets could have promoted the development of venom with more complexity and potency [5,6,7]. However, an alternative “overkill” concept proposes that the evolution of snake venom might be subject to relaxed selection because of its high toxicity and a far higher injected dose than minimally necessary for capturing prey [8,9], by virtue of which the complexity of snake venom may be closely related to the phylogeny of the snakes [10]. This prediction is supported by empirical observations [11]; however, two other studies on phylogeny-related venom variation found that none of the major protein families were associated with phylogeny, and one family (CRISP) was even significantly associated with diet [10,12]. However, it was still declared that venom divergence can track the phylogeny of the genus *Agkistrodon* to a greater extent than that of *Sistrurus*, based on a comparison of phylogenetic signal strength [12]. The conclusion from a recent investigation of two habu snake species (*Protobothrops jerdonii* and *P. mucrosquamatus*) at a microevolutionary level strikingly integrated these two concepts mentioned above, i.e., that snake venom evolved under a combination of adaptive and neutral mechanisms [13]. Thus, it would be worthwhile examining whether this conclusion is applicable to the variations in venom composition at the macroevolutionary level in habu snakes (genus *Protobothrops*).

As Old-World pit vipers, habu snakes have been highly concerned in their phylogenetic relationships, biogeological patterns, and evolutionary histories. These species mainly occur in South Asia, Southeast Asia, and East Asia, and have been inferred to originate from ~20.64 mya and diverged within the genus from ~17.20 mya, with experience of 38 dispersal and 11 vicariant events [14]. They were initially considered to comprise eight species, including *P. cornutus*, *P. elegans*, *P. flavoviridis*, *P. jerdonii*, *P. kaulbacki*, *P. mucrosquamatus*, *P. tokarensis*, and *P. xiangchengensis* [15]. Due to seven more members (*P. dabieshanensis*, *P. himalayanus*, *P. kelomohy*, *P. mangshanensis*, *P. maolanensis*, *P. sieversorum*, and *P. trungkhanensis*) having been designated or revised from synonymous species over the last two decades, habu snakes were recently recognized to comprise 15 validated species [16,17,18,19,20]. Phylogenetic analysis based on multilocus gene markers robustly classified 14 habu snakes into four clades, in which the first clade comprises *P. himalayanus*, *P. kaulbacki*, and *P. sieversorum* and the second clade only comprises *P. mangshanensis*, while the other two clades contain the remaining four and six *Protobothrops* species, respectively [21]. The first clade should also contain a very recently described species (*P. kelomohy*) [20]. Of note, the phylogenetic relationships of the habu snakes in some clades are still very complicated and should be further clarified [21].

On the other hand, a complicated natural history and taxonomical relationships might indicate a diversity in venom profiles and snakebite envenomation mechanisms, which have also resulted in habu snakes receiving varying degrees of attention. Among these, *P. mucrosquamatus* might potentially cause the heaviest snakebite burden, as this species occupies the largest distribution area and threatens the greatest proportion of the human population (8.04% of the world’s population), and thus is well-known as the highest medically important habu snake [22,23]. *P. flavoviridis* is another highest medically important habu snake, for which epidemiological and clinical data have been exactly tracked, although this species ranges over a relatively small distribution area and threatens a very small proportion of the human population (<0.01% of the world’s population) [23,24]. Victims envenomed by *P. mucrosquamatus* and *P. flavoviridis* always suffer similar symptoms, such as local swelling, necrosis, bleeding at the bite site, vomiting, loss of consciousness, hypotension, and even acute renal failure, in addition to other species-specific symptoms (e.g., severe pain and burning sensation, festering, dizziness, and blurred vision from *P. mucrosquamatus*; cyanosis and compartment syndrome from *P. flavoviridis*) [25,26]. Epidemiological data indicate that the regional snakebite frequency of *P. mucrosquamatus* ranges from 1.40% (Nanchang, Jiangxi; 2017–2019) to 95.04% (Chongqin; 2019–2020) in China [27,28], and the snakebite incidence has been estimated to range from 0.16 to 1.16/100,000 population per year, whereas the snakebite incidence caused by *P. flavoviridis* was estimated to be 35/1,000,000 population per year during 1977–1984 in Japan [29]. To better understand and treat the snakebites from these two species, their venom compositions have been elucidated and are mainly comprised of snake venom metalloproteinase (SVMP), snake venom serine proteinase (SVSP), phospholipase A_2_ (PLA_2_), cysteine-rich secretory protein (CRISP), bradykinin-potentiating and C-type natriuretic peptides (BPP/CNP), and C-type lectin (CTL) [30,31], and the relative abundance of the same protein family can also shift due to the different sources of venom and -omics technology [32,33,34,35]. However, most habu snakes are mainly endemic to small areas or lack enough detailed epidemiological and clinical data, and are classified as secondary medically important venomous snakes [23]. Except for *P. elegans*, *P. kelomohy*, and *P. mangshanensis* [33,36,37], the global venom composition of the remaining habu snakes has been less studied. Thus, the mystery of the venom profiles across the genus *Protobothrops* still needs to be comprehensively solved.

**Figure 1:**
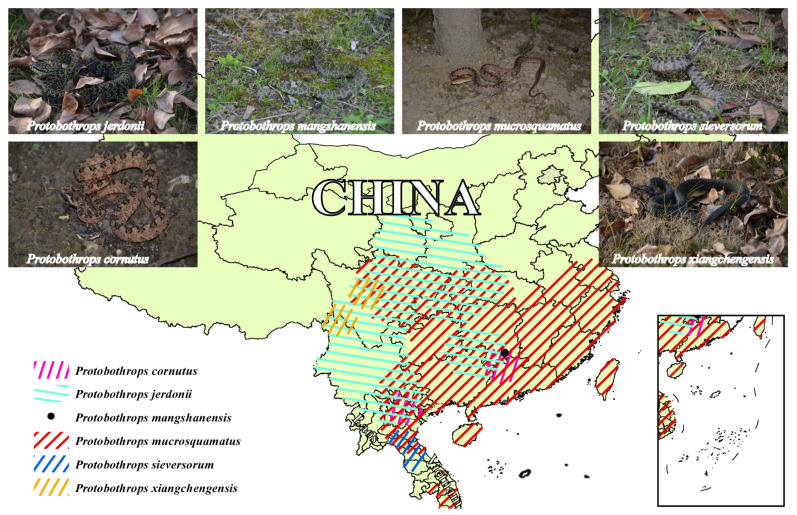
Major geographical distribution regions of the six habu snakes in the current study. The distribution areas of these snakes were illustrated by ArcGIS based on the data from the GBIF website and complied with the detailed descriptions in “Snakes of China” edited by Ermi Zhao [40]. The animal images were photographed by Jian-Fang Gao; the *P. mangshanensis* was a subadult specimen and the other habu snakes were adult specimens. A high-resolution image can be found in Figure S1.

Here, we employed a combined proteomic strategy [38,39] to conduct a relative quantitative investigation of venom profiles from six habu snakes (*P. cornutus*, *P. jerdonii*, *P. mangshanensis*, *P. mucrosquamatus*, *P. sieversorum*, and *P. xiangchengensis*; Figure 1), and used mice and specific substrates to determine the toxicological and enzymatic activities of these venoms. We also integrated the venom profiles from four other habu snakes that were previously characterized to calculate the phylogenetic signal strength of major venom components, and venom complexity and potency across the genus *Protobothrops*. Our results will enrich the background data for systematically exploring the interspecific variations in venom composition and function of the habu snakes, and preliminarily reveal whether these variations are related to phylogeny.

## 2. Results and Discussion

### 2.1. Chromatographic and Electrophoretic Profiles of Venoms from Six Habu Snakes

**Figure 2:**
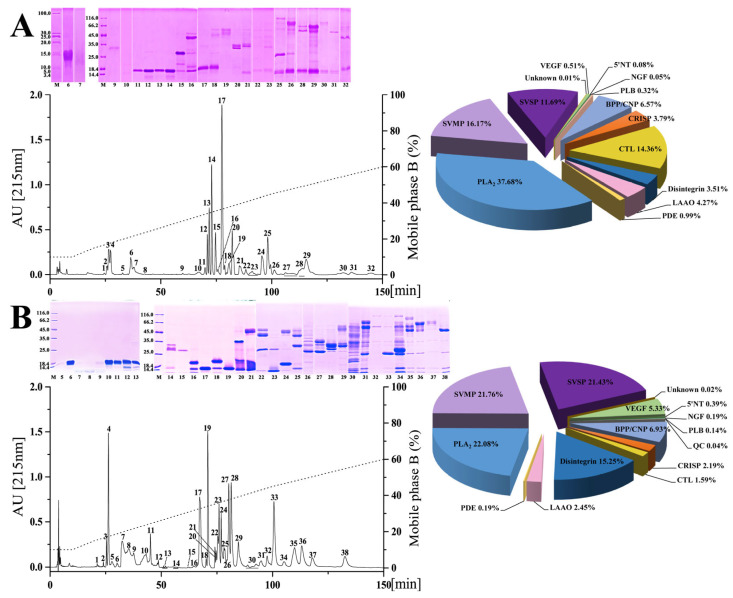

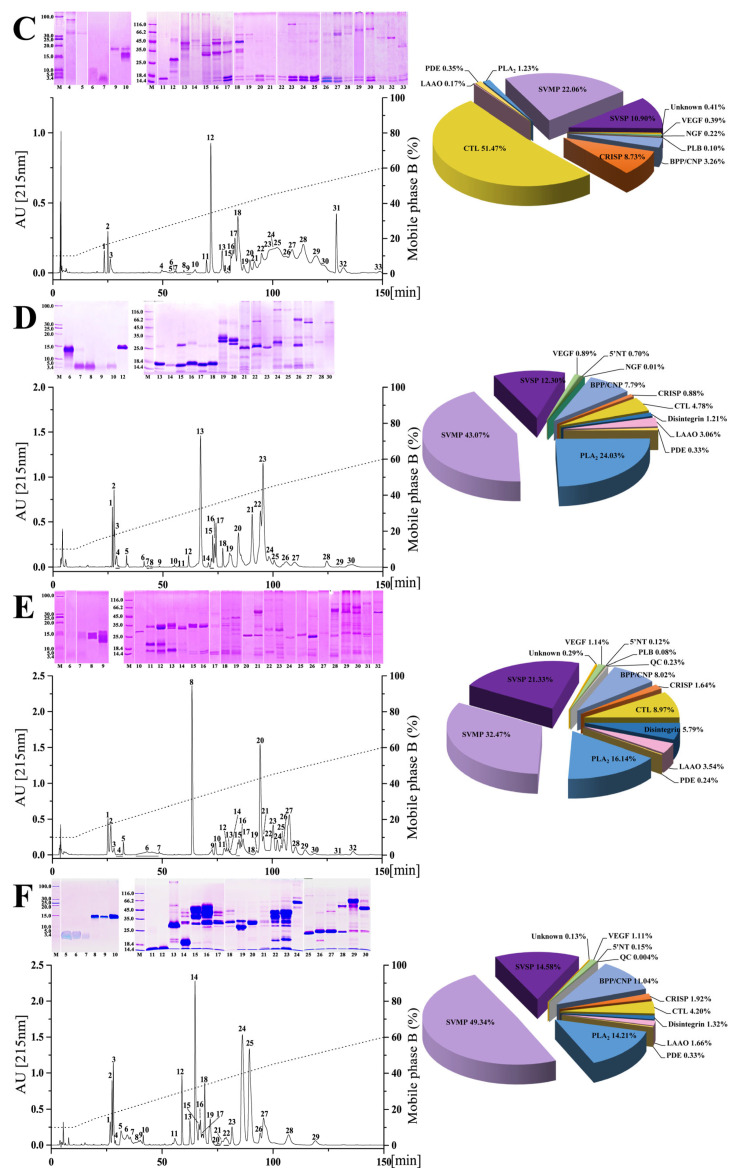
The venom proteomic profiles of six habu snakes. The venom proteins of *P. cornutus* (**A**), *P. jerdonii* (**B**), *P. mangshanensis* (**C**), *P. mucrosquamatus* (**D**), *P. sieversorum* (**E**), and *P. xiangchengensis* (**F**) were fractionated on a C18 column as described in the Materials and Methods. The RP-HPLC eluted fractions were collected and separated by SDS-PAGE under reducing conditions (on the left of panels). Protein bands from gels were digested with trypsin and identified by MALDI-TOF/TOF-MS and nESI-MS/MS. The details of the sequenced peptides and assigned protein families are listed in Supplementary Tables S1–S6. The pie charts (on the right of panels) display the relative abundance of the toxin families in the relevant venoms of habu snakes. The venom of *P. mangshanensis* was collected from subadult specimens, and the venom of the other habu snakes were collected from adult specimens. SVMP, snake venom metalloproteinase; SVSP, snake venom serine proteinase; PLA_2_, phospholipase A_2_; CRISP, cysteine-rich secretory protein; BPP/CNP, bradykinin-potentiating and C-type natriuretic peptides; CTL, C-type lectin; LAAO, L-amino acid oxidase; VEGF, vascular endothelial growth factor; NGF, nerve growth factor; PDE, phosphodiesterase; 5′NT, 5′-nucleotidase; PLB, phospholipase B; QC, glutaminyl-peptide cyclotransferase. A high-resolution image can be found in Figure S2.

Conventionally, the apparent properties of venom proteins can be simply distinguished by chromatography and electrophoresis [41,42,43]. In the current study, interspecific variations could be clearly observed in the chromatographic and the electrophoretic profiles of the venoms from six habu snakes (Figure 2). Specifically, a total amount of 29–38 chromatographic fractions could be resolved from these venoms, among which the *P. xiangchengensis* and *P. jerdonii* venoms expressed the least and the most fractions, respectively. The retention time of most fractions ranged from 25 to 125 min using the current separation procedure, and apparently varied among all the venoms. The components of the first 3–5 chromatographic fractions from these venoms could not be visualized in the electrophoretogram, while the remaining fractions could be further separated by electrophoresis and stained with CBB-250. The fractions expressing simple protein bands were mainly located at the retention times before 72 min in *P. cornutus*, *P. jerdonii*, *P. mangshanensis*, and *P. murcosquamatus* venoms, while those were located at the retention times before 60 and 75 min in *P. xiangchengensis* and *P. sieversorum* venoms, respectively. Moreover, the fractions with long retention times expressed more protein bands in the electrophoretogram in all venoms, among which *P. jerdonii* and *P. sieversorum* venoms were resolved with relatively more such fractions.

### 2.2. Proteomic Profiles of Venoms from Six Habu Snakes

Snake venom can potentially serve as a resource library with phylogeny clues, and exploration of its detailed composition by mass spectrometry technology has been highly concerned and advanced over the past ten years [38,44]. Here, based on the chromatographic and electrophoretic profiles, 4–8 chromatographic fractions from each venom were found to express a low abundance of proteins or contain components that cannot be displayed by electrophoresis, and thus were conducted by in-solution tryptic digestion and identified by nESI-MS/MS analysis. The remaining chromatographic fractions with visible protein bands were subjected to in-gel tryptic digestion, and then the peptides were identified by MALDI-TOF-MS/MS or nESI-MS/MS analysis. In general, the lowest and the highest number of protein bands for mass spectrometry analysis were 89 (*P. cornutus* venom) and 181 (*P. jerdonii* venom), respectively; more than 150 protein bands were identified in both *P. jerdonii* and *P. sieversorum* venoms (Figure 2 and Supplementary Tables S1–S6). Similar to the findings where the venom components could be classified into a few protein families in most venomous snakes [45], the mass spectrometry analysis identified 14 protein families in total in the fractions and protein bands of the venoms from six habu snakes, including SVMP (snake venom metalloproteinase), SVSP (snake venom serine protease), PLA_2_ (phospholipase A_2_), CRISP (cysteine-rich secretory protein), disintegrin, BPP/CNP (bradykinin-potentiating peptide/C-type natriuretic peptides), CTL (C-type lectin), LAAO (L-amino acid oxidase), NGF (nerve growth factor), VEGF (vascular endothelial growth factor), PDE (phosphodiesterase), PLB (phospholipase B), 5′NT (5′-nucleotidase), and QC (glutamine peptide cyclotransferase).

Remarkable variations were observed between the venom proteomes of five adult habu snakes in the current study, both in the presence or absence and the relative abundance of venom protein families (pie charts in Figure 2 and Table 1). Specifically, 11 protein families were shared among these habu snake venoms, with SVMP (32.56 ± 13.94% of the total venom proteome), PLA_2_ (22.93 ± 9.26%), and SVSP (16.27 ± 4.79%) as the three predominate protein families, which were expressed with a total abundance of higher than 65% in all five snake venoms with that in *P. mucrosquamatus* venom even up to 79.88%. The other common protein families between these habu snake venoms with a relatively low abundance included CRISP which accounted for 0.88–3.79% of the total venom proteome, disintegrin (1.21–15.25%), BPP/CNP (6.57–11.04%), CTL (1.59–14.26%), LAAO (1.66–4.27%), VEGF (0.51–5.33%), PDE (0.19–0.99%), and 5′NT (0.08–0.7%). The remaining three protein families including NGF, PLB, and QC with an average relative abundance of less than 0.15% were inconsistently present in these venoms, and they were only found in no more than three habu snake venoms. Moreover, four of these protein families (5′NT, NGF, PLB, and QC) can be considered trace components due to their very low average relative abundance (<0.3%) in these five snake venoms. These adult habu snakes were estimated to share relatively low similarities between their venom proteomes with an average protein similarity coefficient (PSC) of 17.7% (ranging from 7.6% to 39.2%, Table 2), which was similar to those estimated within the genera *Atropoides* (14–16%) [43] and *Bitis* (7–28%) [1], but apparently lower than those within the genus *Bothrops* (65–70%) [46]. Thus, these results might indicate a high level of interspecific variation in venom proteomes within the genus *Protobothrops*. Moreover, the criteria applied to determine the same or highly similar toxin proteins in the current study were not as strict as those in previous studies [1,43], and thus the real PSCs of these habu snake venoms may be lower than those in Table 2.

These results are consistent with the previous investigations on the venom proteomes of three other habu snakes, which were also overwhelmingly dominated by SVMP, PLA_2_, and SVSP (*P. elegans* 71.4%, *P. flavoviridis* 87.92%, and *P. kelomohy* 86.27% of the total abundance; Table 1) [31,33,36]; *P. flavoviridis* undoubtedly expressed the highest abundance of PLA_2_ (55.14%) in its venom proteome among all adult habu snakes. However, the relative abundances of these three protein families were highly variable among these venoms, especially the abundance of SVSP in *P. kelomohy* which was 20.8-fold that of *P. flavoviridis*. Apparent uniformity in the presence or absence, as well as the relative abundance, of the remaining 11 protein families, could also be observed in these habu snake venoms. As for the venom proteome of *P. mucrosquamatus*, a moderate level of geographical variation in the relative abundance of each protein family could be found between the samples from mainland China (current study) and China’s Taiwan (venom proteome decomplexed by Villalta and her colleagues, [30]) (Table 1); however, a great divergence in the presence or absence and relative abundance of components could be observed between the venom samples from these two populations, one of which from China’s Taiwan was elucidated by Liu and his colleagues based on genomic data [32]. Such differences could be due to the different sensitivities of the strategies for proteomic identification, as well as the differences in methodologies for relative abundance quantitation and the database for peptide assignment [47].

As an endangered species with an eye-catching appearance, *P. mangshanensis* was still mysterious due to the rare incidence of snakebites, and once received attention for some key venom components and their functions as well as a two-dimensional electrophoresis profile of its venom proteins, although its global venom proteome has never been decomplexed [48,49,50,51]. PLA_2_ was one of these studied components and accounted for 58% of the total abundance of adult venom proteins, and is also considered the major component inducing the edema, local inflammation, and muscle necrosis in victims envenomed by *P. mangshanensis* [50]. Of note, the subadult *P. mangshanensis* venom in the current study had an extremely low abundance of PLA_2_ (1.23%) (pie charts in Figure 2 and Table 1); the adult venom contained 47.2-fold more PLA_2_than the subadult venom of *P. mangshanensis*, which might be attributed to an ontogenetic shift in snake venom composition. In the global proteome profile of subadult *P. mangshanensis* venom, CTL was the most abundant component (51.47% of the total venom proteome), followed by SVMP (22.06%) and SVSP (10.90%). Unfortunately, the growth status and whereabouts of these two subadult specimens could not be continuously tracked after contacting the anonymous reptile enthusiast again, and thus we could not conduct global proteome analyses on the adult venom.

Statistical analyses further indicated the degrees of variation in the relative abundance of the 14 protein families in the venoms of eight adult habu snakes (three in previous studies [31,33,36] and five in the current study) ranged from 32.5 to 233.0 (coefficients of variations, CV), and the average CV of three highly abundant protein families (47.8 for SVMP, SVSP, and PLA_2_) was much lower than that of trace protein families (206.6 for NGF, PLB, 5′NT, and QC). Similar trends were found in *Agkistrodon* snakes with an average CV of 19.5 estimated for highly abundant protein families (SVMP, SVSP, and PLA_2_) and 71.3 for low abundance families (CRISP, disintegrin, CTL, LAAO, and NGF) [12], as well as in *Sistrurus* snakes with CV values of 23.9 and 125.4, respectively [10]. These results could be because the components with a high abundance rather than those with a low (trace) abundance are generally considered the major contributors to the function of the snake venom, and thus it is more important to maintain the stability of their relative abundance than that of the latter [52].

**Table 1:** Overview of the relative abundance of protein families in venoms from habu snakes.

Species	% of Total Venom Proteins									
	*P. cornutus*	*P. jerdonii*	*P. mangshanensis*	*P. mucrosquamatus*		*P. sieversorum*	*P. xiangchengensis*	*P. elegans*	*P. flavoviridis*	*P. kelomohy*
Location	Mainland China	Mainland China	Mainland China	Mainland China	China’s Taiwan	Vietnam	Mainland China	Japan	Japan	Thailand
Reference	Current study	Current study	Current study	Current study	Villalta et al. [30]	Current study	Current study	Aird et al. [33], Yamakawa et al. [53]	Huang et al. [54], Damm et al. [31]	Chanhome et al. [36]
LD_50_ (µg/g)	0.61 (0.46–0.81)	1.91 (1.73–2.11)	1.23 (0.98–1.54)	3.82 (3.50–4.17)	2.17	1.23 (1.07–1.41)	1.79 (1.51–2.11)	4.85	2.60	0.67 (0.58–0.78)
Separation	RP-HPLC SDS-PAGE	RP-HPLC SDS-PAGE	RP-HPLC SDS-PAGE	RP-HPLC SDS-PAGE	RP-HPLC SDS-PAGE	RP-HPLC SDS-PAGE	RP-HPLC SDS-PAGE	-	RP-HPLC SDS-PAGE	SDS-PAGE
Digestion	In-gel	In-gel	In-gel	In-gel	In-gel	In-gel	In-gel	In-solution	In-gel	In-gel
MS	LC-MS/MS	MALDI-TOF LC-MS/MS	LC-MS/MS	LC-MS/MS	MALDI-TOF LC-MS/MS	LC-MS/MS	MALDI-TOF LC-MS/MS	LC-MS/MS	LC-MS/MS	LC-MS/MS
SVMP	16.17	21.76	22.06	43.07	43.40	32.47	49.34	39.40	31.34	40.85
SVSP	11.69	21.43	10.90	12.30	10.40	21.33	14.58	9.50	1.44	29.93
PLA_2_	37.68	22.10	1.23	24.51	23.50	16.14	14.21	22.50	55.14	15.49
CRISP	3.79	2.19	8.73	0.88	0.80	1.64	1.92	1.80	1.83	1.41
Disintegrin	3.51	15.25	-	1.21	0.80	5.79	1.32	-	-	0.35
BPP/CNP	6.57	6.93	3.26	7.79	3.60	8.02	11.04	0.10	-	-
CTL	14.36	1.59	51.47	4.78	3.90	8.97	4.20	2.90	2.78	-
LAAO	4.27	2.45	0.17	3.06	2.00	3.54	1.66	9.80	0.71	3.87
VEGF	0.51	5.33	0.39	0.89	-	1.14	1.11	1.80	-	0.70
NGF	0.05	0.19	0.22	0.01	-	-	-	0.50	-	1.76
PDE	0.99	0.19	1.06	0.80	-	0.24	0.33	4.10	0.07	-
5′NT	0.08	0.39	-	0.70	-	0.12	0.15	2.80	0.02	-
PLB	0.32	0.14	0.10	-	-	0.08	-	2.20	-	-
QC	-	0.04	-	-	-	0.23	0.004	1.40	-	-
Others	-	-	-	-	3.30	-	-	1.00	6.39	5.63
Unknown	0.01	0.02	0.41	-	-	0.29	0.13	0.30	0.27	-

The venom of *P. mangshanensis* was collected from subadult specimens. SVMP, snake venom metalloproteinase; SVSP, snake venom serine proteinase; PLA_2_, phospholipase A_2_; CRISP, cysteine-rich secretory protein; BPP/CNP, bradykinin-potentiating and C-type natriuretic peptides; CTL, C-type lectin; LAAO, L-amino acid oxidase; VEGF, vascular endothelial growth factor; NGF, nerve growth factor; PDE, phosphodiesterase; 5′NT, 5′-nucleotidase; PLB, phospholipase B; QC, glutaminyl-peptide cyclotransferase. LD_50_: dose that induces death in 50% of mice, confidence limits (95%) are listed in parentheses.

**Table 2:** Protein similarity coefficients (%) of the venoms from five adult habu snakes.

	*P. cornutus*	*P. jerdonii*	*P. mucrosquamatus*	*P. sieversorum*	*P. xiangchengensis*
*P. cornutus*	-	14.4	15.8	18.9	9.6
*P. jerdonii*	-	-	15.2	14.3	23.7
*P. mucrosquamatus*	-	-	-	39.2	18.0
*P. sieversorum*	-	-	-	-	7.6
*P. xiangchengensis*	-	-	-	-	-

### 2.3. Toxicological and Enzymatic Activities of Venoms from Six Habu Snakes

As a complex arsenal, snake venom has complicated biochemical functions, which can facilitate the prediction of the clinical manifestations of envenomations, as well as the diagnosis and treatment of snakebites [55]. Given the distinct proteomic profiles of habu snake venoms that were unraveled in the current study, we further determined their toxicological and enzymatic activities to explore the potential variations in biochemical functions among these venoms. The venoms from adult habu snakes in the current study showed relatively high variability in their toxicity to ICR mice, among which the *P. cornutus* and *P. mucrosquamatus* venoms, respectively, produced the strongest (LD_50_ = 0.61 μg/g) and the weakest (LD_50_ = 3.82 μg/g) toxicities among these venoms, and the former was 6-fold more toxic than the latter (Table 1). These venoms all seemed more toxic than *P. elegans* venom (LD_50_ = 4.85 μg/g, [53]) and only the *P. cornutus* venom showed a strong toxicity similar to that of *P. kelomohy* venom (LD_50_ = 0.67 μg/g, [36]). The other habu snake venoms investigated in a previous study [31] and the current study had moderate levels of toxicity. Moreover, ontogenetic shift (LD_50_ of subadult and adult *P. mangshanensis* venom was 1.23 and 4.0 μg/g, respectively [48]; the difference in toxicity could be attributed to the difference in CTL relative abundance between these two venoms) and geographical variation (LD_50_ of mainland China and China’s Taiwan *P. mucrosquamatus* venom was 3.82 and 2.17 μg/g, respectively [30]) in the toxicity of venoms could also be observed in the habu snakes, which should be considered in the clinical diagnosis and treatment of snakebites caused by them. In regard to the toxicity of venoms from the less clinically important habu snakes, such as *P. cornutus*, *P. kelomohy*, *P. sieversorum*, and *P. xiangchengensis*, the toxicity was apparently strong among all venoms; therefore, the envenomations caused by them and their clinical treatment should be given more attention.

**Figure 3:**
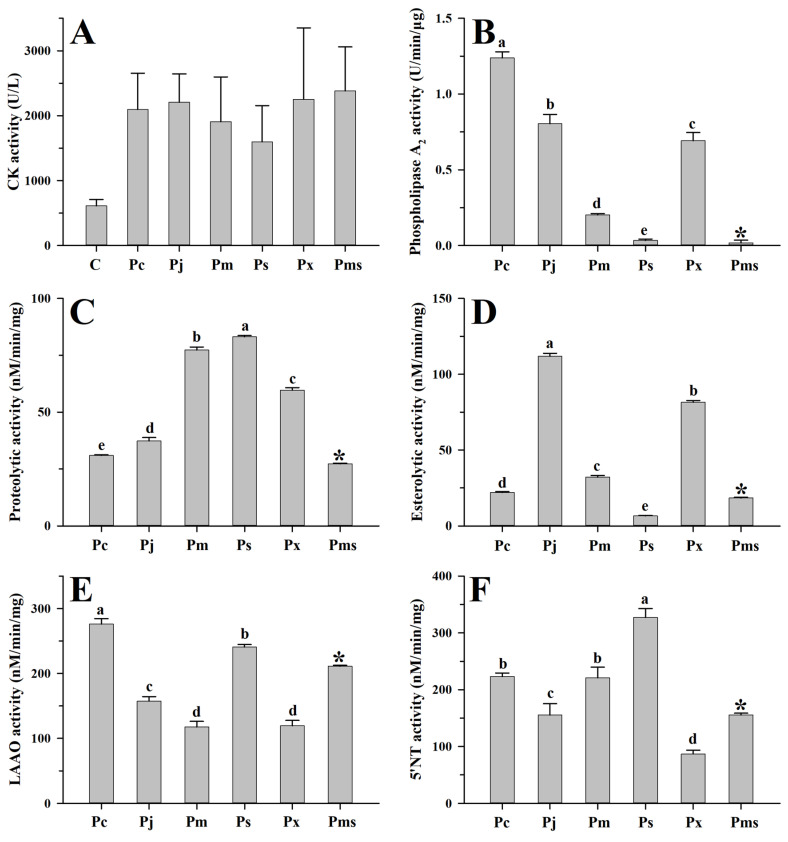
Myotoxicity and enzymatic activities of venoms from habu snakes. C, saline; Pc, *P. cornutus*; Pj, *P. jerdonii*; Pm, *P. mucrosquamatus*; Ps, *P. sieversorum*; Px, *P. xiangchengensis*; Pms, *P. mangshanensis*. Data are expressed as mean value ± SD ((**A**): *n* = 4; (**B**–**F**): *n* = 3). The enzymatic activities of *P. mangshanensis* venom (indicated with “*” above the column) were excluded from the statistical analysis due to the venom having been collected from subadult specimens. The significance level is set at α = 0.05, a > b > c > d > e. A high-resolution image can be found in Figure S3.

**Figure 4:**
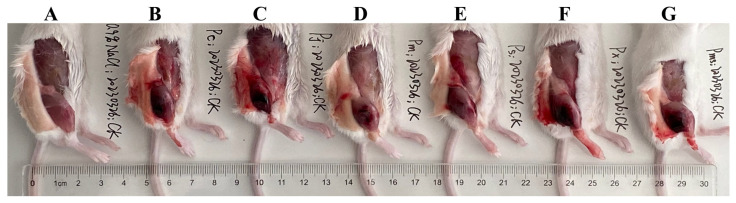
Local symptoms in the gastrocnemius muscle of the mice after injection of (**A**) saline; (**B**) *P. cornutus*; (**C**) *P. jerdonii*; (**D**) *P. mucrosquamatus*; (**E**) *P. sieversorum*; (**F**) *P. xiangchengensis*; (**G**) *P. mangshanensis* venoms. High- resolution image can be found in Figure S4.

The myotoxicity caused by viperid venom is mainly attributed to the basic Asp49 and Lys49 PLA_2_s and their homologues, which can damage the skeletal muscle cell plasma membrane [56,57]. Here, these two PLA_2_s could be found in both adult and subadult habu snake venoms (Supplementary Tables S1–S6), and thus can apparently cause higher creatine kinase activity (1598.6–2382.4 U/L) in mice compared to the saline-treated mice (612.2 U/L) (all *p* < 0.05) (Figure 3A). Although the abundance of these two PLA_2_s varied among adult habu snakes, Student’s t-tests indicated that their myotoxicities were relatively similar to each other (all *p* ≥ 0.14). Compared to the adult habu snake venoms, the subadult *P. mangshanensis* venom contained the lowest abundance of PLA_2_ (1.23%), but the highest apparent value of myotoxicity (2382.4 U/L) (Figure 2 and Figure 3A). Given that the habu snakes are notorious for causing severe muscle necrosis in victims, the gastrocnemius muscle of the ICR mice that were injected with these habu snake venoms was subsequently dissected and observed. Unsurprisingly, these mice suffered from significant swelling and varying degrees of muscle necrosis, which were roughly correlated with the intensity of creatine kinase activity: *P. xiangchengensis* and *P. mangshanensis* venoms caused the strongest submuscular symptoms with large reddish brown areas of muscle necrosis in mice, followed by *P. cornutus* and *P. jerdonii* venoms, whereas *P. mucrosquamatus* and *P. sieversorum* venoms caused relatively mild submuscular symptoms with small reddish brown areas of muscle necrosis in mice (Figure 4). Moreover, the subcutaneous capillaries near the injection site were also observed with varying degrees of bleeding, which could further verify the conclusion that the habu snake venom can induce strong hemorrhagic symptoms in victims [58,59,60]. In total, it could be inferred that the myotoxicity of these venoms is not rigidly correlated with the relative abundance of basic Asp49 and Lys49 PLA_2_s, but may be correlated with the potential capability of the unique catalytic domains of each PLA_2_ to damage the skeletal muscle cell plasma membrane. For instance, a basic Asp49 PLA_2_ (Q90W39) was highly expressed in *P. cornutus* venom, but the venom caused low myotoxicity according to the description in the UniProt database. In line with a previous study [12], the myotoxicity and enzymatic activity of PLA_2_s in these venoms were not correlated, as some acidic PLA_2_s showed a lack of myotoxicity and some myotoxic Lys49 PLA_2_s may be enzymatically inactive according to the UniProt database (Supplementary Tables S1–S6). Among the adult habu snake venoms, the *P. cornutus* venom expressed the highest amount of enzymatically active PLA_2_s (37.68%), followed by *P. jerdonii* (22.10%), *P. xiangchengensis* (10.56%), *P. mucrosquamatus* (8.47%), and *P. sieversorum* (0.36%) venoms; in addition, these venoms showed a strong dose-dependent relationship between hydrolytic activity towards soybean lecithin and the relative abundance of enzymatically active PLA_2_s with a strong Spearman’s rank correlation coefficient (*ρ* = 1.0) as well as a strong Pearson’s correlation coefficient (*R* = 0.94, *F*_1,3_ = 24.39, *p* = 0.016) (Supplementary Tables S1, S2 and S4–S6, and Figure 3B).

On the other hand, one-way ANOVA also revealed the interspecific variations in four other enzymatic activities of the venoms from five adult habu snakes (all *p* < 0.0001; Figure 3C–F). Specifically, the *P. sieversorum* venom expressed the highest capability (83.2 nM/min/mg) to degrade casein, and was nearly 2.7-fold more active than the *P. cornutus* venom, which expressed the lowest capability (30.9 nM/min/mg). Although SVMPs and SVSPs are generally deemed to be responsible for the proteolytic activity in viperid venoms [61,62], the relative abundance of these two components was weakly correlated with the capability of these habu snake venoms to degrade casein based on a moderate Spearman’s rank correlation coefficient (*ρ* = 0.60, *p* > 0.05). Among these five adult habu snake venoms, the *P. cornutus* and *P. sieversorum* venoms expressed the strongest LAAO (276.0 nM/min/mg) and 5′-NT (327.3 nM/min/mg) activities, respectively, and were about 2.3-fold and 3.8-fold more active than the *P. mucrosquamatus* (117.8 nM/min/mg) and *P. xiangchengensis* (87.1 nM/min/mg) venoms, which expressed the weakest activity in the corresponding enzymes. Except for the *P. sieversorum* venom (6.5 nM/min/mg), the four other adult habu snake venoms could apparently hydrolyze the synthetic substrate (BAPNA), with the *P. jerdonii* venom possessing the highest activity (111.9 nM/min/mg). However, nonparametric Spearman’s rank correlation analysis indicated that the variation in these enzymatic activities was not related to the differences in the relative abundance of the relevant venom components (all *p* > 0.05). This could be attributed to the potential bias in the estimation of the relative abundance of venom components, especially for those with trace or low abundances (e.g., LAAO and 5′-NT); in addition, it might also be related to the potential differences in the catalytic domains of the components from the same toxin family in these habu snake venoms, and the detailed mechanisms should be further explored.

Taken together, the interspecific differences in toxicological and enzymatic activities would potentially drive the variability between the local and systematic symptoms caused by these habu snake venoms, and therefore highlight the requirement of species-specific therapeutic schedules for the victims. Clinically, two heterologous monovalent antivenoms against *Gloydius brevicaudus* venom or *Deinagkistron actus* venom and a bivalent antivenom against venoms including *P. mucrosquamatus* venom were recommended to treat the victims envenomed by *P. mucrosquamatus* [58,63], and a species-specific monovalent antivenom against *P. flavoviridis* venom was available for the treatment of snakebites caused by *P. flavoviridis* [59], whereas the strict preclinical trials for these treatment are still insufficient [63], especially for those endemic to areas with few human inhabitants and recently classified habu snakes. Accordingly, preclinical evaluation of the immunological cross-reaction between these antivenoms and habu snake venoms should further be implemented using immunoassay methods, such as ELISA, Western blotting, and antivenomic strategy.

### 2.4. Estimation of the Phylogenetic Signal of the Interspecific Variations in Venom Traits

The proteomic profiles of venoms have been considered important complementary evidence in discriminating between related species with taxonomic controversy and in exploring the adaptive evolution trends in venom compositional patterns along with phylogenetic clues; thus, snake venom contains valuable information on the evolutionary history of venomous snakes [1,11,39,64,65,66]. The evaluation of the phylogeny-based evolutionary changes in venom traits (venom composition and function) of the habu snakes is shown in Table 1 (five species from the current study and four species from previous studies). Eight protein families (venom composition) with relative abundances higher than 1% in at least five of the nine taxa as well as an LD_50_ (venom function) were used in the analysis. Since the venom of *P. mangshanensis* was collected from subadult samples, the venom traits of this species were excluded from the analysis. Moreover, the major contribution of the variables that could not be well converged (less than 80%) on two vectors by principal component analysis, thus venom breadth was calculated as an alternatively integrative trait of venom composition for testing the potential phylogeny-related change. For venom composition, Blomberg’s analysis [67] revealed K values of eight venom protein families ranging from 0.324 (disintegrin) to 0.932 (SVSP) with a mean K value of 0.618 ± 0.194 (mean ± SD), and that of venom breadth was 0.472 (Table 3). The statistical result of 1000 times calculation on the trait values randomly assigned to the tree tips further indicated that, except for SVSP (*p* = 0.026), the other venom composition traits were not associated with phylogeny (all *p* ≥ 0.153, Table 3). Similar to the traits of venom composition, a moderate K value of 0.621 with a *p*-value of 0.311 for LD_50_ indicated no evidence for an association with phylogeny (Table 3).

Overall, the resemblance of the relatives within the genus *Protobothrops* in most venom traits deviated from Brownian motion evolution, which has been similarly observed in the genera *Agkistrodon* and *Sistrurus* [10,12]. It has been proposed that snake venom is a relatively labile evolutionary trait, and a small number of taxa and different adaptation patterns to environmental factors might play important roles in the random evolution of venom traits [12]. Another possibility is that the similarity in venom traits might be more a reflection of similar selection pressures driven by high similarities in diets, rather than the phylogenetic similarity of the snakes [12]. It is undeniable that variable habitats can probably drive the habu snakes to develop different adaption patterns to the same environmental factor, especially for those with a narrow niche space, such as *P. cornutus*, *P. xiangchengensis*, and *P. sieversorum*. On the other hand, detailed information on diets of these habu snakes has not yet been reported, but it has been empirically recorded that some of them (including *P. cornutus*, *P. jerdonii*, *P. mucrosquamatus*, and *P. xiangchengensis*) mainly feed on birds and rodents [68]; thus, the habu snakes might possess a relatively narrow but highly similar diet niche, which would imply high similar selection pressures on the venoms of the habu snakes. Therefore, precise verification using quantifiable dietary data of these habu snakes is required in the future. Unlike the genera *Agkistrodon* and *Sistrurus*, at least one toxin family (SVSP) in venoms of the genus *Protobothrops* was found to be associated with phylogeny in the current study. A recent investigation on *Bothrops atrox* venoms claimed that the lethality patterns of snake venom can be variable depending on the prey [69]; thus, different conclusions on phylogeny-related venom functions might be obtained if the lethality of the habu snake venoms were evaluated in birds, which should be conducted in future research. In brief, the habu snake venoms might have evolved under a combination of adaptive and neutral mechanisms.

Moreover, based on a protein-by-protein comparison, the apparent mean K value of these habu snakes was larger than those of the genera *Agkistrodon* (0.50 ± 0.28) [12] and *Sistrurus* (0.11 ± 0.06) [10], which means that the intensity of the phylogenetic signal in venom composition variation across habu snakes would be much higher than that across the two other genera. Nonparametric comparisons (Wilcoxon signed rank test) further indicated that the genera *Protobothrops* and *Agkistrodon* showed no difference in phylogenetic signals (*p* = 0.68), despite both genera expressing higher phylogenetic signals than the genus *Sistrurus* (all *p* < 0.05). A reasonable explanation is that the degree of covariation between phylogeny and venom variation is evolutionarily labile and varies among different clades of closely-related venomous snakes [12].

## 3. Conclusions

We employed a combined proteomic strategy to decomplex the venom composition of pit vipers from a subset of Old World habu snakes (*Protobothrops*) and revealed remarkable variations in the venom proteomes of these habu snakes, both in the presence or absence and the relative abundance of venom protein families. Specifically, 14 protein families were identified in all chromatographic fractions and gel bands of these habu snake venoms, and 11 of them were shared among these venoms. Moreover, the venoms from adult habu snakes were overwhelmingly dominated by SVMP (32.56 ± 13.94%), PLA_2_ (22.93 ± 9.26%), and SVSP (16.27 ± 4.79%), with a total abundance of higher than 65% in the venom proteomes. These adult habu snakes were estimated to share relatively low similarities between their venom proteomes, with an average protein similarity coefficient (PSC) of 17.7% (which ranged from 7.6% to 39.2%). Compared with these adult snakes, the subadult *P. mangshanensis* venom contained an extremely low abundance of PLA_2_ (1.23%), but a high abundance of CTL (51.47%), followed by SVMP (22.06%) and SVSP (10.90%). An analysis integrated with the proteomic profiles of the habu snake venoms in the current and previous studies further indicated that the highly abundant protein families had much lower degrees of variation in the relative abundance than the trace families. Interspecific variation in lethality and enzymatic activities was also explored in the habu snake venoms in the current study, and it was found to potentially drive the variability in the local and systematic symptoms of snakebites and highlighted the requirement of species-specific therapeutic schedules for victims. Apparent variations in muscle necrosis were observed in the mice injected with these habu snake venoms, but there were no variations in the degree of myotoxicity. With regard to the venoms from the less clinically important habu snakes, such as *P. cornutus*, *P. kelomohy*, *P. sieversorum* and *P. xiangchengensis*, their toxicities were relatively strong among all the venoms; therefore, the envenomations by these snakes, as well as their clinical treatment, should be given more attention. Phylogeny-based comparisons of the variations in venom traits revealed that, except for SVSP, the resemblance of the relatives within the genus *Protobothrops* in venom traits deviated from Brownian motion evolution. Given the potential similarity of the selection pressures due to diet on the venoms of the habu snakes, it was inferred that these venoms might have evolved under a combination of adaptive and neutral mechanisms. The mean K value of the venom traits from this subset of habu snakes was larger than those of the genera *Agkistrodon* and *Sistrurus*, which can further validate the assumption that the degree of covariation between phylogeny and venom variation is evolutionarily labile and varies among different clades of closely related venomous snakes.

## 4. Materials and Methods

### 4.1. Animals, Snake Venom Extraction, and Ethics

Six adult *P. jerdonii* (snout-vent length (SVL), 52.9–77.8 cm) and nine adult *P. mucrosquamatus* (SVL, 61.2–78.2 cm) were collected from fields in Zhejiang and Guangxi, China, respectively, and then transferred to our laboratory and maintained in Reptile Pet Terrariums (60 × 45 × 45 cm, Reptizoo, Dongguan, China). Two adult specimens of each species of *P. cornutus* (SVL, 51.2–52.5 cm), *P. sieversorum* (SVL, 65.8–67.5 cm), and *P. xiangchengensis* (SVL, 61.8–62.5 cm), as well as two subadult specimens of *P. mangshanensis* (19-month-old; considering the potential injuries to the animals, the provider did not allow the measurement of the morphological characteristics of these specimens), were supplied by two anonymous reptile enthusiasts, and return to them after venom extraction. Fresh venom of each snake was extracted by biting on a parafilm-wrapped jar, centrifuged at 10,000× *g* 4 °C for 15 min to remove the impurities, then lyophilized, weighed, pooled (in equal amounts by species), and stored at −80 °C. The handling of all snakes collected for venom extraction and the ICR mice from the Laboratory Animal Center of Hangzhou Normal University for evaluating the toxicological activity of venoms and experimental procedures were supervised and approved by the Animal Research Ethics Committee of Hangzhou Normal University (AREC20140311).

### 4.2. Proteomic Analyses

#### 4.2.1. Reverse-Phase High-Performance Liquid Chromatography (RP-HPLC)

A total of 5 mg crude venom powder was reconstituted in 0.1% TFA and centrifuged at 15,000× *g* at 4 °C for 10 min; the supernatant was then subjected to a Kromasil 300 RP-C18 column (250 × 4.6 mm, 5 μm; AkzoNobel) using a Waters E600 HPLC system (Waters, Milford, MA, USA). The venom proteins were separated at 1 mL/min using a linear gradient of the mobile phase A (0.1% TFA) and B (100% ACN) according to Gao et al. [62]: isocratically 10% B for 10 min, 10–15% B over 10 min, 15–45% B over 80 min, and 45–60% B over 50 min. The separation process was monitored at 215 nm. The eluted fractions were collected manually and concentrated in a CentriVap^®^ Centrifugal Concentrator (Labconco, Kansas, MO, USA).

#### 4.2.2. Sodium Dodecyl Sulfate-Polyacrylamide Gel Electrophoresis (SDS-PAGE)

The lyophilized fractions were re-dissolved in ddH_2_O, and the concentration of protein in each fraction was quantified according to Bradford [70]. Then, the proteins of each fraction were mixed with loading buffer, loaded into the wells, and performed with 12% and 18% SDS-PAGE under reducing conditions. After the electrophoresis, the gels were stained with 0.1% CBB R-250, and the protein bands were scanned using a Umax2100 densitometer system (Novax Technologies, Taipei, China).

#### 4.2.3. In-Gel and In-Solution Tryptic Digestion

The protein bands in the gels were excised and chopped into small pieces, sequentially incubated with 0.1 mM NH_4_HCO_3_ in 30% ACN and 0.1 mM NH_4_HCO_3_ at room temperature. The proteins in the gel pieces were reduced by incubation with 50 mM DTT in 0.1 M NH_4_HCO_3_, alkylated with 55 mM IAA in 0.1 M NH_4_HCO_3_, and then in-gel digested with trypsin (MS grade, Promega, Madison, WI, USA) for 20 h at 37 °C. Several chromatographic fractions with a low relative abundance were unsuitable for electrophoresis, and thus were re-dissolved, reduced, alkylated, and in-solution tryptic digested according to the procedure mentioned above. The peptide mixture in the supernatant was collected and lyophilized.

#### 4.2.4. Mass Spectrometry (MS) Identification

For MALDI-TOF-MS/MS analysis, a 2 μL peptide mixture re-dissolved in 20% ACN was loaded onto a stainless steel plate and air-dried, then covered with 0.5 μL of 5 mg/mL α-cyano-4-hydroxycinnamic acid (ABI) in 0.1% TFA and 50% ACN and air-dried again. Subsequently, the peptide mixture was subjected to a 4800 Plus MALDI-TOF/TOF-MS mass spectrometer (AB SCIEX, Framingham, MA, USA) according to the instruction manual. For LC-MS/MS analysis, the peptide mixture was re-dissolved in 0.1% TFA, automatically subjected to an Acclaim™ PepMap™ 100 C18 column (Trap Cartridge; 5 × 0.3 mm, 5 μm; ThermoFisher, Waltham, MA, USA) using the Ultimate 3000 RSLCnano system (ThermoFisher, Waltham, MA, USA). Then, the mixture was separated using an Acclaim™ PepMap™ RSLC 100 C18 column (NanoViper; 75 μm × 15 cm, 2 μm; ThermoFisher, Waltham, MA, USA) with the mobile phase A (0.1% FA) and B (20% ACN in 0.1% FA) under a linear gradient according to Zheng et al. [39]: 4% B over 3 min, 4–50% B over 47 min, 50–99% B over 4 min, and 99% B over 6 min. The eluents were sequentially loaded onto a Q Exactive Orbitrap platform (ThermoFisher, Waltham, MA, USA) according to the operation manual. Moreover, the chromatographic fractions with a short retention time could not be effectively visualized in the SDS-PAGE, and thus they were re-dissolved and directly subjected to LC-MS/MS analysis.

The raw MS/MS spectra were interpreted using FlexAnalysis 3.4 (Bruker Daltonics, Brerica, MA, USA) or Xcalibur 2.2 (ThermoFisher, Waltham, MA, USA), and the assignment of sequence similarity was executed using PEAKS X (Bioinformatics Solutions Inc., Waterloo, ON, Canada) against the UniProt Serpentes database or an in-house database composed of toxin transcripts from *P. jerdonii* and *P. mucrosquamatus* venom-gland transcriptomes. The fragment mass tolerances were separately set at 0.4 Da and 0.1 Da for MALDI-TOF/TOF-MS and LC-MS/MS, carbamidomethyl (C) and oxidation (M) were set as fixed and variable modifications, respectively.

#### 4.2.5. Protein Relative Abundance and Similarity Estimation

The relative abundance of each protein was evaluated according to our previous study [39]. Briefly, the relative abundance of each fraction was calculated using the integration of the chromatographic peaks in the RP-HPLC spectra by Empower software (Waters, Milford, MA, USA) and Origin 9.0 (OriginLab Corporation, Northampton, MA, USA), and that of each protein band was estimated using densitometry measurements of the SDS-PAGE gel by Gel-Pro Analyzer 4.0 software (Media Cybernetics, Rockville, MD, USA). If the fraction contains only one protein band in the SDS-PAGE gel, the proportion of the relevant chromatographic peak in the whole venom protein was directly recorded for the relative abundance; if the fraction contains more than one protein band in the SDS-PAGE gel, the proportion of each protein band was calculated by densitometry, and the chromatographic peak was proportionally assigned to each band for their relative abundance (relative abundance of each protein band in the fraction = (integration of the relevant chromatographic peak/accumulated integration of all chromatographic peaks × 100) × (optical density of each protein band/accumulated optical density of all protein bands in the relevant lane × 100)). Finally, the relative abundance of the bands belonging to the same protein family was accumulated to describe the proteomic profile of the venom. To estimate the similarity of venom proteins between each two (“a” and “b”) adult habu snakes, a protein similarity coefficient (PSC) was employed and calculated according to a previous study [1]: PSC_ab_ = (2 × (number of proteins shared between a and b)/(total number of distinct proteins in a + total number of distinct proteins in b)) × 100.

### 4.3. Toxicological and Enzymatic Activities of Venoms

#### 4.3.1. Lethality

For determination of the median lethal dose (LD_50_), groups of four male ICR mice (22–26 g) received injections of various doses of venom dissolved in 100 μL physiological saline by the intraperitoneal route as previously described [62], and the control group only received an injection of the same volume of physiological saline. The number of deaths occurring during a period of 24 h were recorded, and the LD_50_ (μg/g) was estimated using the Spearman–Karber method.

#### 4.3.2. Myotoxicity

For determination of the myotoxicity, each venom (10 μg in 20 μL physiological saline) was injected into the gastrocnemius muscle of the right hind limb in a group of four male ICR mice (22–26 g), with the controls only received an identical injection of physiological saline as previously described [12]. After 3 h, blood was collected from each mouse into a heparinized plastic tube, centrifugated at 3000× *g* at 4 °C for 10 min. The creatine kinase (CK) activity of the plasma was then determined using the CK Determination Kit (A032-1-1, Nanjing Jiancheng Bioengineering Institute, Nanjing, China) according to the instruction manual, and expressed as U/L.

#### 4.3.3. Phospholipase A_2_ Activity

The phospholipase A_2_ activity was evaluated according to Zheng et al. [39] with some modification. Specifically, each venom (0.8 μg in 1 μL 20 mM PBS) was quickly added into the substrate system (0.1 M NaCl, 10 mM CaCl_2_, 7 mM Triton X-100, 0.35% soybean lecithin, and 98.8 mM phenol red, pH 8.0). The absorbance was recorded at 558 nm at 28 °C for a 2.5 min interval. A change in absorbance of 0.3 OD value/min/μg venom was used to define one unit of phospholipase A_2_ activity.

#### 4.3.4. Proteolytic Activity

The proteolytic activity of each venom was determined according to Gao et al. [62] and He et al. [71]. Briefly, a substrate system (0.5 mL 2% casein in 0.2 M Tris–HCl, pH 8.5) was incubated with the venom (40 μg in 100 μL 20 mM PBS) at 37 °C for 2 h. The reaction was stopped by incubation with 0.6 mL 0.44 M TCA at 37 °C for 30 min; the mixture was then centrifuged at 12,000× *g* at 4 °C for 15 min. Aliquots (0.8 mL) of the supernatant were sequentially mixed with 2.0 mL 0.4 M Na_2_CO_3_ and 0.4 mL folin reagent (1:2 dilution), and the absorbance was recorded at 660 nm. l-Tyrosine was used as the standard, and the proteolytic activity was defined as nmol of l-Tyrosine released/min/mg venom.

#### 4.3.5. Esterolytic Activity

The esterolytic activity was tested according to the method from Tu et al. [72] with a slight modification. The substrate system (180 μL 1 mM BAPNA in 0.1 M Tris-HCl, pH 8.0) in the wells of a 96-well micro-plate was incubated with each venom (8 μg in 2 μL 20 mM PBS) at 37 °C for 30 min. Then, the reaction was stopped by adding 18 μL 30% acetic acid, and the absorbance of each well in the micro-plate was read at 405 nm. *p*-nitroaniline was used as the standard, and the esterolytic activity was defined as nmol of *p*-nitroaniline released/min/mg venom.

#### 4.3.6. l-Amino Acid Oxidase Activity

The l-amino acid oxidase activity was determined following the method of Toyama et al. [73]. The substrate solution (90 μL 50 mM Tris-HCl, pH 8.0, containing 0.25 mM l-Leucine, 2 mM o-phenylenediamine, and 0.81 U/mL horseradish peroxidase) in the wells of a 96-well micro-plate was incubated with the venom (0.8 μg in 1 μL 20 mM PBS) at 37 °C for 1 h. Then, the reaction was stopped by adding 50 μL 2 M H_2_SO_4_, and the absorbance was read at 490 nm. H_2_O_2_ was used as the standard, and the activity was expressed as nmol of H_2_O_2_ degraded/min/mg venom.

#### 4.3.7. 5′-Nucleotidase Activity

The 5′-nucleotidase activity was assayed following the method of Dhananjaya et al. [74]. The substrate system (90 μL 50 mM Tris–HCl, pH 7.4, containing 10 mM MgCl_2_, 50 mM NaCl, 10 mM KCl, and 10 mM 5′ AMP) was incubated with each venom (0.8 μg in 10 μL 20 mM PBS) at 37 °C for 30 min. Then, the reaction was stopped by adding 100 μL 0.42% ammonium molybdate in 1 M sulfuric acid and 10% ascorbic acid, and the absorbance was read at 660 nm. KH_2_PO_4_ was as the standard, and the activity was defined as nmol of inorganic phosphate released/min/mg venom.

#### 4.3.8. Statistical Analyses

The LD_50_ with a 95% confidence interval was performed using the Trimmed Spearman–Karber 1.5 program. Student’s t-test, one-way ANOVA, and Tukey’s test were used to analyze the myotoxicity and enzymatic activities based on Statistica 8.0 (StatSoft Inc., Tulsa, OK, USA); nonparametric Spearman’s rank correlation analysis and linear correlation analysis were further employed to assess the correlation between the enzymatic activities and the relative abundance of the relevant venom components. Descriptive statistics were defined as mean ± standard deviation (SD), and the significance level was set at α = 0.05.

### 4.4. Phylogeny-Based Comparative Analyses

The phylogeny-based comparative exploration of variations in venom traits of habu snakes in Table 1 was carried out following the methods advanced by Gibbs et al. [10] and Lomonte et al. [12]. For estimation of the phylogenetic relationships between these habu snakes, *Azemiops feae* and *Gloydius brevicaudus* were selected as outgroups; the venom proteomes of these two species were decomplexed in our previous studies [39,62,75] and can be used in the current study. Three mtDNA genes including 12S RNA, 16S RNA, and cytochrome b genes were used for constructing a Bayesian inference tree. These mtDNA genes were mainly screened from a recent study [76] and the NCBI database and were assigned individual samples based on the location from which the snake was collected; the accession numbers were listed in Table S7. Briefly, the sequences were aligned and merged using Geneious 4.8.3 (Biomatters Ltd., Auckland, New Zealand), and the best-fitting model was screened using PartitionFinder 1.1.1 [77] based on the BIC and greedy search. The phylogeny was then constructed using BEAST 2.2 [78], and the maximum clade credibility tree was obtained using TreeAnnotator 2.2. Then, the Bayesian phylogeny in Newick format was deposited in Data File S1. Venom traits for each taxon were represented by the relative abundance of eight protein families, the venom complexity, and the LD_50_. The venom complexity, defined as venom breadth, was calculated using the standardized Levin index [7,79].

The multiPhylosignal subroutine in the R-package Picante [80] was performed to test the phylogenetic signal of variation in the venom traits of the habu snakes. For each venom trait, Blomberg’s K analysis [67] based on the multiPhylosignal program generated a K value, which can vary from 0 (under random evolution, trait variation is independent of phylogeny) to 1 (under Brownian evolution, trait variation is correlated with the amount of phylogenetic divergence) to greater than 1 (trait is very conserved in evolution). Whether the observed K was greater than random expectation was analyzed by a statistical test of 1000 times random calculation based on the trait values randomly assigned to the tree tips.

## Figures and Tables

**Table 3:** Estimation of phylogenetic signal (K-value) for the variation in venom traits of the habu snakes excluding *P. mangshanensis* as shown in Table 1.

Venom Trait	K-Value	*p*-Value
SVMP	0.566	0.405
SVSP	0.932	0.026
PLA_2_	0.728	0.158
CRISP	0.789	0.153
Disintegrin	0.324	0.694
BPP/CNP	0.474	0.422
CTL	0.628	0.303
LAAO	0.499	0.504
Venom breadth	0.472	0.492
LD_50_	0.621	0.311

## Data Availability

Not applicable.

## Supplementary Materials

The following supporting information can be downloaded at https://www.mdpi.com/article/10.3390/toxins15050350/s1. Table S1: Assignment of the reverse-phase chromatographic fractions from *Protobothrops cornutus* venom to protein families by nESI-MS/MS of selected peptide ions from in-gel digested protein bands separated by SDS-PAGE; Table S2: Assignment of the reverse-phase chromatographic fractions from *Protobothrops jerdonii* venom to protein families by MALDI-TOF-MS/MS and nESI-MS/MS of selected peptide ions from in-gel digested protein bands separated by SDS-PAGE; Table S3: Assignment of the reverse-phase chromatographic fractions from *Protobothrops mangshanensis* venom to protein families by nESI-MS/MS of selected peptide ions from in-gel digested protein bands separated by SDS-PAGE; Table S4: Assignment of the reverse-phase chromatographic fractions from *Protobothrops mucrosquamatus* venom to protein families by nESI-MS/MS of selected peptide ions from in-gel digested protein bands separated by SDS-PAGE; Table S5: Assignment of the reverse-phase chromatographic fractions from *Protobothrops sieversorum* venom to protein families by nESI-MS/MS of selected peptide ions from in-gel digested protein bands separated by SDS-PAGE; Table S6: Assignment of the reverse-phase chromatographic fractions from *Protobothrops xiangchengensis* venom to protein families by MALDI-TOF-MS/MS and nESI-MS/MS of selected peptide ions from in-gel digested protein bands separated by SDS-PAGE; SVMP, snake venom metalloproteinase; SVSP, snake venom serine proteinase; PLA_2_, phospholipase A_2_; CRISP, cysteine-rich secretory protein; BPP/CNP, bradykinin-potentiating and C-type natriuretic peptides; CTL, C-type lectin; LAAO, L-amino acid oxidase; VEGF, vascular endothelial growth factor; NGF, nerve growth factor; PDE, phosphodiesterase; 5′NT, 5′-nucleotidase; PLB, phospholipase B; QC, glutaminyl-peptide cyclotransferase. Unknown: unidentified components; Table S7: GenBank accession numbers of the sequences for mitochondrial gene markers from snakes for phylogenetic relationship reconstruction; Data File S1: Bayesian phylogeny in Newick format for the snakes in Table S7; Figures S1–S4: High-resolution images for Figure 1, Figure 2, Figure 3 and Figure 4 in the text.
