# Supplementary material for: Phylogeny-Related Variations in Venomics: A Test in a Subset of Habu Snakes (Protobothrops)
Source: Toxins (Basel). 2023 May 21;15(5):350. doi: 10.3390/toxins15050350 (PMC10223207; doi:10.3390/toxins15050350)
Supplement: Supplementary file 1 [file toxins-15-00350-s001.zip › Supplementary tables 1-6.pdf]

Supplementary table 1 Assignment of the reverse-phase chromatographic fractions from *Protobothrops cornutus* venom to protein families by nESI-MS/MS of selected peptide ions from in-gel digested protein bands separated by SDS-PAGE.

**Peak**	**%**	**MW (kDa)**	**Peptide Ion**		**Score**	**MS/MS-derived sequence**	**Protein family/species/accession**
			**m/*z***	***z***			
1	0.03	*de novo*	255.7	2	55	PPPIS	BPP/CNP; *Protobothrops jerdonii*; ~PJD|c31701
2	0.75	*de novo*	248.6	2	71	QSKPG	BPP/CNP; *Protobothrops flavoviridis*; ~P0C7P5
		de novo	255.7	2	59	PPISP	
3	3.05	-	622.3	2	104	QSKPGRPPPISP	BPP/CNP; *P. jerdonii*; ~PJD|c31701
		-	475.8	2	47	RPPHHIPP	
4	2.75	*de novo*	241.1	2	80	QSKPG	BPP/CNP; *P. flavoviridis*; ~P0C7P5
		de novo	255.7	2	60	PPISP	
5	0.18	-	670.6	3	79	GDWNDDRCTGQSADCPR	Disintegrin; *Protobothrops mucrosquamatus*; ~E9NW26
6	1.90	14.5	1005.4	2	79	GDWNDDRCTGQSADCPR	Disintegrin; *P. mucrosquamatus*; ~E9NW26
7	0.98	11.9	670.6	3	64	GDWNDDRCTGQSADCPR	Disintegrin; *P. mucrosquamatus*; ~E9NW26
			984.4	2	49	LSPGAQCADGLCCDQCR	
8	0.44	-	576.2	2	58	CTGQSADCPR	Disintegrin; *P. mucrosquamatus*; ~E9NW26
			984.4	2	55	LSPGAQCADGLCCDQCR	
9	0.12	32.5	978.7	3	66	AAESECDIAESCTGQSAECPTDDFQR	SVMP-fragment; *P. mucrosquamatus*; ~XP_015682631
			801.3	3	57	LRPGTQCEDGECCEQCQFK	
			559.8	2	53	LFCVQGPIGK	
			623.9	2	52	LFCVQGPIGKK	
			514.3	2	43	IPCAPQDVK	
			467.6	3	42	IPCAPQDVKCGR	
			578.3	2	42	KIPCAPQDVK	
10	0.51	16.0	456.8	2	36	ETLVPILK	VEGF; *P. jerdonii*; ~PJD|c48748_g1
11	0.75	17.4	502.5	3	54	CCFVHDCCYGK	PLA_2; *P. mucrosquamatus*; ~Q90W39
			591.3	2	45	AAAVCLGHNLR	
12	0.01	28.9	-	-	-	-	Unknown
	2.72	17.3	657.1	4	95	KNAIPFYSSYGCYCGWGGQGKPK	PLA_2; *P. mucrosquamatus*; ~Q90W39
			1249.1	2	87	NAIPFYSSYGCYCGWGGQGKPK	
			1019.1	3	86	NAIPFYSSYGCYCGWGGQGKPKDATDR	
			769.6	3	79	CCFVHDCCYGKLTDCSPK	
		12.8	838.7	3	69	TGVIICGEGTECEKQICECDR	PLA_2; *P. flavoviridis*; ~Q805A2
			776.9	2	57	TGVIICGEGTECEK	
13	4.77	17.4	657.1	4	85	KNAIPFYSSYGCYCGWGGQGKPK	PLA_2; *P. mucrosquamatus*; ~Q90W39
			833.0	3	80	NAIPFYSSYGCYCGWGGQGKPK	
			764.6	4	67	NAIPFYSSYGCYCGWGGQGKPKDATDR	
			577.5	4	63	CCFVHDCCYGKLTDCSPK	
			591.3	2	57	AAAVCLGHNLR	

Peak	%	MW (kDa)	Peptide Ion		Score	MS/MS-derived sequence	Protein family/species/accession
			m/*z*	*z*			
		13.1	838.7	3	102	TGVIICGEGTECEKQICECDR	PLA$_2$; *P. flavoviridis*; ~Q805A2
			776.9	2	66	TGVIICGEGTECEK	
14	8.75	17.4	1019.1	3	127	NAIPFYSSYGCYCGWGGQGKPKDATDR	PLA$_2$; *P. mucrosquamatus*; ~Q90W39
			657.1	4	91	KNAIPFYSSYGCYCGWGGQGKPK	
			833.0	3	86	NAIPFYSSYGCYCGWGGQGKPK	
			769.6	3	83	CCFVHDCCYGKLTDCSPK	
			591.3	2	81	AAAVCLGHNLR	
		13.1	1032.4	2	73	DATDRCCFVHDCCYGK	PLA$_2$; *P. mucrosquamatus*; ~Q90W39
			591.3	2	69	AAAVCLGHNLR	
			577.5	4	49	CCFVHDCCYGKLTDCSPK	
			502.5	3	44	CCFVHDCCYGK	
		11.2	591.3	2	72	AAAVCLGHNLR	PLA$_2$; *P. mucrosquamatus*; ~Q90W39
			657.1	4	60	KNAIPFYSSYGCYCGWGGQGKPK	
			502.5	3	50	CCFVHDCCYGK	
			833.0	3	43	NAIPFYSSYGCYCGWGGQGKPK	
15	3.73	28.4	1722.7	1	86	TNCPASCFCHNEII	CRISP; *P. jerdonii*; ~Q7ZZN9
		21.2	583.3	2	51	NVDFDSESPR	CRISP; *P. flavoviridis*; ~1WVR_A
	0.25	17.3	591.3	2	68	AAAVCLGHNLR	PLA$_2$; *P. mucrosquamatus*; ~Q90W39
			577.5	4	67	CCFVHDCCYGKLTDCSPK	
			657.1	4	58	KNAIPFYSSYGCYCGWGGQGKPK	
			833.0	3	46	NAIPFYSSYGCYCGWGGQGKPK	
16	0.46	50.1	596.8	2	42	IMGWGTTTPTK	SVSP; *Crotalus scutulatus*; ~AUS82544
		42.9	756.8	2	72	VIGGDECNINEHR	SVSP; *Gloydius blomhoffii*; ~Q9PT51
	0.04	31.1	777.3	2	57	MEWYPEAAANAER	CRISP; *P. flavoviridis*; ~1WVR_A
			583.3	2	49	NVDFDSESPR	
			769.3	2	46	MEWYPEAAANAER	
			589.3	2	41	SVNPTASNMLK	
		28.4	611.8	2	49	GNVDFDSESPR	CRISP; *Vipera berus nikolskii*; ~B7FDI0
	0.05	19.9	560.6	3	78	FIRIDAACVCVISR	NGF; *P. mucrosquamatus*; ~XP_015667549
			556.3	2	72	NPNPVPTGCR	
			632.3	2	72	IDAACVCVISR	
			697.3	2	59	ALTMEGKQASWR	
			714.3	2	54	CRNPNPVPTGCR	
			594.6	3	41	HWNSYCTTTHTFVK	
			962.5	1	39	QYFFETK	
	0.28	17.4	591.3	2	71	AAAVCLGHNLR	PLA$_2$; *P. mucrosquamatus*; ~Q90W39
17	0.22	33.9	559.8	2	35	TLCAGILEGGK	SVSP; *P. mucrosquamatus*; ~PMR|c29163_g4
	19.07	18.9	1505.5	1	91	CCFVHDCCYGK	PLA$_2$; *Trimeresurus stejnegeri*; ~P82896

Peak	%	MW (kDa)	Peptide Ion m/z	Peptide Ion z	Score	MS/MS-derived sequence	Protein family/species/accession
			871.9	2	82	EICECDKAAAICFR	
		15.7	838.7	3	83	TGVIICGEGTECEKQICECDR	PLA$_2$; *P. flavoviridis*; ~Q805A2
			776.9	2	80	TGVIICGEGTECEK	
18	0.15	57.9	565.3	2	51	ILCAGILQGGK	SVSP; *P. flavoviridis*; ~BAN82037
			423.2	2	47	RMPAQSR	
		46.4	756.8	2	72	VIGGDECNINEHR	SVSP; *Agkistrodon contortrix contortrix*; ~P09872
	0.02	28.4	611.8	2	55	GNVDFDSESPR	CRISP; *V. berus nikolskii*; ~B7FDI0
	1.09	19.4	871.9	2	61	EICECDKAAAICFR	PLA$_2$; *T. stejnegeri*; ~P82896
19	2.52	57.4	550.8	2	50	NVPNEDEQR	SVSP; *T. stejnegeri*; ~Q8AY80
			419.5	3	49	NVPNEDEQRR	
		50.5	716.0	3	61	SAHIAPLSLPSNPPSVGSVCR	SVSP; *A. contortrix contortrix*; ~JAV51428
			679.3	2	37	EWVLTAAHCDR	
20	5.61	34.8	559.3	2	48	TLCAGILQGGK	SVSP; *P. jerdonii*; ~Q9DF66
		33.9	738.9	2	58	DDENDKDIMLIR	SVSP; *Crotalus atrox*; ~AUS82489
			498.2	3	46	DDENDKDIMLIR	
		32.0	492.9	3	81	DDENDKDIMLIR	SVSP; *C. atrox*; ~AUS82489
			498.2	3	57	DDENDKDIMLIR	
		19.4	492.9	3	60	DDENDKDIMLIR	SVSP; *C. atrox*; ~AUS82489
			722.4	2	39	VLNEDEQTRDPK	
		16.0	756.8	2	62	VIGGDECNINEHR	SVSP; *Gloydius ussuriensis*; ~AAP20637
21	1.70	58.4	756.8	2	56	VIGGDECNINEHR	SVSP; *G. blomhoffii*; ~Q9PT51
		35.7	763.8	2	77	IIGGDECNINEHR	SVSP; *Gloydius halys*; ~4E7N_A
			573.3	2	48	EKFFCLSSK	
		33.9	749.8	2	79	VVGGDECNINEHR	SVSP; *A. contortrix contortrix*; ~P82981
	0.89	16.2	811.0	3	65	VFQQQMNWADAEKFCTQQR	CTL; *P. jerdonii*; ~D1MGU1
			805.7	3	65	VFQQQMNWADAEKFCTQQR	
			829.8	2	57	TTDNQWWSMDCSK	
			808.4	2	49	YNAWSAESECIASK	
22	0.44	56.1	756.8	2	66	VIGGDECNINEHR	SVSP; *G. blomhoffii*; ~CAB65936
		42.7	749.8	2	81	VVGGDECNINEHR	SVSP; *Ovophis okinavensis*; ~AAB34084
		33.9	500.2	3	61	VVGGDECNINEHR	SVSP; *A. contortrix contortrix*; ~P82981
	0.60	17.9	811.0	3	63	VFQQQMNWADAEKFCTQQR	CTL; *P. jerdonii*; ~D1MGU1
			829.8	2	48	TTDNQWWSMDCSK	
			797.9	2	43	VFQQQMNWADAEK	
			808.4	2	40	YNAWSAESECIASK	
23	0.79	76.2	674.8	4	82	SECDIAESCTGQSADCPTDHYHR	SVMP; *P. mucrosquamatus*; ~XP_029142019
			577.3	3	80	YSEDLDYGMVDHGTK	
			742.6	3	73	LHSWVECESGECCEQCR	

Peak	%	MW (kDa)	Peptide Ion		Score	MS/MS-derived sequence	Protein family/species/accession
			m/*z*	*z*			
		56.5	1013.4	2	63	LTPGSQCAEGLCCEQCR	SVMP; *P. mucrosquamatus*; ~XP_029142018
			549.3	2	48	FSTEGKLCR	
			664.4	2	44	YIELVIVADHR	
		43.8	734.7	3	92	HDNAQLLTGMIFNEKIEGR	SVMP; *P. mucrosquamatus*; ~XP_029142018
			1013.4	2	73	LTPGSQCAEGLCCEQCR	
			643.0	4	71	FSDCSLNQYSNEIIHNHFTSR	
			556.1	4	65	IYETANDLNEIYRPLKIR	
			729.4	3	63	HDNAQLLTGMIFNEKIEGR	
			664.4	2	61	YIELVIVADHR	
			549.3	2	56	FSTEGKLCR	
			583.0	3	56	HDNAQLLTGMIFNEK	
			532.3	2	50	TRPHLVANR	
			651.3	3	46	IYETANDLNEIYRPLK	
			414.9	3	37	YDGDKTEISSK	
		38.7	1013.4	2	70	LTPGSQCAEGLCCEQCR	SVMP; *T. stejnegeri*; ~P0DM87
			549.3	2	51	FSTEGKLCR	
	0.60	33.2	718.4	3	97	IMGWGTISSTKETYPNVPR	SVSP; *Gloydius intermedius*; ~ASX97880
			713.0	3	76	IMGWGTISSTKETYPNVPR	
	0.89	18.2	591.3	2	57	FCSEQVKGAR	CTL; *P. jerdonii*; ~D1MGU0
24	3.99	-	618.8	2	77	SAGQLFEESLR	LAAO; *P. mucrosquamatus*; ~XP_015673892
			455.6	3	61	SAGQLFEESLRK	
			583.4	2	54	ITFRPPLPPK	
		-	618.4	2	70	KVTVTYQTPAK	LAAO; *P. flavoviridis*; ~BAN82013
			554.3	2	65	VTVTYQTPAK	
	0.08	-	725.4	2	95	VVSLNVLCTECR	5'NT; *Gloydius brevicaudus*; ~B6EWW8
			653.9	2	86	QVPVVQAYAFGK	
	0.24	-	640.2	3	70	DCPSDWSSYEGHCYR	CTL; *Trimeresurus albolabris*; ~P81114
			584.3	2	52	EQFECLVSR	
25	0.94	110.0	545.8	2	59	YGPVSGEIIR	PDE; *Protobothrops elegans*; ~BAP39928
			746.8	2	48	SVQPQAQSWSCSK	
		81.9	782.4	2	60	CSSITELEKVNQR	PDE; *P. elegans*; ~BAP39928
			561.3	2	48	NPFYTPSPAK	
			545.8	2	46	YGPVSGEIIR	
			516.8	2	38	YCLLHQAK	
			403.2	2	38	ALEMADR	
			457.8	2	35	LNLNNQAK	
			678.3	2	33	AATYFWPGSEVK	
	0.17	59.7	618.8	2	64	SAGQLFEESLR	LAAO; *P. mucrosquamatus*; ~XP_015673893

Peak	%	MW (kDa)	Peptide Ion m/z	z	Score	MS/MS-derived sequence	Protein family/species/accession
			438.7	2	61	STTDLPSR	
			501.2	2	46	DVNRASENP	
			474.8	2	35	IVREYIR	
	5.37	27.0	655.4	2	46	KPQCILNAPLR	SVMP; *P. jerdonii*; ~Q7ZZS9
	1.18	17.3	829.8	2	53	TTDNQWWSMDCSK	CTL; *P. jerdonii*; ~D1MGU1
			808.4	2	53	YNAWSAESECIASK	
			811.0	3	47	VFQQQMNWADAEKFCTQQR	
			805.9	2	43	VFQQQMNWADAEK	
		16.0	591.3	2	55	WVNVYCGQR	CTL; *P. mucrosquamatus*; ~XP_015671571
			655.3	2	53	KWVNVYCGQR	
			466.2	3	52	TCLGLEKGTDFR	
			457.2	2	40	FCMEQAK	
26	0.05	112.0	545.8	2	59	YGPVSGEIIR	PDE; *P. elegans*; ~BAP39928
			746.8	2	47	SVQPQAQSWSCSK	
	1.06	77.6	899.4	3	89	SECDIAESCTGQSADCPTDHYHR	SVMP; *P. mucrosquamatus*; ~XP_029142019
			577.3	3	71	YSEDLDYGMVDHGTK	
		27.9	587.8	2	47	MIPCAPQDVK	SVMP; *P. elegans*; ~BAP39935
			552.3	2	43	CGKLYCFR	
	0.11	57.4	412.6	3	58	K.KVTVTYQTPAK.D	LAAO; *P. flavoviridis*; ~BAN82013
			626.8	2	49	K.SAGQLYEESLR.K	
	0.06	50.5	597.8	2	69	TPVPAGCYDSK	PLB; *P. elegans*; ~BAP39922
			450.9	3	57	RTPVPAGCYDSK	
			453.2	2	52	VADISMAAK	
			555.3	2	48	YSDQTNILR	
	0.98	17.9	811.0	3	96	VFQQQMNWADAEKFCTQQR	CTL; *P. jerdonii*; ~D1MGU1
			805.7	3	78	VFQQQMNWADAEKFCTQQR	
			829.8	2	66	TTDNQWWSMDCSK	
			808.4	2	59	YNAWSAESECIASK	
			532.2	3	46	VFQQQMNWADAEK	
			805.9	2	44	VFQQQMNWADAEK	
		16.5	591.3	2	66	WVNVYCGQR	CTL; *P. mucrosquamatus*; ~XP_015671571
			466.2	3	52	TCLGLEKGTDFR	
27	0.26	-	555.3	2	63	YSDQTNILR	PLB; *P. elegans*; ~BAP39922
			597.8	2	63	TPVPAGCYDSK	
	2.88	-	640.2	3	100	DCPSDWSSYEGHCYR	CTL; *T. albolabris*; ~P81114
			584.3	2	58	EQFECLVSR	
		-	808.4	2	63	YNAWSAESECIASK	CTL; *P. jerdonii*; ~D1MGU1
			805.9	2	49	VFQQQMNWADAEK	

Peak	%	MW (kDa)	Peptide Ion		Score	MS/MS-derived sequence	Protein family/species/accession
			m/*z*	*z*			
28	1.74	64.4	559.8	2	50	LFCVQGPIGK	SVMP; *P. mucrosquamatus*; ~XP_015682631
			623.9	2	45	LFCVQGPIGKK	
			514.3	2	44	IPCAPQDVK	
			1467.6	2	37	AAESECDIAESCTGQSAECPTDDFQR	
		54.7	520.3	2	45	FKAAGTECR	SVMP; *P. flavoviridis*; ~Q90ZI3
			816.3	3	38	AAGTECRAATDECDMADLCTGR	
	2.31	17.9	1216.1	2	83	VFQQQMNWADAEKFCTQQR	CTL; *P. jerdonii*; ~D1MGU1
			829.8	2	63	TTDNQWWSMDCSK	
			808.4	2	54	YNAWSAESECIASK	
			532.2	3	43	VFQQQMNWADAEK	
			805.9	2	42	VFQQQMNWADAEK	
		16.3	811.0	3	75	VFQQQMNWADAEKFCTQQR	CTL; *P. jerdonii*; ~D1MGU1
			829.8	2	48	TTDNQWWSMDCSK	
			805.9	2	41	VFQQQMNWADAEK	
			539.2	3	35	YNAWSAESECIASK	
29	5.82	63.4	978.7	3	79	AAESECDIAESCTGQSAECPTDDFQR	SVMP; *P. mucrosquamatus*; ~XP_015682631
			559.8	2	64	LFCVQGPIGK	
			623.9	2	62	LFCVQGPIGKK	
		53.8	843.3	2	65	AATDECDMADLCTGR	SVMP; *P. flavoviridis*; ~Q90ZI3
			567.9	3	55	AATDECDMADLCTGR	
		43.4	843.3	2	60	AATDECDMADLCTGR	SVMP; *P. flavoviridis*; ~Q90ZI3
			851.3	2	47	AATDECDMADLCTGR	
		34.6	843.3	2	62	AATDECDMADLCTGR	SVMP; *P. flavoviridis*; ~Q90ZI3
			567.9	3	50	AATDECDMADLCTGR	
		33.2	843.3	2	61	AATDECDMADLCTGR	SVMP; *P. flavoviridis*; ~Q90ZI3
			567.9	3	44	AATDECDMADLCTGR	
		29.1	559.8	2	60	LFCVQGPIGK	SVMP; *P. mucrosquamatus*; ~XP_015682631
			623.9	2	54	LFCVQGPIGKK	
		22.8	532.6	3	62	YIPCAQEDVKCGR	SVMP; *Bothrops jararacussu*; ~Q1PHZ4
			574.9	3	58	ENEKYIPCAQEDVK	
	3.52	17.8	1208.1	2	110	VFQQQMNWADAEKFCTQQR	CTL; *P. jerdonii*; ~D1MGU1
			1216.1	2	98	VFQQQMNWADAEKFCTQQR	
			1658.7	1	71	TTDNQWWSMDCSK	
			571.3	4	61	KESHLVSFESSEEVDFVVSK	
			808.4	2	61	YNAWSAESECIASK	
			532.2	3	49	VFQQQMNWADAEK	
			805.9	2	47	VFQQQMNWADAEK	
		16.2	805.7	3	82	VFQQQMNWADAEKFCTQQR	CTL; *P. jerdonii*; ~D1MGU1

Peak	%	MW (kDa)	Peptide Ion		Score	MS/MS-derived sequence	Protein family/species/accession
			m/*z*	*z*			
			811.0	3	80	VFQQQMNWADAEKFCTQQR	
			829.8	2	56	TTDNQWWSMDCSK	
			808.4	2	51	YNAWSAESECIASK	
			805.9	2	39	VFQQQMNWADAEK	
			797.9	2	35	VFQQQMNWADAEK	
30	0.32	84.7	823.3	3	78	RSECDIAESCTGHSDDCPTDR	SVMP; *P. mucrosquamatus*; ~XP_015685802
			551.2	5	72	SECDIAESCTGHSDDCPTDRFHR	
			771.3	3	67	SECDIAESCTGHSDDCPTDR	
		70.5	742.6	3	69	LHSWVECESGECCEQCR	SVMP; *P. mucrosquamatus*; ~XP_015685802
			617.7	4	62	RSECDIAESCTGHSDDCPTDR	
			771.3	3	60	SECDIAESCTGHSDDCPTDR	
			551.2	5	60	SECDIAESCTGHSDDCPTDRFHR	
			467.6	3	49	IPCAPQDVKCGR	
	0.64	17.4	811.0	3	82	VFQQQMNWADAEKFCTQQR	CTL; *P. jerdonii*; ~D1MGU1
			805.7	3	61	VFQQQMNWADAEKFCTQQR	
			797.9	2	42	VFQQQMNWADAEK	
			539.2	3	40	YNAWSAESECIASK	
		16.2	640.2	3	49	DCPSDWSSYEGHCYR	CTL; *T. albolabris*; ~P81114
31	0.48	52.4	1148.4	2	43	SVCDLPEQCTGQSADCPTDR	SVMP; *P. mucrosquamatus*; ~XP_015683144
	0.24	17.4	805.7	3	58	VFQQQMNWADAEKFCTQQR	CTL; *P. jerdonii*; ~D1MGU1
			811.0	3	57	VFQQQMNWADAEKFCTQQR	
			808.4	2	51	YNAWSAESECIASK	
			829.8	2	47	TTDNQWWSMDCSK	
			797.9	2	40	VFQQQMNWADAEK	
			805.9	2	38	VFQQQMNWADAEK	
		16.0	811.0	3	62	VFQQQMNWADAEKFCTQQR	CTL; *P. jerdonii*; ~D1MGU1
			808.4	2	55	YNAWSAESECIASK	
32	0.46	64.8	551.3	4	98	HDNAQLLTGMIFNEKIEGR	SVMP; *P. mucrosquamatus*; ~XP_015681821
			583.0	3	64	HDNAQLLTGMIFNEK	
			769.4	2	62	IYESVNALNVIFR	
			729.4	3	50	HDNAQLLTGMIFNEKIEGR	
			453.7	2	50	YHFVANR	
			505.8	2	46	SVGIVQDHR	
		63.0	734.7	3	80	HDNAQLLTGMIFNEKIEGR	SVMP; *P. mucrosquamatus*; ~XP_015681821
			769.4	2	64	IYESVNALNVIFR	
			583.0	3	64	HDNAQLLTGMIFNEK	
			851.0	3	62	DSCSCGANSCIMSETVSNEPSSR	
			505.8	2	49	SVGIVQDHR	

Peak	%	MW (kDa)	Peptide Ion m/*z*	*z*	Score	MS/MS-derived sequence	Protein family/species/accession
			453.7	2	47	YHFVANR	
			437.9	3	46	FIELVIVADHR	
			575.3	2	36	SRYHFVANR	
			729.4	3	35	HDNAQLLTGMIFNEKIEGR	
		58.8	734.7	3	91	HDNAQLLTGMIFNEKIEGR	SVMP; *P. mucrosquamatus*; ~XP_015681821
			729.4	3	76	HDNAQLLTGMIFNEKIEGR	
			577.6	3	65	HDNAQLLTGMIFNEK	
			583.0	3	61	HDNAQLLTGMIFNEK	
			769.4	2	60	IYESVNALNVIFR	
			453.7	2	49	YHFVANR	
			656.4	2	49	FIELVIVADHR	
			575.3	2	48	SRYHFVANR	
			505.8	2	45	SVGIVQDHR	
			851.0	3	42	DSCSCGANSCIMSETVSNEPSSR	
		54.7	551.3	4	89	HDNAQLLTGMIFNEKIEGR	SVMP; *P. mucrosquamatus*; ~XP_015681821
			547.3	4	80	HDNAQLLTGMIFNEKIEGR	
			851.0	3	69	DSCSCGANSCIMSETVSNEPSSR	
			577.6	3	68	HDNAQLLTGMIFNEK	
			583.0	3	65	HDNAQLLTGMIFNEK	
			656.4	2	62	FIELVIVADHR	
			629.7	3	61	RHDNAQLLTGMIFNEK	
			453.7	2	58	YHFVANR	
			505.8	2	56	SVGIVQDHR	
			635.0	3	56	RHDNAQLLTGMIFNEK	
			845.7	3	46	DSCSCGANSCIMSETVSNEPSSR	
			583.8	2	44	RSVGIVQDHR	
		43.4	729.4	3	111	HDNAQLLTGMIFNEKIEGR	SVMP; *P. mucrosquamatus*; ~XP_015681821
			734.7	3	94	HDNAQLLTGMIFNEKIEGR	
			684.3	3	85	LRPGAQCAEGLCCDQCR	
			629.7	3	74	RHDNAQLLTGMIFNEK	
			865.9	2	74	HDNAQLLTGMIFNEK	
			769.4	2	70	IYESVNALNVIFR	
			476.5	4	68	RHDNAQLLTGMIFNEK	
			851.0	3	68	DSCSCGANSCIMSETVSNEPSSR	
			579.6	3	66	MAHELGHNLGLHHDR	
			873.9	2	64	HDNAQLLTGMIFNEK	
			585.0	3	63	MAHELGHNLGLHHDR	
			656.4	2	59	FIELVIVADHR	

Peak	%	MW (kDa)	Peptide Ion m/z	Peptide Ion z	Score	MS/MS-derived sequence	Protein family/species/accession
			575.3	2	58	SRYHFVANR	
			453.7	2	58	YHFVANR	
			845.7	3	54	DSCSCGANSCIMSETVSNEPSSR	
			490.0	3	53	RFIELVIVADHR	
			505.8	2	47	SVGIVQDHR	
			583.8	2	42	RSVGIVQDHR	
		19.0	684.3	3	115	LRPGAQCAEGLCCDQCR	SVMP; *P. mucrosquamatus*; ~XP_015681821
			551.3	4	102	HDNAQLLTG<u>M</u>IFNEKIEGR	
			769.4	2	71	IYESVNALNVIFR	
			583.0	3	65	HDNAQLLTG<u>M</u>IFNEK	
			577.6	3	58	HDNAQLLTGMIFNEK	
			729.4	3	52	HDNAQLLTGMIFNEKIEGR	
			505.8	2	51	SVGIVQDHR	
			437.9	3	47	FIELVIVADHR	
			453.7	2	44	YHFVANR	

Methionine oxidation is underlined.

Supplementary table 2 Assignment of the reverse-phase chromatographic fractions from *Protobothrops jerdonii* venom to protein families by MALDI-TOF-MS/MS and nESI-MS/MS of selected peptide ions from in-gel digested protein bands separated by SDS-PAGE.

Peak	%	MW (kDa)	Peptide Ion		Score	MS/MS-derived sequence	Protein family/species/accession
			m/*z*	*z*			
1	0.09	-	422.0	4	69	RLPKSKGASATSAASRP	BPP/CNP; *Protobothrops flavoviridis*; ~P0C7P5
			493.0	4	63	HRLPKSKGASATSAASRP*M*	
			687.7	3	59	AAPHRLPKSKGASATSAASRP	
			456.3	4	56	HRLPKSKGASATSAASRP	
			981.5	2	49	ASGPAAPHRLPKSKGASATSA	
			401.2	3	45	ASGPAAPHRLPK	
			406.6	3	41	SGPAAPHRLPKS	
2	0.18	-	410.7	2	40	GRPPPISP	BPP/CNP; *Protobothrops mucrosquamatus*; ~XP_05680879
			655.4	3	25	HHIPPLLVQQWAQGSPR	
			566.8	2	30	SKPGRPPPISP	
			367.2	3	20	LKGLGKKAVGE	
			523.3	2	28	KPGRPPPISP	
			699.4	2	40	HQSKPGRPPPISP	
			529.3	2	44	QQWAQGSPR	
3	1.34	-	459.3	2	22	PGRPPPISP	BPP/CNP; *Protobothrops elegans*; ~M5A7M5
			523.3	2	34	KPGRPPPISP	
			410.7	2	34	GRPPPISP	
4	5.32	-	835.4	3	101	ASGPAAPHRLPKSKGASATSAASRPM	BPP/CNP; *P. flavoviridis*; ~BAP39952
			811.8	3	94	SGPAAPHRLPKSKGASATSAASRPM	
			676.9	4	62	SGPAAPHRLPKSKGASATSAASRPMRD	
			676.9	4	58	EAASGPAAPHRLPKSKGASATSAASRPM	
			548.8	4	42	AAPHRLPKSKGASATSAASRPM	
5	0.76	18.7	1151.4	1	5	CTGQSADCPR	Disintegrin; *Protobothrops jerdonii*; Q7ZZS9
			1920.7	1	83	GDNPDDRCTGQSADCPR	
			1967.8	1	85	LSPGAQCADGLCCDQCR	
6	0.44	19.0	1015.4	1	36	ARGDNPDDR	Disintegrin; *P. jerdonii*; Q7ZZS9
			1151.5	1	66	CTGQSADCPR	
			1920.7	1	68	GDNPDDRCTGQSADCPR	
			1967.8	1	139	LSPGAQCADGLCCDQCR	
7	5.09	9.4	451.2	2	38	QLELNER	VEGF; *Bitis gabonica*; ~AAR06855
8	5.20	9.4	2011.8	1	73	LTPGSQCAEGLCCDQCR	Disintegrin; *P. jerdonii*; P0C6E4
9	2.09	18.9	1180.5	1	13	CTGLSDDCPR	Disintegrin; *P. flavoviridis*; ~P23323
			2011.8	1	88	LTPGSQCAEGLCCDQCR	
10	3.53	19.3	1180.5	1	51	CTGLSDDCPR	Disintegrin; *P. jerdonii*; P0C6E4
			1211.5	1	39	FARGDYPDDR	

Peak	%	MW (kDa)	Peptide Ion		Score	MS/MS-derived sequence	Protein family/species/accession
			m/z	z			
			1998.8	1	90	GDYPDDRCTGLSDDCPR	
			2011.8	1	106	LTPGSQCAEGLCCDQCR	
11	2.10	19.2	666.9	3	83	GDYPDDRCTGLSDDCPR	Disintegrin; *P. jerdonii*; P0C6E4
			404.5	3	58	FARGDYPDDR	
12	0.51	19.4	666.9	3	81	GDYPDDRCTGLSDDCPR	Disintegrin; *P. jerdonii*; P0C6E4
			404.5	3	55	FARGDYPDDR	
		14.4	2011.8	1	110	LTPGSQCAEGLCCDQCR	Disintegrin; *P. flavoviridis*; ~P23323
		10.9	2011.8	1	113	LTPGSQCAEGLCCDQCR	Disintegrin; *P. flavoviridis*; ~P23323
13	0.01	31.3	1004.4	1	42	GNDYGYCR	SVMP fragment; *P. elegans*; ~BAP39935
			1083.5	1	35	DSCFKGNQK	
			1132.5	1	25	GNDYGYCRK	
	0.62	19.0	666.9	3	68	GDYPDDRCTGLSDDCPR	Disintegrin; *P. jerdonii*; P0C6E4
			606.3	2	52	FARGDYPDDR	
		14.7	1006.4	2	56	LTPGSQCAEGLCCDQCR	Disintegrin; *P. jerdonii*; P0C6E4
			666.9	3	40	GDYPDDRCTGLSDDCPR	
14	0.12	32.3	1004.4	1	49	GNDYGYCR	SVMP fragment; *P. elegans*; ~BAP39935
			1083.5	1	36	DSCFKGNQK	
			1132.5	1	24	GNDYGYCRK	
		29.7	1058.5	1	56	IACEPQNVK	SVMP fragment; *Deinagkistrodon acutus*; ~ALE20769
			1812.7	1	134	QGAQCAEGLCCDQCR	
			2053.8	1	125	LKQGAQCAEGLCCDQCR	
		28.5	1025.5	1	55	FKGAGTECR	SVMP fragment; *Gloydius halys*; ~Q8AWI5
			1058.5	1	38	IACEPQNVK	
			1269.5	1	37	SAECTDRFQR	
			1812.7	1	96	QGAQCAEGLCCDQCR	
	0.02	16.7	1116.5	1	54	LDIYSYSQK	PLA_2; *P. jerdonii*; Q8JIY9
			1505.5	1	69	CCFVHDCCYGK	
			1567.6	1	77	NEDIVCGGGTECEK	
			2063.8	1	49	DATDRCCFVHDCCYGK	
15	0.09	28.0	1001.4	1	48	GKGNFYCR	SVMP fragment; *P. mucrosquamatus*; ~XP_0568263
			1118.6	1	38	LFCVQGPIGK	
			1155.6	1	30	KIPCAPQDVK	
			2401.9	1	31	LRPGTQCEDGECCEQCQFK	
			2934.1	1	215	AAESECDIAESCTGQSAECPTDDFQR	
16	0.18	20.5	962.4	1	56	QYFFETK	NGF; *Gloydius intermedius*; ~ALB06112
			1003.4	1	60	EDHPVHNR	
			1111.5	1	73	NPNPVPTGCR	
			1263.6	1	83	IDAACVCVISR	

Peak	%	MW (kDa)	Peptide Ion		Score	MS/MS-derived sequence	Protein family/species/accession
			m/*z*	*z*			
			1427.6	1	27	CRNPNPVPTGCR	
			1781.8	1	120	HWNSYCTTTHTFVK	
	0.08	16.1	910.5	1	37	IEVMQFK	VEGF; *P. mucrosquamatus*; ~Q330K6
			1315.6	1	35	EHTACECRPR	
			2207.0	1	48	IEVMQFKEHTACECRPR	
	0.02	11.0	1505.5	1	35	CCFVHDCCYGK	PLA_2; *Crotalus durissus cumanensis*; ~P86805
17	8.11	16.7	1741.8	1	117	QICECDKAAAICFR	PLA_2; *Trimeresurus stejnegeri*; ~Q6H3C5
	0.16	12.1	910.5	1	20	IEVMQFK	VEGF; *P. mucrosquamatus*; ~Q330K6
			1315.5	1	17	EHTACECRPR	
18	0.44	21.3	1116.5	1	61	LDIYSYSQK	PLA_2; *P. jerdonii*; Q8JIY9
			1505.5	1	73	CCFVHDCCYGK	
			1567.6	1	99	NEDIVCGGGTECEK	
			2063.8	1	61	DATDRCCFVHDCCYGK	
			2665.2	1	129	LDIYSYSQKNEDIVCGGGTECEK	
		16.7	1116.6	1	30	LDIYSYSQK	PLA_2; *P. jerdonii*; Q8JIY9
			1505.5	1	56	CCFVHDCCYGK	
			1670.8	1	79	AAAICFLDNLGTYNK	
			2214.0	1	8	EPLTTYLFYACYCGWGGR	
			2700.2	1	38	EATGKEPLTTYLFYACYCGWGGR	
	0.01	10.4	-	-	-	-	Unknown
19	6.92	15.8	1116.5	1	66	LDIYSYSQK	PLA_2; *P. jerdonii*; Q8JIY9
			1505.5	1	74	CCFVHDCCYGK	
			1567.6	1	92	NEDIVCGGGTECEK	
			1670.8	1	89	AAAICFLDNLGTYNK	
20	0.70	34.8	980.4	1	44	QICECDR	PLA_2; *P. flavoviridis*; ~BAP39949
			1148.5	1	40	SDIYSYSWK	
			1206.6	1	88	AAAVCFGQNLR	
			1552.7	1	96	TGVIICGEGTECEK	
			1577.6	1	68	CCFVHDCCYEK	
			2514.1	1	180	TGVIICGEGTECEKQICECDR	
		28.8	980.4	1	21	QICECDR	PLA_2; *P. flavoviridis*; ~S6BAM8
			1148.5	1	5	SDIYSYSWK	
			1206.6	1	61	AAAVCFGQNLR	
			1552.7	1	79	TGVIICGEGTECEK	
			1577.6	1	65	CCFVHDCCYEK	
		21.5	980.4	1	43	QICECDR	PLA_2; *P. flavoviridis*; ~BAP39949
			1148.5	1	46	SDIYSYSWK	

Peak	%	MW (kDa)	Peptide Ion m/z	z	Score	MS/MS-derived sequence	Protein family/species/accession
			1206.6	1	88	AAAVCFGQNLR	
			1552.7	1	100	TGVIICGEGTECEK	
			1577.6	1	75	CCFVHDCCYEK	
			2135.8	1	57	DATDRCCFVHDCCYEK	
			2514.1	1	160	TGVIICGEGTECEKQICECDR	
		20.5	799.9	2	106	AAAVCFGQNLRTYK	PLA$_2$; *P. flavoviridis*; ~Q805A2
			723.3	3	103	QICECDRAAAVCFGQNLR	
			402.9	3	86	AAAVCFGQNLR	
			629.3	4	84	TGVIICGEGTECEKQICECDR	
			776.9	2	76	TGVIICGEGTECEK	
		15.0	980.4	1	43	QICECDR	PLA$_2$; *P. flavoviridis*; ~BAP39949
			1206.6	1	88	AAAVCFGQNLR	
			1552.7	1	96	TGVIICGEGTECEK	
			1577.5	1	57	CCFVHDCCYEK	
21	0.44	47.8	929.5	1	56	LQFGLHSK	SVSP; *T. stejnegeri*; ~Q7QI7
			1117.6	1	64	TLCAGILQGGK	
	0.29	21.5	637.9	3	49	VHDCCYEKLTDCSPK	PLA$_2$; *P. flavoviridis*; ~BAN82002
		14.4	784.3	2	80	NEDIVCGGGTECEK	PLA$_2$; *P. jerdonii*; Q8JIY9
			1333.1	2	67	LDIYSYSQKNEDIVCGGGTECEK	
			835.9	2	66	AAAICFLDNLGTYNK	
			1285.1	2	53	AAAICFLDNLGTYNKEYNNYSK	
22	0.71	51.0	929.5	1	62	LQFGLHSK	SVSP; *T. stejnegeri*; ~Q7QI7
			1117.6	1	45	TLCAGILQGGK	
		41.2	929.5	1	59	LQFGLHSK	SVSP; *T. stejnegeri*; ~Q71QJ1
			1189.6	1	38	WDKDIMLIR	
			1205.6	1	22	WDKDIMLIR	
	1.45	28.7	1165.5	1	94	NVDFDSESPR	CRISP; *Echis coloratus*; ~P0DMT4
			1537.6	1	61	MEWYPEAAANAER	
			1553.6	1	52	MEWYPEAAANAER	
			2075.1	1	127	KPEIQNEIIDLHNSLRR	
23	0.75	39.0	583.3	2	56	NVDFDSESPR	CRISP; *P. flavoviridis*; ~6IMF_A
			581.3	2	45	SVNPTASNMLK	
			769.3	2	42	MEWYPEAAANAER	
		28.8	781.4	4	90	GNVDFDSESPRKPEIQNEIIDLHNSLR	CRISP; *Vipera berus nikolskii*; ~B7FDI0
			611.8	2	64	GNVDFDSESPR	
	2.77	12.7	1505.5	1	51	CCFVHDCCYGK	PLA$_2$; *Trimeresurus borneensis*; ~Q2YHJ2
			1741.8	1	65	QICECDKAAAICFR	
			2063.8	1	46	DATDRCCFVHDCCYGK	

Peak	%	MW (kDa)	Peptide Ion m/z	z	Score	MS/MS-derived sequence	Protein family/species/accession
24	0.50	45.0	929.5	1	54	LQFGLHSK	SVSP; *P. flavoviridis*; ~A0A077LD42
			1129.6	1	68	FLVALYTFR	
			1188.6	1	51	WNKDIMLIR	
			1204.6	1	21	MKLQFGLHSK	
	2.43	19.9	1505.5	1	73	CCFVHDCCYGK	PLA_2; *Trimeresurus hageni*; ~A0A0H3UX6
			1728.7	1	125	EVCECDKAAAICFR	
25	1.89	49.6	929.5	1	53	LQFGLHSK	SVSP; *T. stejnegeri*; ~Q7QI6
			1204.7	1	15	MKLQFGLHSK	
			1722.8	1	134	TLCAGVLQGGTDTCNR	
		41.0	929.5	1	54	LQFGLHSK	SVSP; *P. flavoviridis*; ~A0A077LD42
			1129.6	1	69	FLVALYTFR	
			1188.7	1	55	WNKDIMLIR	
			1204.6	1	33	WNKDIMLIR	
		31.5	1129.6	1	63	FLVALYTFR	SVSP; *P. jerdonii*; B0ZT25
			1172.6	1	46	VFDHLDWIK	
			1188.6	1	59	WNKDIMLIR	
			1204.6	1	5	WNKDIMLIR	
			2055.0	1	137	IQLGMHSTNVINEDVQTR	
			2071.0	1	82	IQLGMHSTNVINEDVQTR	
	0.39	16.1	967.4	1	42	EVCECDR	PLA_2; *P. flavoviridis*; ~T2HPU3
			1505.6	1	61	CCFVHDCCYGK	
			2063.8	1	71	DATDRCCFVHDCCYGK	
26	0.02	57.4	-	-	-	-	Unknown
	0.36	38.6	968.5	1	15	FFCPNRK	SVSP; *P. flavoviridis*; ~U3TAG3
			1097.5	1	30	EKFFCPNR	
		27.3	1129.6	1	40	FLVALYTFR	SVSP; *P. jerdonii*; B0ZT25
			2055.0	1	5	IQLGMHSTNVINEDVQTR	
			2071.0	1	77	IQLGMHSTNVINEDVQTR	
			2536.2	1	85	ETHPDVPYCANINILDYSVCR	
		20.7	-	-	-	-	Unknown
27	6.06	34.3	968.4	1	18	FFCPNRK	SVSP; *T. stejnegeri*; ~Q91516
			1097.5	1	44	EKFFCPNR	
			2506.1	1	134	EIYPDVPHCANINILDHAVCR	
			2879.4	1	189	LDSSVSNSEHIAPLSLPSSPPSVGSVCR	
			3046.4	1	122	TIPTKEIYPDVPHCANINILDHAVCR	
		29.7	1204.6	1	10	WNKDIMLIR	SVSP; *P. elegans*; ~P84788
			2119.1	1	158	SAHIAPLSLPSSPPSVGSVCR	
		26.8	1188.6	1	53	WNKDIMLIR	SVSP; *P. mucrosquamatus*; ~Q91510

Peak	%	MW (kDa)	Peptide Ion		Score	MS/MS-derived sequence	Protein family/species/accession
			m/*z*	*z*			
			1204.6	1	36	WNKDIMLIR	
			2054.1	1	139	VMGWGTITSPQVTLPDVPR	
			2119.1	1	141	SAHIAPLSLPSSPPSVGSVCR	
			2247.2	1	141	KSAHIAPLSLPSSPPSVGSVCR	
28	6.89	49.6	1035.5	2	35	VMGWGTITSPQVTLPDVPR	SVSP; *P. mucrosquamatus*; ~Q91508
		35.0	436.2	3	53	NMEIYLGVHSK	SVSP; *P. elegans*; ~P84788
			645.8	2	51	NMEIYLGVHSK	
		32.2	820.4	2	80	CANINLLDYEVCR	SVSP; *P. mucrosquamatus*; ~Q91510
			690.7	3	66	VMGWGTITSPQVTLPDVPR	
			685.4	3	48	VMGWGTITSPQVTLPDVPR	
		30.7	1188.6	1	67	WNKDIMLIR	SVSP; *D. acutus*; ~Q9I8X0
			2119.0	1	152	SAHIAPLSLPSSPPSVGSVCR	
			2247.1	1	164	KSAHIAPLSLPSSPPSVGSVCR	
		28.8	645.8	2	59	NMEIYLGVHSK	SVSP; *P. elegans*; ~P84788
			653.8	2	59	NMEIYLGVHSK	
			641.8	2	48	INLLDYSECR	
		27.2	1035.5	2	94	VMGWGTITSPQVTLPDVPR	SVSP; *P. mucrosquamatus*; ~Q9508
			1027.5	2	75	VMGWGTITSPQVTLPDVPR	
			646.3	2	59	DMEIYLGVHSK	
			436.5	3	54	DMEIYLGVHSK	
			820.4	2	43	CANINLLDYEVCR	
29	3.68	56.5	1152.7	1	83	AAKPELPAISR	SVSP; *P. flavoviridis*; ~T2HQA7
			2119.2	1	165	SAHIAPLSLPSSPPSVGSVCR	
		51.9	1037.5	1	82	GAHAGLPATSR	SVSP; *Agkistrodon piscivoru*; ~A0A94ARH4
			1189.6	1	75	WDKDIMLIR	
		35.2	968.5	1	23	FFCPNRK	SVSP; *P. flavoviridis*; ~U3TAG3
			1097.5	1	27	EKFFCPNR	
		33.2	1205.6	1	54	WDKDIMLIR	SVSP; *Gloydius brevicaudus*; ~P8509
			1512.7	1	92	VIGGDECNINEHR	
		30.8	2903.5	1	193	LDSPVSNSEHIAPLSLPSSPPSVGSICR	SVSP; *Crotalus oreganus helleri*; ~TE7I9
	0.13	42.1	-	-	-	-	Unknown
		22.8	-	-	-	-	Unknown
		14.7	-	-	-	-	Unknown
30	0.33	63.0	618.8	2	68	SAGQLFEESLR	LAAO; *P. elegans*; ~BAP3995
			682.9	2	67	SAGQLFEESLRK	
			490.3	2	46	EYLIKEGK	
		55.6	618.8	2	70	SAGQLFEESLR	LAAO; *P. elegans*; ~BAP3995
			682.9	2	67	SAGQLFEESLRK	

Peak	%	MW (kDa)	Peptide Ion m/z	Peptide Ion z	Score	MS/MS-derived sequence	Protein family/species/accession
			490.3	2	52	EYLIKEGK	
			588.6	4	42	YPVKPSEEGKSAGQLFEESLR	
		48.2	618.8	2	61	SAGQLFEESLR	LAAO; *P. elegans*; ~BAP3995
			682.9	2	60	SAGQLFEESLRK	
			490.3	2	50	EYLIKEGK	
		28.2	1058.0	2	82	RFDEIVGGMDQLPTSMYR	LAAO; *P. elegans*; ~BAP3995
			588.6	4	73	YPVKPSEEGKSAGQLFEESLR	
			711.0	3	68	RFDEIVGGMDQLPTSMYR	
			682.9	2	59	SAGQLFEESLRK	
			653.6	3	57	FDEIVGGMDQLPTSMYR	
			711.0	3	54	RFDEIVGGMDQLPTSMYR	
			988.0	2	48	FDEIVGGMDQLPTSMYR	
			716.3	3	44	RFDEIVGGMDQLPTSMYR	
			490.3	2	41	EYLIKEGK	
		25.3	711.0	3	76	RFDEIVGGMDQLPTSMYR	LAAO; *P. elegans*; ~BAP3995
			705.7	3	75	RFDEIVGGMDQLPTSMYR	
			711.0	3	62	RFDEIVGGMDQLPTSMYR	
			618.8	2	62	SAGQLFEESLR	
			682.9	2	57	SAGQLFEESLRK	
			988.0	2	49	FDEIVGGMDQLPTSMYR	
			716.3	3	46	RFDEIVGGMDQLPTSMYR	
			490.3	2	45	EYLIKEGK	
		21.9	618.8	2	74	SAGQLFEESLR	LAAO; *P. elegans*; ~BAP3995
			682.9	2	70	SAGQLFEESLRK	
			784.4	3	67	YPVKPSEEGKSAGQLFEESLR	
			716.3	3	63	RFDEIVGGMDQLPTSMYR	
			988.0	2	60	FDEIVGGMDQLPTSMYR	
			490.3	2	47	EYLIKEGK	
	0.37	41.9	1512.7	1	58	VIGGDECNINEHR	SVSP; *G. brevicaudus*; ~P85109
		34.2	2506.2	1	114	EIYPDVPHCANINILDHAVCR	SVSP; *T. stejnegeri*; ~Q91516
			2879.4	1	110	LDSSVSNSEHIAPLSLPSSPPSVGSVCR	
		30.7	2119.1	1	122	SAHIAPLSLPSSPPSVGSVCR	SVSP; *P. mucrosquamatus*; ~Q91509
		22.7	987.5	1	45	EIYPNVPR	SVSP; *C. oreganus helleri*; ~TDE97
			1347.7	1	11	AAYPWWPVTTR	
		21.2	987.5	1	22	EIYPNVPR	SVSP; *C. oreganus helleri*; ~TDE97
	0.27	13.3	666.9	3	74	GDYPDDRCTGLSDDCPR	SVMP; *P. jerdonii*; P0C6E4
			590.7	2	55	CTGLSDDCPR	

Peak	%	MW (kDa)	Peptide Ion		Score	MS/MS-derived sequence	Protein family/species/accession
			m/z	z			
			604.8	2	52	LSVGLVQDYSK	
		10.7	1006.4	2	47	LTPGSQCAEGLCCDQCR	SVMP; *P. jerdonii*; P0C6E4
			625.6	3	37	IHNDADSTASISACDGLK	
31	0.38	65.3	1449.7	1	101	VVSLNVLCTECR	5'NT; *P. flavoviridis*; ~T2HRS9
			1555.8	1	119	IIALGHSGFLEDQR	
			1577.9	1	98	HANFPILSANIRPK	
			2421.2	1	155	FHECNLGNLICDAVIYNNVR	
			3125.6	1	136	ETPVLSNPGPYLEFRDEVEELQNHANK	
		22.8	725.4	2	63	VVSLNVLCTECR	5'NT; *P. elegans*; ~BAP39925
			573.6	3	54	ETPVLSNPGPYLEFR	
			526.6	3	53	HANFPILSANIRPK	
			653.9	2	47	QVPVVQAYAFGK	
			513.2	2	44	QDCYGGVAR	
			430.2	2	42	IINVGSEK	
	0.31	56.1	948.6	1	29	IVREYIR	LAAO; *P. elegans*; ~A0A077L6L4
			1178.7	1	42	INFRPPLPPK	
			1236.6	1	94	SAGQLFEESLR	
			1306.8	1	37	INFRPPLPPKK	
			1334.8	1	36	RINFRPPLPPK	
		45.0	1176.1	2	75	YPVKPSEEGKSAGQLFEESLR	LAAO; *P. elegans*; ~BAP3995
			634.8	2	70	SGTKIFLTCNK	
			557.0	4	68	NCADIVMNDLSLIHQLPKK	
			412.9	3	68	SAGQLFEESLR	
			682.9	2	61	SAGQLFEESLRK	
			699.7	3	53	NCADIVMNDLSLIHQLPK	
			490.3	2	46	EYLIKEGK	
		41.4	431.9	3	57	HDDIFAYEKR	LAAO; *P. elegans*; ~BAP3995
			705.7	3	47	RFDEIVGGMDQLPTSMYR	
			711.0	3	47	RFDEIVGGMDQLPTSMYR	
			581.6	3	43	DPGVLKYPVKPSEEGK	
			716.3	3	30	RFDEIVGGMDQLPTSMYR	
			582.3	3	30	KDIQAFCYPSMIQK	
			634.3	3	29	DTSLVTADYVIVCTTSR	
			618.8	2	29	SAGQLFEESLR	
			711.0	3	28	RFDEIVGGMDQLPTSMYR	
			757.9	2	28	ETDYEEFLEIAR	
			577.0	3	27	KDIQAFCYPSMIQK	
			699.7	3	27	NCADIVMNDLSLIHQLPK	

Peak	%	MW (kDa)	Peptide Ion		Score	MS/MS-derived sequence	Protein family/species/accession
			m/z	z			
			800.9	2	23	DIQAFCYPSMIQK	
			438.7	2	23	STTDLPSR	
			569.3	2	21	HDDIFAYEK	
			682.9	2	21	SAGQLFEESLRK	
		33.8	618.8	2	75	SAGQLFEESLR	LAAO; *P. elegans*; ~BAP3995
			784.4	3	69	YPVKPSEEGKSAGQLFEESLR	
			682.9	2	63	SAGQLFEESLRK	
		30.7	711.0	3	70	RFDEIVGGMDQLPTSMYR	LAAO; *P. elegans*; ~BAP3995
			682.9	2	67	SAGQLFEESLRK	
			711.0	3	67	RFDEIVGGMDQLPTSMYR	
			716.3	3	64	RFDEIVGGMDQLPTSMYR	
			490.3	2	50	EYLIKEGK	
			988.0	2	44	FDEIVGGMDQLPTSMYR	
			423.6	3	31	SGTKIFLTCNK	
			512.3	2	31	IFLTCNKK	
			422.7	2	26	AIEEKVR	
			664.3	3	25	FDEIVGGMDQLPTSMYR	
			448.2	2	24	IFLTCNK	
			699.7	3	23	NCADIVMNDLSLIHQLPK	
		28.8	581.6	3	68	DPGVLKYPVKPSEEGK	LAAO; *P. elegans*; ~BAP3995
			618.8	2	68	SAGQLFEESLR	
			711.0	3	66	RFDEIVGGMDQLPTSMYR	
			757.9	2	52	ETDYEEFLEIAR	
			682.9	2	51	SAGQLFEESLRK	
			716.3	3	49	RFDEIVGGMDQLPTSMYR	
			784.4	3	46	YPVKPSEEGKSAGQLFEESLR	
			577.0	3	45	KDIQAFCYPSMIQK	
			438.7	2	42	STTDLPSR	
			532.7	2	41	NPLEECFR	
			550.9	3	37	NDKEGWYANLGPMR	
			490.3	2	37	EYLIKEGK	
			582.3	3	37	KDIQAFCYPSMIQK	
			705.7	3	35	RFDEIVGGMDQLPTSMYR	
			508.6	3	34	YDTYSTKEYLIK	
			634.3	3	33	DTSLVTADYVIVCTTSR	
			556.3	3	32	NDKEGWYANLGPMR	
			474.8	2	32	IVREYIR	
			988.0	2	28	FDEIVGGMDQLPTSMYR	

Peak	%	MW (kDa)	Peptide Ion m/z	z	Score	MS/MS-derived sequence	Protein family/species/accession
			567.3	2	27	YPVKPSEEGK	
			422.7	2	26	AIEEKVR	
			501.2	2	25	DVNRASE	
			589.9	2	21	INFRPPLPPK	
			448.2	2	21	IFLTCNK	
			512.3	2	20	IFLTCNKK	
			808.9	2	15	DIQAFCYPSMIQK	
		21.6	711.0	3	74	RFDEIVGGMDQLPTSMYR	LAAO; *P. elegans*; ~BAP3995
			618.8	2	62	SAGQLFEESLR	
			682.9	2	60	SAGQLFEESLRK	
			577.0	3	56	KDIQAFCYPSMIQK	
			567.3	2	55	YPVKPSEEGK	
			423.6	3	53	SGTKIFLTCNK	
			463.6	3	49	KFWEDDGIHGGK	
			705.7	3	49	RFDEIVGGMDQLPTSMYR	
			438.7	2	49	STTDLPSR	
			530.3	4	48	FWEDDGIHGGKSTTDLPSR	
			711.0	3	47	RFDEIVGGMDQLPTSMYR	
			716.3	3	46	RFDEIVGGMDQLPTSMYR	
	0.02	26.5	1290.7	1	51	NMEIYLGVHSK	SVSP; *P. elegans*; ~P84788
			2119.1	1	129	SAHIAPLSLPSSPPSVGSVCR	
			2247.2	1	74	KSAHIAPLSLPSSPPSVGSVCR	
	0.28	25.2	1292.6	1	65	DYYQTFLTNK	SVMP; *P. jerdonii*; Q7ZZS9
			1309.7	1	65	KPQCILNAPLR	
			2130.0	1	111	LFSDCSKDYYQTFLTNK	
			2147.1	1	64	DLITVQADAPTTAGLFGDWR	
	0.09	16.1	993.5	1	52	SYVWIGLR	CTL; *P. flavoviridis*; ~Q7LZ7
			1410.7	1	92	TCLGLEKETGFR	
	0.14	10.7	-	-	-	-	Unknown
32	0.15	109.1	540.9	3	65	KSVQPQAQSWSCSK	PDE; *P. elegans*; ~BAP39928
			746.8	2	50	SVQPQAQSWSCSK	
		104.9	1180.5	2	40	EQSSPLSCPFGPVPSPDVSGCR	PDE; *P. flavoviridis*; ~BAN82023
	1.57	57.4	948.6	1	18	IVREYIR	LAAO; *P. elegans*; ~A0A077L6L4
			1178.7	1	31	INFRPPLPPK	
			1236.6	1	92	SAGQLFEESLR	
			1306.8	1	14	INFRPPLPPKK	
			1334.8	1	17	RINFRPPLPPK	
			1364.7	1	10	SAGQLFEESLRK	

Peak	%	MW (kDa)	Peptide Ion m/*z*	*z*	Score	MS/MS-derived sequence	Protein family/species/accession
		55.1	711.0	3	72	RFDEIVGGMDQLPTSMYR	LAAO; *P. elegans*; ~BAP3995
			618.8	2	64	SAGQLFEESLR	
			711.0	3	64	RFDEIVGGMDQLPTSMYR	
			682.9	2	52	SAGQLFEESLRK	
			490.3	2	45	EYLIKEGK	
		34.7	618.8	2	53	SAGQLFEESLR	LAAO; *P. mucrosquamatus*; ~XP_05673893
			438.7	2	39	STTDLPSR	
33	0.03	106.3	540.9	3	62	KSVQPQAQSWSCSK	PDE; *P. elegans*; ~BAP39928
			746.8	2	53	SVQPQAQSWSCSK	
	0.11	57.0	705.7	3	71	RFDEIVGGMDQLPTSMYR	LAAO; *P. elegans*; ~BAP3995
			711.0	3	59	RFDEIVGGMDQLPTSMYR	
			577.0	3	53	KDIQAFCYPSMIQK	
			682.9	2	52	SAGQLFEESLRK	
			423.6	3	49	SGTKIFLTCNK	
			711.0	3	46	RFDEIVGGMDQLPTSMYR	
			490.3	2	44	EYLIKEGK	
	8.59	34.7	604.8	2	57	LSVGLVQDYSK	SVMP; *P. jerdonii*; P0C6E4
			686.9	2	51	LAIVVDYGMYTK	
			590.7	2	49	CTGLSDDCPR	
			694.9	2	44	LAIVVDYGMYTK	
		25.3	933.5	1	69	DSDKITVR	SVMP; *P. jerdonii*; P0C6E4
			2569.3	1	120	NDYQTFLTNYKPQCILNAPLR	
			3406.6	1	158	LFSDCSKNDYQTFLTNYKPQCILNAPLR	
		16.7	694.9	2	41	LAIVVDYGMYTK	SVMP; *P. jerdonii*; P0C6E4
			604.8	2	37	LSVGLVQDYSK	
			1006.4	2	32	LTPGSQCAEGLCCDQCR	
			666.9	3	32	GDYPDDRCTGLSDDCPR	
			466.6	3	31	SHDCAHLLTDTK	
			590.7	2	28	CTGLSDDCPR	
		11.0	1006.4	2	66	LTPGSQCAEGLCCDQCR	SVMP; *P. jerdonii*; P0C6E4
			666.9	3	64	GDYPDDRCTGLSDDCPR	
			604.8	2	60	LSVGLVQDYSK	
			686.9	2	59	LAIVVDYGMYTK	
			466.6	3	57	SHDCAHLLTDTK	
			590.7	2	55	CTGLSDDCPR	
34	0.01	109.1	540.9	3	63	KSVQPQAQSWSCSK	PDE; *P. elegans*; ~BAP39928
			596.1	4	61	AKRPDFYTLYIEEPDTTGHK	
			746.8	2	35	SVQPQAQSWSCSK	

Peak	%	MW (kDa)	Peptide Ion		Score	MS/MS-derived sequence	Protein family/species/accession
			m/*z*	*z*			
		102.2	1121.6	1	23	NPFYTPSPAK	PDE; *P. flavoviridis*; ~T2HP62
			1355.7	1	10	AATYFWPGSEVK	
			1924.0	1	16	VRDVELLTGLNFYSGLK	
			2360.1	1	34	EQSSPLSCPFGPVPSPDVSGCR	
			2405.2	1	20	TESEAHNLPYGRPQVLQNHSK	
	0.02	67.6	513.2	2	52	QDCYGGVAR	5'NT; *P. elegans*; ~BAP39925
			408.2	3	51	MGKVYPAVEGR	
			445.7	2	51	VYPAVEGR	
			513.2	2	46	NYSSQEIGK	
		41.7	725.4	2	70	VVSLNVLCTECR	5'NT; *P. elegans*; ~BAP39925
			523.9	3	66	HGQGMGELLQVSGIK	
			519.3	3	63	IIALGHSGFLEDQR	
			513.2	2	51	QDCYGGVAR	
			653.9	2	47	QVPVVQAYAFGK	
			686.0	3	45	ASGNPILLNKDISEDQDVK	
			476.3	2	44	VGIIGYTTK	
			430.2	2	41	IINVGSEK	
	0.05	57.9	423.6	3	67	SGTKIFLTCNK	LAAO; *P. elegans*; ~BAP3995
			699.7	3	59	NCADIVMNDLSLIHQLPK	
			557.0	4	58	NCADIVMNDLSLIHQLPKK	
		55.1	784.4	3	104	YPVKPSEEGKSAGQLFEESLR	LAAO; *P. elegans*; ~BAP3995
			618.8	2	66	SAGQLFEESLR	
			634.8	2	65	SGTKIFLTCNK	
			682.9	2	60	SAGQLFEESLRK	
			490.3	2	53	EYLIKEGK	
			694.4	3	46	NCADIVMNDLSLIHQLPK	
			699.7	3	43	NCADIVMNDLSLIHQLPK	
		33.0	618.8	2	63	SAGQLFEESLR	LAAO; *P. elegans*; ~BAP3995
			634.3	3	54	DTSLVTADYVIVCTTSR	
			682.9	2	53	SAGQLFEESLRK	
			582.3	3	48	KDIQAFCYPSMIQK	
		32.3	711.0	3	86	RFDEIVGGMDQLPTSMYR	LAAO; *P. elegans*; ~BAP3995
			618.8	2	63	SAGQLFEESLR	
			951.0	2	61	DTSLVTADYVIVCTTSR	
			577.0	3	55	KDIQAFCYPSMIQK	
			757.9	2	53	ETDYEEFLEIAR	
			569.3	2	51	HDDIFAYEK	
			582.3	3	50	KDIQAFCYPSMIQK	

Peak	%	MW (kDa)	Peptide Ion m/z	Peptide Ion z	Score	MS/MS-derived sequence	Protein family/species/accession
			490.3	2	47	EYLIKEGK	
			659.0	3	45	FDEIVGGMDQLPTSMYR	
	0.09	48.7	1512.7	1	94	VIGGDECNINEHR	SVSP; *Calloselasma rhodostoma*; ~P26324
		31.5	2119.1	1	34	SAHIAPLSLPSSPPSVGSVCR	SVSP; *P. flavoviridis*; ~T2HQA7
		34.3	968.5	1	16	FFCPNRK	SVSP; *T. stejnegeri*; ~Q91516
			1097.5	1	45	EKFFCPNR	
			2506.2	1	115	EIYPDVPHCANINILDHAVCR	
			2879.5	1	185	LDSSVSNSEHIAPLSLPSSPPSVGSVCR	
			3046.6	1	122	TIPTKEIYPDVPHCANINILDHAVCR	
	1.13	38.6	792.3	3	53	GAGTECRAASSECDMADLCTGR	SVMP; *P. mucrosquamatus*; ~XP_02942320
			548.6	3	41	AASSECDMADLCTGR	
		27.8	1129.5	1	51	NDYQTFLTK	SVMP; *P. flavoviridis*; ~P20165
			1966.9	1	100	LFSDCSKNDYQTFLTK	
		25.0	933.5	1	26	DSDKITVR	SVMP; *P. jerdonii*; P0C6E4
			1208.6	1	71	LSVGLVQDYSK	
			1388.7	1	4	LAIVVDYGMYTK	
			2569.3	1	118	NDYQTFLTNYKPQCILNAPLR	
		24.0	609.9	3	60	CDTCIMSAVISDKPSK	SVMP; *P. jerdonii*; PJD|c52836_g4
			604.6	3	53	CDTCIMSAVISDKPSK	
			647.6	4	49	SHDCAHLLTDTKLDDNTIGVAYK	
			668.9	2	40	LDDNTIGVAYKK	
			638.3	3	38	GMCDPKLSVGLVQDYSK	
			686.9	2	36	LAIVVDYGMYTK	
			694.9	2	36	LAIVVDYGMYTK	
			428.7	2	35	LFSDCSK	
			466.6	3	31	SHDCAHLLTDTK	
			608.0	3	28	LAIVVDYGMYTKYNR	
		16.4	1006.4	2	82	LTPGSQCAEGLCCDQCR	SVMP; *P. jerdonii*; P0C6E4
			666.9	3	69	GDYPDDRCTGLSDDCPR	
			466.6	3	63	SHDCAHLLTDTK	
			604.8	2	61	LSVGLVQDYSK	
			590.7	2	58	CTGLSDDCPR	
		11.5	1006.4	2	75	LTPGSQCAEGLCCDQCR	SVMP; *P. jerdonii*; P0C6E4
			699.3	2	74	SHDCAHLLTDTK	
			666.9	3	71	GDYPDDRCTGLSDDCPR	
			625.6	3	70	IHNDADSTASISACDGLK	
			604.8	2	68	LSVGLVQDYSK	
			590.7	2	56	CTGLSDDCPR	

Peak	%	MW (kDa)	Peptide Ion		Score	MS/MS-derived sequence	Protein family/species/accession
			m/*z*	*z*			
35	4.33	91.1	2225.8	1	121	LHSWVECESGECCEOCR	SVMP; *P. flavoviridis*; ~A0A077LAC3
		57.9	1612.8	1	92	IYACVNTLNLIYR	SVMP; *Crotalus adamanteus*; ~J3SDW8
			2402.0	1	76	LRPGTQCEDGECCEQCQFK	
		49.1	1812.7	1	130	QGAQCAEGLCCDQCR	SVMP; *D. acutus*; ~Q1PS45
			2053.9	1	150	LKQGAQCAEGLCCDQCR	
			2303.0	1	27	SCVMAGTLSCEASYLFSDCSR	
		25.2	1208.7	1	8	LSVGLVQDYSK	SVMP; *P. jerdonii*; P0C6E4
			1388.7	1	5	LAIVVDYGMYTK	
		11.3	666.9	3	59	GDYPDDRCTGLSDDCPR	SVMP; *P. jerdonii*; P0C6E4
			1006.4	2	49	LTPGSQCAEGLCCDQCR	
			590.7	2	44	CTGLSDDCPR	
	0.14	43.6	746.7	3	60	HQGLPESYNFDFVTMKPVL	PLB; *P. elegans*; ~BAP39922
			453.2	2	58	VADISMAAK	
			666.9	2	57	VVPESLFAWER	
			469.6	3	55	FTAYAINGPPVEK	
			682.6	3	52	IANMMADSGKTWAETFEK	
			487.6	3	47	KVVPESLFAWER	
			741.4	3	44	HQGLPESYNFDFVTMKPVL	
			684.8	2	44	DQGKVTDMESMK	
			468.9	3	44	YNNYKEDPYAK	
			470.7	2	41	VTDMESMK	
			481.6	3	41	HLDFKITDPQTK	
			401.7	2	40	ITDPQTK	
			682.6	3	38	IANMMADSGKTWAETFEK	
		34.5	666.9	2	63	VVPESLFAWER	PLB; *P. elegans*; ~BAP39922
			453.2	2	59	VADISMAAK	
			450.9	3	56	RTPVPAGCYDSK	
			703.9	2	46	FTAYAINGPPVEK	
			555.3	2	46	YSDQTNILR	
	0.04	40.2	448.2	3	68	HLVIACHYDSK	QC; *P. flavoviridis*; ~BAN89376
			754.8	3	65	NTYQIQGIDLFVLLDLIGAR	
			591.0	3	62	NLYDLGLLNNYSSER	
			500.9	3	61	YPVEDDHIPFLR	
			561.0	3	60	MWQNDLHPILIER	
			553.0	3	54	RYPVEDDHIPFLR	
			515.0	3	53	GVPILHLIPSPFPR	
			513.3	3	53	NPVFPVYFLNTAR	
			713.4	3	52	VFVGATDSAVPCAMMLELAR	

Peak	%	MW (kDa)	Peptide Ion m/z	z	Score	MS/MS-derived sequence	Protein family/species/accession
			532.8	2	51	YFPPQLDGK	
			627.8	2	47	WSPSDSLYGSR	
			481.6	3	47	LIFFDGEEAFVR	
	0.23	16.4	705.9	2	62	TCLGLEKETGFR	CTL; *P. flavoviridis*; ~Q7LZ7
			552.0	4	55	DCPSGWSSYEGHCYKPFK	
			432.9	3	47	LYKTWDDAER	
36	3.69	63.4	1025.5	1	51	FKGAGTECR	SVMP; *Phalotris mertensi*; ~A0A82C5Q9
			1830.0	1	34	KYIEFVIVVDNGMFR	
		50.1	694.6	3	77	LRQGAQCAEGLCCDQCR	SVMP; *P. flavoviridis*; ~Q90ZI3
			843.3	2	50	AATDECDMADLCTGR	
			851.3	2	42	AATDECDMADLCTGR	
		47.8	694.6	3	59	LRQGAQCAEGLCCDQCR	SVMP; *P. flavoviridis*; ~Q90ZI3
		25.8	933.5	1	67	DSDKITVR	SVMP; *P. jerdonii*; P0C6E4
			1208.6	1	72	LSVGLVQDYSK	
			1397.6	1	56	SHDCAHLLTDTK	
	0.09	56.1	711.0	3	71	RFDEIVGGMDQLPTSMYR	LAAO; *P. elegans*; ~BAP3995
			618.8	2	68	SAGQLFEESLR	
			711.0	3	60	RFDEIVGGMDQLPTSMYR	
			705.7	3	58	RFDEIVGGMDQLPTSMYR	
			682.9	2	53	SAGQLFEESLRK	
			490.3	2	48	EYLIKEGK	
			588.6	4	46	YPVKPSEEGKSAGQLFEESLR	
			716.3	3	45	RFDEIVGGMDQLPTSMYR	
	0.06	34.5	749.8	2	60	VVGGDECNINEHR	SVSP; *Agkistrodon contortrix contortrix*; ~P8298
	0.41	16.4	993.5	1	48	SYVWIGLR	CTL; *P. flavoviridis*; ~Q7LZ7
			1410.7	1	86	TCLGLEKETGFR	
		11.0	905.4	1	15	STNNKWR	CTL; *P. flavoviridis*; ~T2HP57
37	0.31	110.5	-	-	-	-	Unknown
		63.4	-	-	-	-	Unknown
	0.90	73.1	742.6	3	66	LHSWVECESGECCEQCR	SVMP; *P. mucrosquamatus*; ~XP_05683679
		51.5	843.3	2	57	AATDECDMADLCTGR	SVMP; *P. flavoviridis*; ~Q90ZI3
			567.9	3	42	AATDECDMADLCTGR	
		26.3	602.6	3	58	LAIVVDYGMYTKYNR	SVMP; *P. jerdonii*; P0C6E4
			466.6	3	55	SHDCAHLLTDTK	
			604.8	2	46	LSVGLVQDYSK	
			608.0	3	44	LAIVVDYGMYTKYNR	
		11.0	1006.4	2	57	LTPGSQCAEGLCCDQCR	SVMP; *P. jerdonii*; P0C6E4
			604.8	2	51	LSVGLVQDYSK	

Peak	%	MW (kDa)	Peptide Ion		Score	MS/MS-derived sequence	Protein family/species/accession
			m/z	z			
38	0.21	36.0	2879.4	1	86	LDSSVSNSEHIAPLSLPSSPPSVGSVCR	SVSP; *T. stejnegeri*; ~Q91516
			3046.5	1	26	TIPTKEIYPDVPHCANINILDHAVCR	
	0.35	16.7	993.6	1	35	SYVWIGLR	CTL; *P. flavoviridis*; ~Q7LZ7
			2204.9	1	66	DCPSGWSSYEGHCYKPFK	
	1.91	49.1	1174.6	1	38	IFPCAPQDVK	SVMP; *P. flavoviridis*; ~G1UJB2
			1472.8	1	84	YLISHTPQCILK	
			1968.0	1	114	YLISHTPQCILKEPLR	
		26.3	933.5	1	55	DSDKITVR	SVMP; *P. jerdonii*; P0C6E4
			1208.6	1	70	LSVGLVQDYSK	
	0.02	35.2	968.5	1	18	FFCPNRK	SVSP; *T. stejnegeri*; ~Q91516
			1097.5	1	26	EKFFCPNR	
			2879.4	1	96	LDSSVSNSEHIAPLSLPSSPPSVGSVCR	
	0.51	16.7	993.5	1	51	SYVWIGLR	CTL; *P. flavoviridis*; ~Q7LZ7
			1410.7	1	88	TCLGLEKETGFR	
			2204.9	1	92	DCPSGWSSYEGHCYKPFK	
		11.3	1843.8	1	47	YKAWAEESYCVYFK	CTL; *P. flavoviridis*; ~A0A077LD73

Methionine oxidation is underlined.

Supplementary table 3 Assignment of the reverse-phase chromatographic fractions from *Protobothrops mangshanensis* venom to protein families by nESI-MS/MS of selected peptide ions from in-gel digested protein bands separated by SDS-PAGE.

Peak	%	MW (kDa)	Peptide Ion		Score	MS/MS-derived sequence	Protein family/species/accession
			m/*z*	*z*			
1	0.88	-	679.4	2	42	PPISPLSVQHWP	BPP/CNP; *Protobothrops jerdonii*; ~PJD|c31701
2	1.82	-	622.3	2	71	QSKPGRPPPISP	BPP/CNP; *P. jerdonii*; ~PJD|c31701
3	0.56	-	719.8	2	107	QPHESPAGGTTAFR	BPP/CNP; *P. jerdonii*; ~PJD|c31701
			622.3	2	101	QSKPGRPPPISP	
			530.3	2	43	QSKPGRPPPI	
4	0.42	88.3	587.8	2	31	MIPCAPQDVK	SVMP; *Protobothrops elegans*; ~BAP39935
		38.7	502.7	2	51	GNDYGYCR	SVMP; *P. elegans*; ~BAP39935
			587.8	2	45	MIPCAPQDVK	
			579.8	2	38	MIPCAPQDVK	
	0.04	80.5	-	-	-	-	Unknown
		74.7	-	-	-	-	Unknown
5	0.06	27.8	906.8	2	79	QGAQCAEGLCCDQCR	Disintegrin; *Protobothrops flavoviridis*; ~Q90ZI3
			529.8	2	43	IACEPQNVK	
6	0.17	5.8	-	-	-	-	Unknown
7	0.09	3.4	-	-	-	-	Unknown
8	0.11	-	-	-	-	-	Unknown
9	0.08	17.7	682.3	2	68	ALTMEGNQASWR	NGF; *P. flavoviridis*; ~BAN82015
			647.3	2	63	IDTACVCVISR	
			855.4	2	53	FIRIDTACVCVISR	
			556.3	2	44	NPNPVPTGCR	
10	0.06	18.5	570.6	3	61	FIRIDTACVCVISR	NGF; *P. flavoviridis*; ~BAN82015
			647.3	2	59	IDTACVCVISR	
			556.3	2	40	NPNPVPTGCR	
	0.39	14.9	633.9	5	47	ETLVPILKEYPDEVSHLFKPSCVPVLR	VEGF; *P. flavoviridis*; ~P67862
11	0.77	16.5	622.3	2	46	NLGTYDKNYR	PLA_2; *Protobothrops mangshanensis*; P84776
			449.9	3	40	DRYSYSWENK	
			579.6	3	37	ELCECDKAVAICLR	
			401.7	2	34	AVAICLR	
			463.6	3	31	AIVCGEKNPCLK	
			470.2	2	30	MVFQETGK	
12	8.24	49.3	583.3	2	41	NVDFDSESPR	CRISP; *P. flavoviridis*; ~1WVR_A
			636.3	2	36	CPASCFCQNK	
		27.4	743.8	2	51	SKCPASCFCQNK	CRISP; *P. flavoviridis*; ~6IMF_A
			636.3	2	49	CPASCFCQNK	
			499.9	3	38	CPASCFCQNK	

Peak	%	MW (kDa)	Peptide Ion		Score	MS/MS-derived sequence	Protein family/species/accession
			m/*z*	*z*			
			512.3	4	38	WTDIIHAWHGEYKDFK	
		22.3	777.3	2	94	MEWYPEAAANAER	CRISP; *P. flavoviridis*; ~Q8JI39
			581.3	2	63	SVNPTASNMLK	
			904.8	3	52	SVNPTASNMLKMEWYPEAAANAER	
		20.0	583.3	2	56	NVDFDSESPR	CRISP; *P. flavoviridis*; ~6IMF_A
	0.49	23.8	-	-	-	-	Unknown
	0.46	17.4	581.6	3	71	EICECDKAAAICFR	PLA$_2$; *P. mangshanensis*; AHJ09547
			704.3	2	65	DIVCGEDNPCTK	
			730.7	3	52	VTGCNPKDDFYIYSLQNR	
			717.3	2	35	DDFYIYSLQNR	
		14.7	717.3	2	63	DDFYIYSLQNR	PLA$_2$; *P. mangshanensis*; AHJ09547
13	2.27	43.1	716.0	3	93	SAHIAPLSLPSNPPSVGSVCR	SVSP; *Agkistrodon piscivorus conanti*; ~JAS04401
			569.3	4	62	KSAHIAPLSLPSNPPSVGSVCR	
		40.2	594.8	2	26	WNKDIMLIR	SVSP; *P. flavoviridis*; ~O13057
		37.2	536.9	3	49	TLCAGILEGGKDSCK	SVSP; *P. flavoviridis*; ~O13057
		31.0	763.9	2	62	IIGGDECNINEHR	SVSP; *Gloydius halys*; ~B0ZT25
		23.6	583.8	2	52	VMGWGTISATK	SVSP; *P. jerdonii*; ~ABZ04157
		18.5	565.3	2	38	ILCAGILQGGK	SVSP; *Protobothrops mucrosquamatus*; ~XP_015671562
	0.08	20.2	647.3	2	56	IDTACVCVISR	NGF; *P. flavoviridis*; ~BAN82015
14	0.20	46.8	565.3	2	50	ILCAGILQGGK	SVSP; *P. flavoviridis*; ~O13058
		40.5	756.8	2	45	VIGGDECNINEHR	SVSP; *Gloydius blomhoffii;* ~CAB65936
		37.0	559.8	2	63	TLCAGILEGGK	SVSP; *P. jerdonii*; ~B0ZT25
			575.8	2	45	VMGWGTISATK	
		29.4	565.3	2	39	ILCAGILQGGK	SVSP; *P flavoviridis*; ~O13058
15	1.20	47.8	411.2	3	49	KVLNEDEQTR	SVSP; *P. jerdonii*; ~AAG10790
			552.3	2	46	VLNEDEQTR	
		40.5	550.8	2	45	NVPNEDEQR	SVSP; *Trimeresurus stejnegeri*; ~Q8AY80
			419.5	3	44	NVPNEDEQRR	
		33.2	473.3	2	37	EWVLTAAR	SVSP; *P. mucrosquamatus*; ~XP_015671555
		31.4	473.3	2	41	EWVLTAAR	SVSP; *P. mucrosquamatus*; ~XP_015671555
		16.3	411.2	3	55	KVLNEDEQTR	SVSP; *P. jerdonii*; ~Q9DF66
			552.3	2	48	VLNEDEQTR	
		14.7	411.2	3	46	KVLNEDEQTR	SVSP; *P. jerdonii*; ~Q9DF66
			552.3	2	44	VLNEDEQTR	
16	2.48	48.8	504.9	3	41	VIGGDECNINEHR	SVSP; *Agkistrodon contortrix contortrix*; ~2AIQ_A
		43.4	763.9	2	59	IIGGDECNINEHR	SVSP; *G. halys*; ~4E7N_A
		33.5	444.9	3	47	SKTLCAGILQGGK	SVSP; *P. jerdonii*; ~Q9DF66
		30.9	582.8	2	31	IMGWGTISATK	SVSP; *P. flavoviridis*; ~BAP39955

Peak	%	MW (kDa)	Peptide Ion		Score	MS/MS-derived sequence	Protein family/species/accession
			m/z	z			
		27.8	559.3	2	41	TLCAGILQGGK	SVSP; *P. jerdonii*; ~AAG10790
			552.3	2	33	VLNEDEQTR	
		20.3	559.3	2	52	TLCAGILQGGK	SVSP; *P. jerdonii*; ~AAG10790
			552.3	2	48	VLNEDEQTR	
			411.2	3	42	KVLNEDEQTR	
		19.2	552.3	2	47	VLNEDEQTR	SVSP; *P. jerdonii*; ~AAG10790
			411.2	3	44	KVLNEDEQTR	
			559.3	2	42	TLCAGILQGGK	
		16.5	552.3	2	50	VLNEDEQTR	SVSP; *P. jerdonii*; ~Q9DF66
			411.2	3	47	KVLNEDEQTR	
		15.0	411.2	3	54	KVLNEDEQTR	SVSP; *P. jerdonii*; ~Q9DF66
			552.3	2	49	VLNEDEQTR	
17	1.30	50.3	504.9	3	47	VIGGDECNINEHR	SVSP; *Gloydius blomhoffii*; ~CAB65936
		45.4	590.8	2	49	IMGWGTISATK	SVSP; *P. flavoviridis*; ~BAP39955
		33.5	588.3	2	50	AAYPELPATSR	SVSP; *P. mucrosquamatus*; ~XP_015671560
			448.8	2	40	VTLPDVPR	
			820.4	2	35	CANINLLDYEVCR	
	3.28	17.4	528.3	4	60	FCSEQVKGAHLVSIESYR	CTL; *P. mucrosquamatus*; ~XP_015685588
		14.8	914.9	2	47	TTDNQWLSMDCSSKR	CTL; *P. mucrosquamatus*; ~Q5FZI5
			922.9	2	38	TTDNQWLS<u>M</u>DCSSKR	
18	4.24	107.4	442.2	3	58	CIELVIVADHR	SVMP; *Ovophis okinavensis*; ~BAN82161
		44.1	1005.4	2	69	GDWNDDRCTGQSADCPR	SVMP; *P. mucrosquamatus*; ~E9NW26
			576.2	2	58	CTGQSADCPR	
	0.98	85.1	-	-	-	-	Unknown
		18.2	-	-	-	-	Unknown
	2.74	56.8	754.1	3	50	NSAHIAPLSLPSNPPSVGSVCR	SVSP; *Gloydius brevicaudus*; ~AAF25008
		49.3	559.8	2	55	TLCAGILEGGK	SVSP; *G. blomhoffii*; ~CAB65936
			504.9	3	47	VIGGDECNINEHR	
		35.2	570.8	2	55	AAKPELPATSR	SVSP; *Crotalus adamanteus*; ~J3SDW9
		33.5	588.3	2	42	AAYPELPATSR	SVSP; *P. mucrosquamatus*; ~Q91508
		29.5	559.8	2	49	TLCAGILEGGK	SVSP; *Protobothrops elegans*; ~P84787
		25.2	559.8	2	58	TLCAGILEGGK	SVSP; *P. mucrosquamatus*; ~XP_015671557
		23.5	559.8	2	56	TLCAGILEGGK	SVSP; *P. mucrosquamatus*; ~XP_015671557
		22.4	559.8	2	73	TLCAGILEGGK	SVSP; *P. mucrosquamatus*; ~XP_015671557
	0.98	16.1	808.4	2	42	YNAWSAESECIASK	CTL; *P. jerdonii*; ~D1MGU1
			588.8	2	40	LQWSDGTELK	
19	0.71	56.8	754.1	3	48	NSAHIAPLSLPSNPPSVGSVCR	SVSP; *Agkistrodon piscivorus leucostoma*; ~ADP88562

Peak	%	MW (kDa)	Peptide Ion		Score	MS/MS-derived sequence	Protein family/species/accession
			m/*z*	*z*			
		49.3	562.3	2	48	SVQFDDEQR	SVSP; *Trimeresurus stejnegeri*; ~AAN52347
		43.7	562.3	2	27	SVQFDDEQR	SVSP; *Ovophis okinavensis*; ~BAN82129
	0.48	18.2	-	-	-	-	Unknown
	0.40	16.0	797.9	2	46	LASIQSSEEEAFVGK	CTL; *P. flavoviridis*; ~BAP39991
20	0.12	44.3	626.8	2	55	SAGQLYEESLR	LAAO; *P. flavoviridis*; ~BAN82013
			438.7	2	47	STTDLPSR	
		33.5	626.8	2	44	SAGQLYEESLR	LAAO; *P. flavoviridis*; ~BAN82013
			412.6	3	36	KVTVTYQTPAK	
			438.7	2	35	STTDLPSR	
		31.6	626.8	2	46	SAGQLYEESLR	LAAO; *P. flavoviridis*; ~BAN82013
			438.7	2	38	STTDLPSR	
	1.82	18.5	411.2	3	51	GAHLVSIESYR	CTL; *Trimeresurus purpureomaculatus*; ~P0DJL2
			481.9	3	31	YHVWIGLSVQNK	
		15.6	420.7	2	31	TVSFVCK	CTL; *P. jerdonii*; ~PJD|c42983
			797.9	2	28	LASIQSSEEEAFVGK	
			532.7	2	24	WEWSDDAR	
21	2.36	18.2	616.3	2	66	GAHLVSIESYR	CTL; *P. mucrosquamatus*; ~XP_015685588
			449.2	2	18	FCSEQVK	
		15.7	584.3	2	37	EQFECLVSR	CTL; *Trimeresurus albolabris*; ~P81114
			959.9	2	34	DCPSDWSSYEGHCYR	
22	5.38	17.4	411.2	3	66	GAHLVSIESYR	CTL; *Protobothrops elegans*; ~BAP39929
		15.5	616.3	2	52	GAHLVSIESYR	CTL; *P. elegans*; ~BAP39929
23	0.72	103.3	-	-	-	-	Unknown
	0.16	66.2	782.4	2	65	CSSITELEKVNQR	PDE; *P. elegans*; ~BAP39928
			561.3	2	58	NPFYTPSPAK	
			787.9	2	51	STPTSVPPSASDCLR	
		57.4	782.4	2	68	CSSITELEKVNQR	PDE; *P. flavoviridis*; ~BAN82021
			787.9	2	59	STPTSVPPSASDCLR	
			561.3	2	49	NPFYTPSPAK	
			530.8	2	41	SVQPQVSCR	
		43.8	782.4	2	53	CSSITELEKVNQR	PDE; *P. flavoviridis*; ~BAN82021
			787.9	2	49	STPTSVPPSASDCLR	
			403.2	2	41	ALEMADR	
			457.8	2	39	LNLNNQAK	
			419.8	2	38	IQIHTAR	
	4.50	17.1	483.8	2	37	LVSIESYR	CTL; *P. jerdonii*; ~D1MGU0
		15.0	959.9	2	80	DCPSDWSSYEGHCYR	CTL; *T. albolabris*; ~P81114
			774.4	2	56	VFNEPQNWADAEK	

Peak	%	MW (kDa)	Peptide Ion m/*z*	*z*	Score	MS/MS-derived sequence	Protein family/species/accession
			826.4	3	53	VFNEPQNWADAEKFCTQQHK	
24	0.12	103.3	787.9	2	35	STPTSVPPSASDCLR	PDE; *P. mucrosquamatus*; ~PMR|c20258
			561.3	2	33	NPFYTPSPAK	
			457.8	2	25	LNLNNQAK	
	4.27	16.9	411.2	3	70	GAHLVSIESYR	CTL; *P. elegans*; ~BAP39929
			501.6	3	42	QYCYKPFKQLK	
			567.3	2	35	QYCYKPFK	
		15.0	826.4	3	69	VFNEPQNWADAEKFCTQQHK	CTL; *T. albolabris*; ~P81114
			640.2	3	69	DCPSDWSSYEGHCYR	
			584.3	2	52	EQFECLVSR	
25	0.04	99.4	746.8	2	51	SVQPQAQSWSCSK	PDE; *P. elegans*; ~BAP39928
			532.3	2	47	GETSWLKDK	
			410.2	2	38	SIPFEAR	
			403.2	2	36	ALEMADR	
	0.33	58.6	811.0	3	34	AAGTECRAATDECDMADLCTGR	SVMP; *P. flavoviridis*; ~Q90ZI3
			656.4	2	34	IIVQSVPDVTLK	
			423.9	3	41	SAECTDRFQR	
			502.3	2	37	NMPQCILK	
		44.1	906.8	2	70	QGAQCAEGLCCDQCR	SVMP; *P. flavoviridis*; ~Q90ZI3
			694.6	3	62	LRQGAQCAEGLCCDQCR	
			477.9	3	45	IACEPQNVKCGR	
			409.7	2	39	GMVLPGTK	
			502.3	2	39	NMPQCILK	
	3.84	16.9	640.2	3	57	DCPSDWSSYEGHCYR	CTL; *T. albolabris*; ~P81114
			620.0	4	41	VFNEPQNWADAEKFCTQQHK	
			584.3	2	40	EQFECLVSR	
			774.4	2	37	VFNEPQNWADAEK	
		15.2	959.9	2	83	DCPSDWSSYEGHCYR	CTL; *T. albolabris*; ~P81114
			1239.1	2	66	VFNEPQNWADAEKFCTQQHK	
			774.4	2	53	VFNEPQNWADAEK	
26	0.03	103.3	561.3	2	54	NPFYTPSPAK	PDE; *P. flavoviridis*; ~BAN82023
			782.4	2	46	CSSITELEKVNQR	
	0.12	58.6	694.6	3	74	LRQGAQCAEGLCCDQCR	SVMP; *P. flavoviridis*; ~Q90ZI3
			906.8	2	58	QGAQCAEGLCCDQCR	
			502.3	2	39	NMPQCILK	
			423.9	3	36	SAECTDRFQR	
			656.4	2	31	IIVQSVPDVTLK	
	0.10	43.7	666.9	2	69	VVPESLFAWER	PLB; *P. flavoviridis*; ~BAN82026

Peak	%	MW (kDa)	Peptide Ion m/*z*	*z*	Score	MS/MS-derived sequence	Protein family/species/accession
			479.6	3	51	HGLEFSYEMAPR	
			453.2	2	40	VADISMAAK	
			597.8	2	37	TPVPAGCYDSK	
	0.05	31.7	438.7	2	54	STTDLPSR	LAAO; *P. flavoviridis*; ~BAP39950
			626.8	2	49	SAGQLYEESLR	
		29.9	626.8	2	73	SAGQLYEESLR	LAAO; *P. flavoviridis*; ~BAN82014
	2.01	16..5	411.2	3	76	GAHLVSIESYR	CTL; *P. elegans*; ~BAP39929
			567.3	2	39	QYCYKPFK	
		14.7	914.9	2	49	TTDNQWLSMDCSSKR	CTL; *P. mucrosquamatus*; ~Q5FZI5
			922.9	2	31	TTDNQWLSMDCSSKR	
27	3.86	67.5	906.8	2	81	QGAQCAEGLCCDQCR	SVMP; *T. stejnegeri*; ~ABC73079
			694.6	3	67	LRQGAQCAEGLCCDQCR	
			801.0	3	55	GAGTECRPASSECDMADLCTGR	
			502.3	2	47	NMPQCILK	
			423.9	3	47	SAECTDRFQR	
		59.2	477.9	3	47	IACEPQNVKCGR	SVMP; *P. flavoviridis*; ~Q90ZI3
			529.8	2	43	IACEPQNVK	
		40.8	477.9	3	60	IACEPQNVKCGR	SVMP; *P. flavoviridis*; ~Q90ZI3
	0.32	44.4	468.9	3	56	YNNYKEDPYAK	PLB; *P. flavoviridis*; ~BAN82026
			597.8	2	42	TPVPAGCYDSK	
			453.2	2	38	VADISMAAK	
			450.9	3	33	RTPVPAGCYDSK	
	1.92	16.5	-	-	-	-	Unknown
	2.78	14.7	584.3	2	52	EQFECLVSR	CTL; *T. albolabris*; ~P81114
			640.2	3	60	DCPSDWSSYEGHCYR	
28	7.98	90.2	423.9	3	31	SAECTDRFQR	SVMP; *P. jerdonii*; ~PJD|c52836
			579.8	2	30	MIPCAPQDVK	
			587.8	2	30	MIPCAPQDVK	
		80.3	423.9	3	46	SAECTDRFQR	SVMP; *P. flavoviridis*; ~BAN82007
		59.2	906.8	2	79	QGAQCAEGLCCDQCR	SVMP; *P. flavoviridis*; ~Q90ZI3
			477.9	3	76	IACEPQNVKCGR	
			423.9	3	44	SAECTDRFQR	
		16.7	562.8	2	37	QCVDVNRAY	SVMP; *Crotalus horridus*; ~JAG46111
	4.75	14.8	-	-	-	-	Unknown
29	0.94	72.9	634.8	2	36	DESIGMVDTGTK	SVMP; *P. mucrosquamatus*; ~XP_015683679
	6.17	17.6	-	-	-	-	Unknown
		15.9	-	-	-	-	Unknown
30	0.43	77.2	626.8	2	40	DESIGMVDTGTK	SVMP; *P. mucrosquamatus*; ~XP_015683679

Peak	%	MW (kDa)	Peptide Ion		Score	MS/MS-derived sequence	Protein family/species/accession
			m/*z*	*z*			
			514.3	2	39	IPCAPQDVK	
			634.8	2	36	DESIGMVDTGTK	
	1.72	17.4	410.7	2	29	TCLGLEK	CTL; *P. flavoviridis*; ~BAN81993
		15.6	584.3	2	55	EQFECLVSR	CTL; *T. albolabris*; ~3023232
31	1.98	50.3	700.3	3	68	LRPGAQCAEGLCCYQCR	SVMP; *Sistrurus tergeminus*; ~JAS05270
			587.8	2	33	IFPCAPQDVK	
	3.07	16.8	584.3	2	40	EQFECLVSR	CTL; *T. albolabris*; ~P81114
		16.6	584.3	2	38	EQFECLVSR	CTL; *T. albolabris*; ~P81114
32	1.28	49.8	502.9	3	55	VCSNRQCVDVNR	SVMP; *Sistrurus tergeminus*; ~JAS05269
	0.72	16.7	410.7	2	31	TCLGLEK	CTL; *P. flavoviridis*; ~BAN81993
		15.0	410.7	2	25	TCLGLEK	CTL; *P. jerdonii*; ~PJD|c52836
33	0.04	38.3	453.7	2	45	YHFVANR	SVMP; *P. mucrosquamatus*; ~XP_015681821
			505.8	2	41	SVGIVQDHR	
			627.3	2	37	SVGIVQDHRSR	
	0.10	18.5	-	-	-	-	Unknown
		15.1	-	-	-	-	Unknown
	0.01	17.0	410.7	2	31	TCLGLEK	CTL; *P. jerdonii*; ~PJD|c52836

Methionine oxidation is underlined.

Supplementary table 4 Assignment of the reverse-phase chromatographic fractions from *Protobothrops mucrosquamatus* venom to protein families by nESI-MS/MS of selected peptide ions from in-gel digested protein bands separated by SDS-PAGE.

**Peak**	**%**	**MW (kDa)**	**Peptide Ion**		**Score**	**MS/MS-derived sequence**	**Protein family/species/accession**
			**m/*z***	***z***			
1	2.06	-	458.8	4	61	RLPKSKGASATSAASRPM	BPP/CNP; *Protobothrops flavoviridis*; ~P0C7P5
			657.0	3	60	HRLPKSKGASATSAASRPM	
			485.9	3	32	QPHESPAGGTTAFR	
			615.8	2	29	HESPAGGTTAFR	
2	2.67	-	523.3	2	46	KPGRPPPISP	BPP/CNP; *Protobothrops mucrosquamatus*; XP_015680879
			485.9	3	40	QPHESPAGGTTAFR	
			459.3	2	38	PGRPPPISP	
			466.6	3	33	HQSKPGRPPPISP	
			566.8	2	29	SKPGRPPPISP	
3	1.29	-	699.4	2	54	HQSKPGRPPPISP	BPP/CNP; *P. mucrosquamatus*; XP_015680879
			630.9	2	46	QSKPGRPPPISP	
			523.3	2	43	KPGRPPPISP	
4	1.23	-	699.4	2	51	HQSKPGRPPPISP	BPP/CNP; *P. mucrosquamatus*; XP_015680879
5	0.83	-	547.3	3	50	SGNVNDYEVVYPRK	Disintegrin; *P. mucrosquamatus*; E9NW28
			520.8	2	37	DYEVVYPR	
6	0.38	13.8	1257.5	2	124	GDWNDDRCTGQSADCPRNGLYG	Disintegrin; *P. mucrosquamatus*; E9NW26
			784.0	3	112	IARGDWNDDRCTGQSADCPR	
7	0.19	4.1	616.8	2	70	GSGGGGGGLGSNSLR	BPP/CNP; *Bothrops jararaca*; ~Q6LEM5
8	0.14	4.0	883.4	2	77	FSSCGGGGGSFGAGGGFGSR	BPP/CNP; *Gloydius blomhoffii*; ~P01021
9	0.05	3.4	709.9	2	30	GGTTAFREELSPGP	BPP/CNP; *P. flavoviridis*; ~P0C7P5
			454.7	2	28	VHQSKPGR	
10	0.17	4.2	851.4	1	52	IGSTSGLGC	BPP/CNP; *P. mucrosquamatus*; XP_015680879
11	0.06	-	801.3	3	82	LRPGTQCEDGECCEQCQFK	SVMP; *P. mucrosquamatus*; XP_015682631
			1467.6	2	82	AAESECDIAESCTGQSAECPTDDFQR	
			559.8	2	56	LFCVQGPIGK	
			936.9	2	48	NGQPCLNNNGYCYNGK	
12	0.85	14.4	743.4	2	61	AIVCGEKNPPCLK	PLA_2; *P. mucrosquamatus*; Q2PWA3
			861.9	2	60	AVAICLRENLGTYDK	
			881.4	3	54	QVCECDKAVAICLRENLGTYDK	
			848.4	3	54	YSYSWENKAIVCGEKNPPCLK	
			680.9	2	52	ENLGTYDKKHR	
13	15.19	26.6	880.4	2	79	QICECDKAAAMCFR	PLA_2; *P. mucrosquamatus*; Q3HLQ4
			641.8	2	69	AAAMCFRDNVK	
			722.8	4	65	WDQYIYSWENGNIVCGEKNPCKK	
			739.3	3	56	QICECDKAAAMCFRDNVK	

Peak	%	MW (kDa)	Peptide Ion		Score	MS/MS-derived sequence	Protein family/species/accession
			m/z	z			
			863.1	4	55	KMTGKEPILSYATYGCNCGMAGVGQPVDGTDR	
		17.3	1145.2	3	109	KMTGKEPILSYATYGCNCGMAGVGQPVDGTDR	PLA_2; *P. mucrosquamatus*; Q3HLQ4
			1150.5	3	108	KMTGKEPILSYATYGCNCGMAGVGQPVDGTDR	
			1150.5	3	107	KMTGKEPILSYATYGCNCGMAGVGQPVDGTDR	
			867.1	4	106	KMTGKEPILSYATYGCNCGMAGVGQPVDGTDR	
			1107.8	3	105	MTGKEPILSYATYGCNCGMAGVGQPVDGTDR	
			944.4	2	102	KQICECDKAAAMCFR	
			827.1	4	96	MTGKEPILSYATYGCNCGMAGVGQPVDGTDR	
			722.8	4	90	WDQYIYSWENGNIVCGEKNPCKK	
			880.4	2	88	QICECDKAAAMCFR	
			641.8	2	86	AAAMCFRDNVK	
			1108.5	2	79	QICECDKAAAMCFRDNVK	
			837.9	2	73	AAAMCFRDNVKTYK	
			1107.8	3	71	MTGKEPILSYATYGCNCGMAGVGQPVDGTDR	
			413.7	2	53	AAAMCFR	
	0.89	14.2	673.9	3	83	CGGCCSDESLTCTATGKR	VEGF; *P. mucrosquamatus*; Q330K6
			439.0	5	61	IEVMQFKEHTACECRPR	
		12.7	757.7	3	55	EYPNEVSHLFKPSCVPVLR	VEGF; *P. mucrosquamatus*; XP_015676137
			471.8	2	43	EMLVPILK	
14	0.33	16.5	787.0	3	45	KMTGKEPIVSYAFYGCYCGK	PLA_2; *P. flavoviridis*; ~Q8JIG0
			1116.0	2	42	MTGKEPIVSYAFYGCYCGK	
			1124.0	2	41	MTGKEPIVSYAFYGCYCGK	
15	0.71	37.2	764.0	3	49	YRYFYVCQYCPAGNMIGK	CRISP; *P. mucrosquamatus*; P79845
			657.6	3	43	YFYVCQYCPAGNMIGK	
			516.6	3	41	GGCAAAYCPSSKYR	
		27.5	765.3	2	63	CGENIYMSPYPAK	CRISP; *P. mucrosquamatus*; P79845
			652.0	3	59	SYRGGCAAAYCPSSKYR	
			614.8	2	57	GGCAAAYCPSSK	
			750.8	2	56	TKCPASCFCQNK	
			773.3	2	56	CGENIYMSPYPAK	
			985.9	2	50	YFYVCQYCPAGNMIGK	
			663.0	3	40	YFYVCQYCPAGNMIGK	
			705.0	3	35	VIGGIKCGENIYMSPYPAK	
			774.3	2	34	GGCAAAYCPSSKYR	
		23.0	614.8	2	36	GGCAAAYCPSSK	CRISP; *P. mucrosquamatus*; P79845
			774.3	2	35	GGCAAAYCPSSKYR	
		20.4	545.6	3	56	SYRGGCAAAYCPSSK	CRISP; *P. mucrosquamatus*; P79845
