# Supplementary figures and images for: Phylogeny-Related Variations in Venomics: A Test in a Subset of Habu Snakes (Protobothrops)
Source: Toxins (Basel). 2023 May 21;15(5):350. doi: 10.3390/toxins15050350 (PMC10223207; doi:10.3390/toxins15050350)

Figure S1

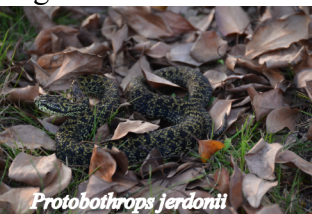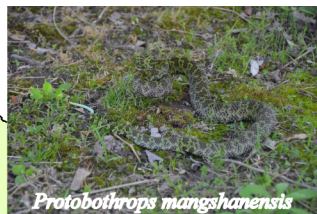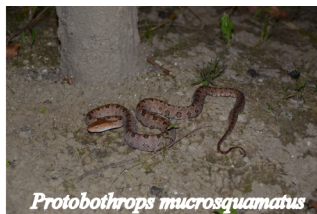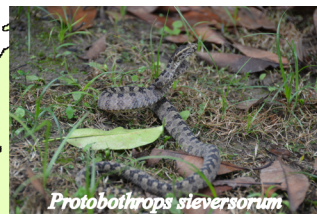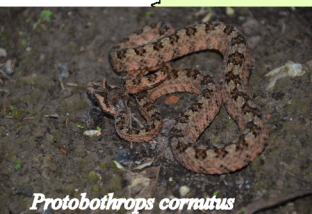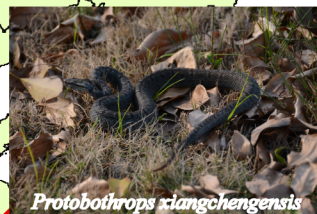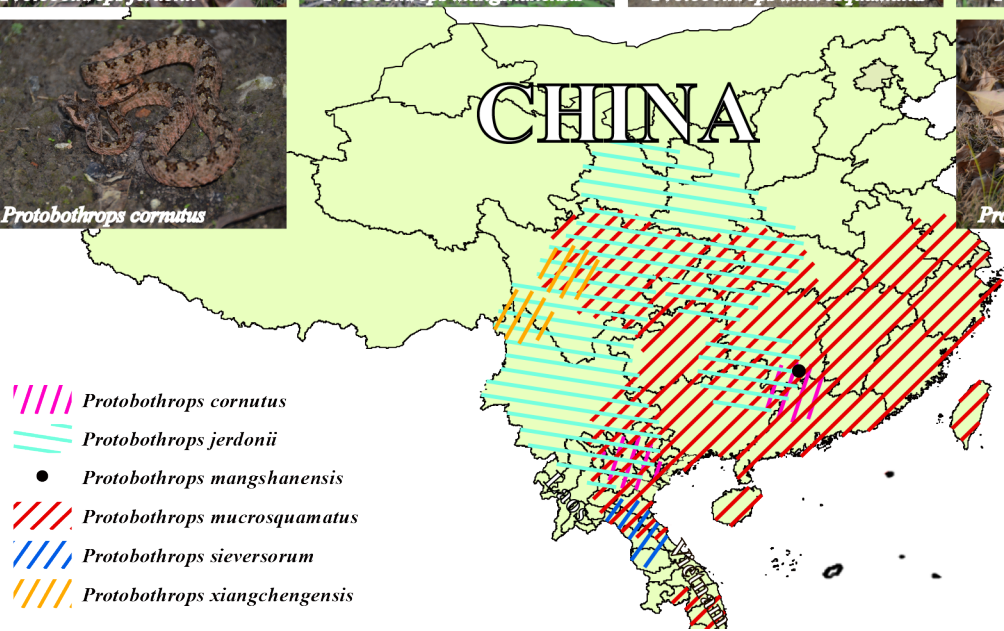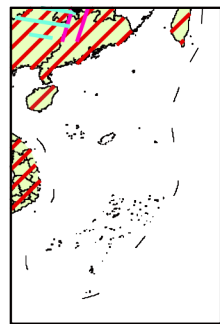

Figure S2

**A**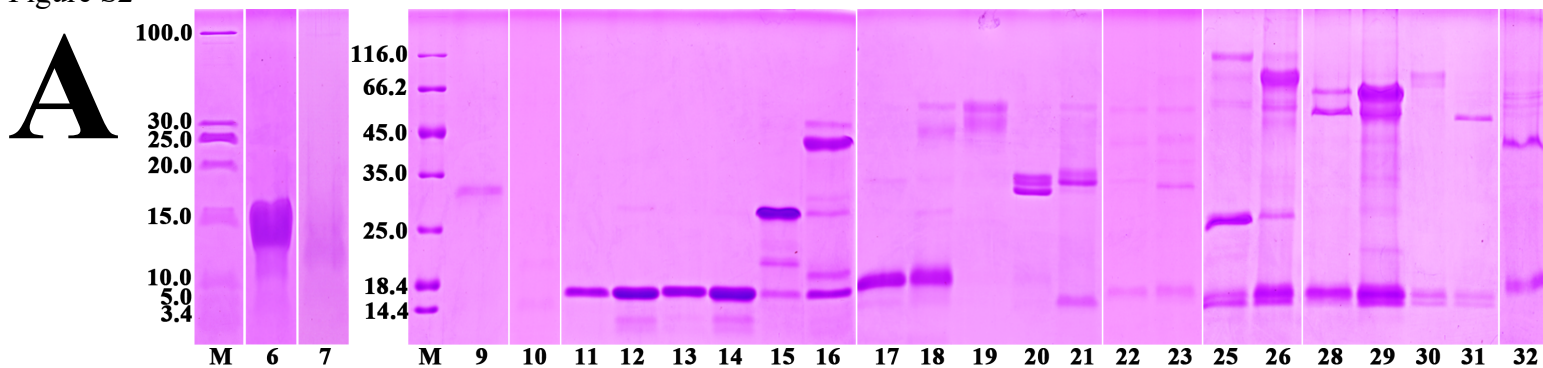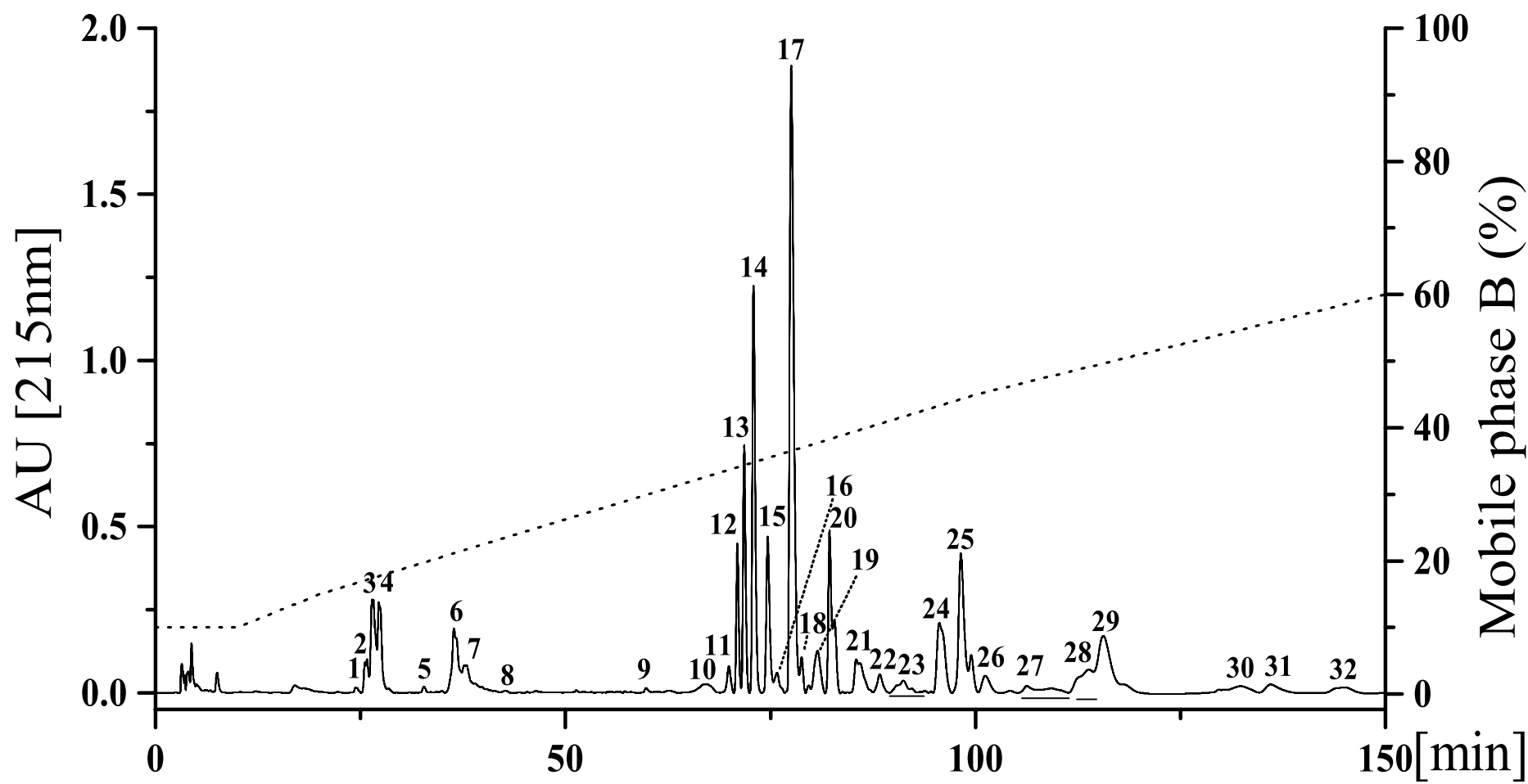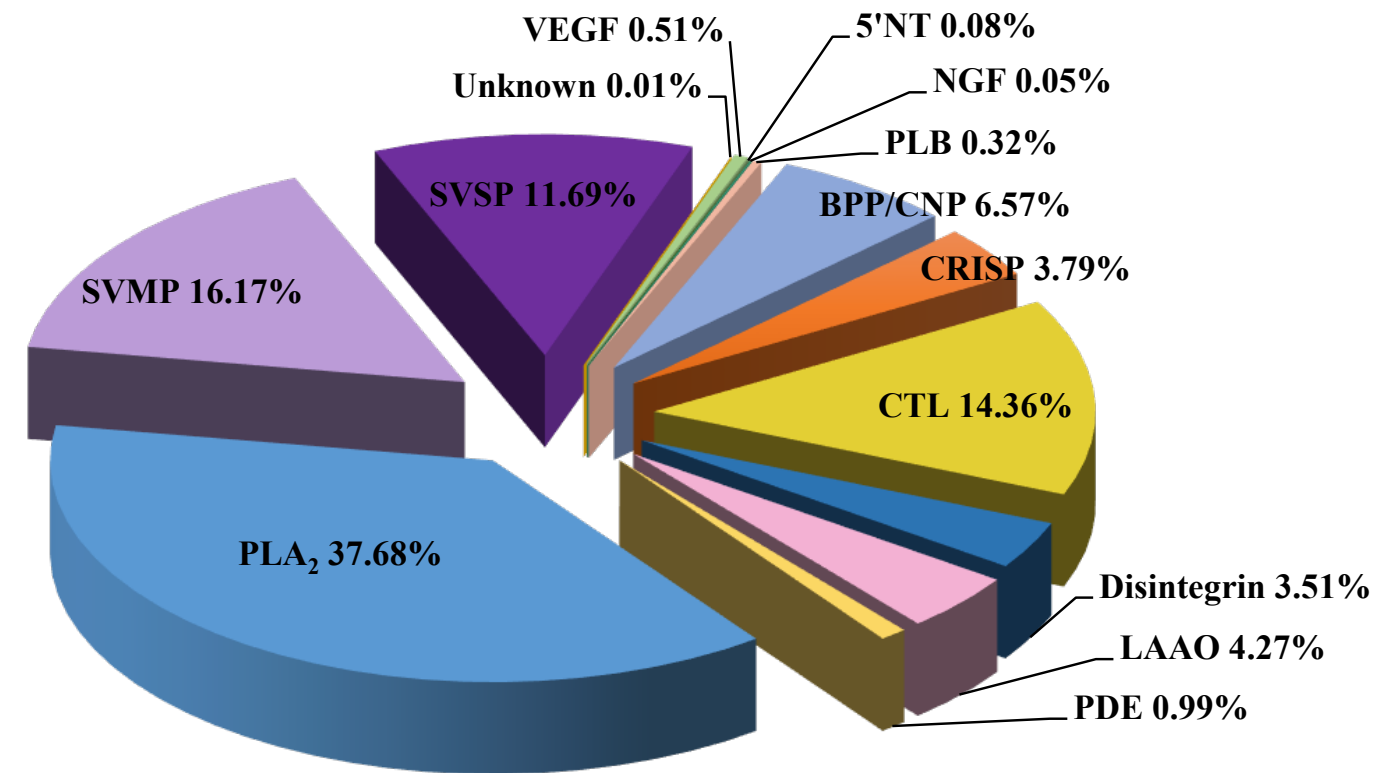

**B**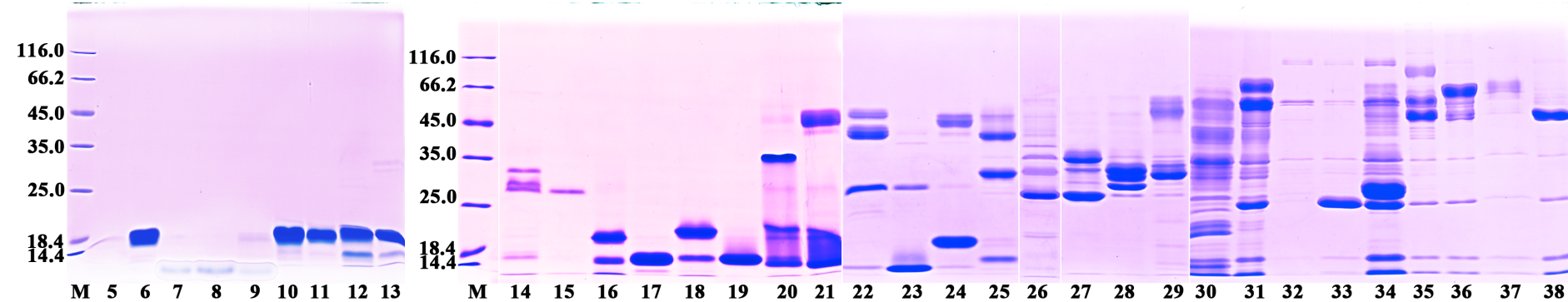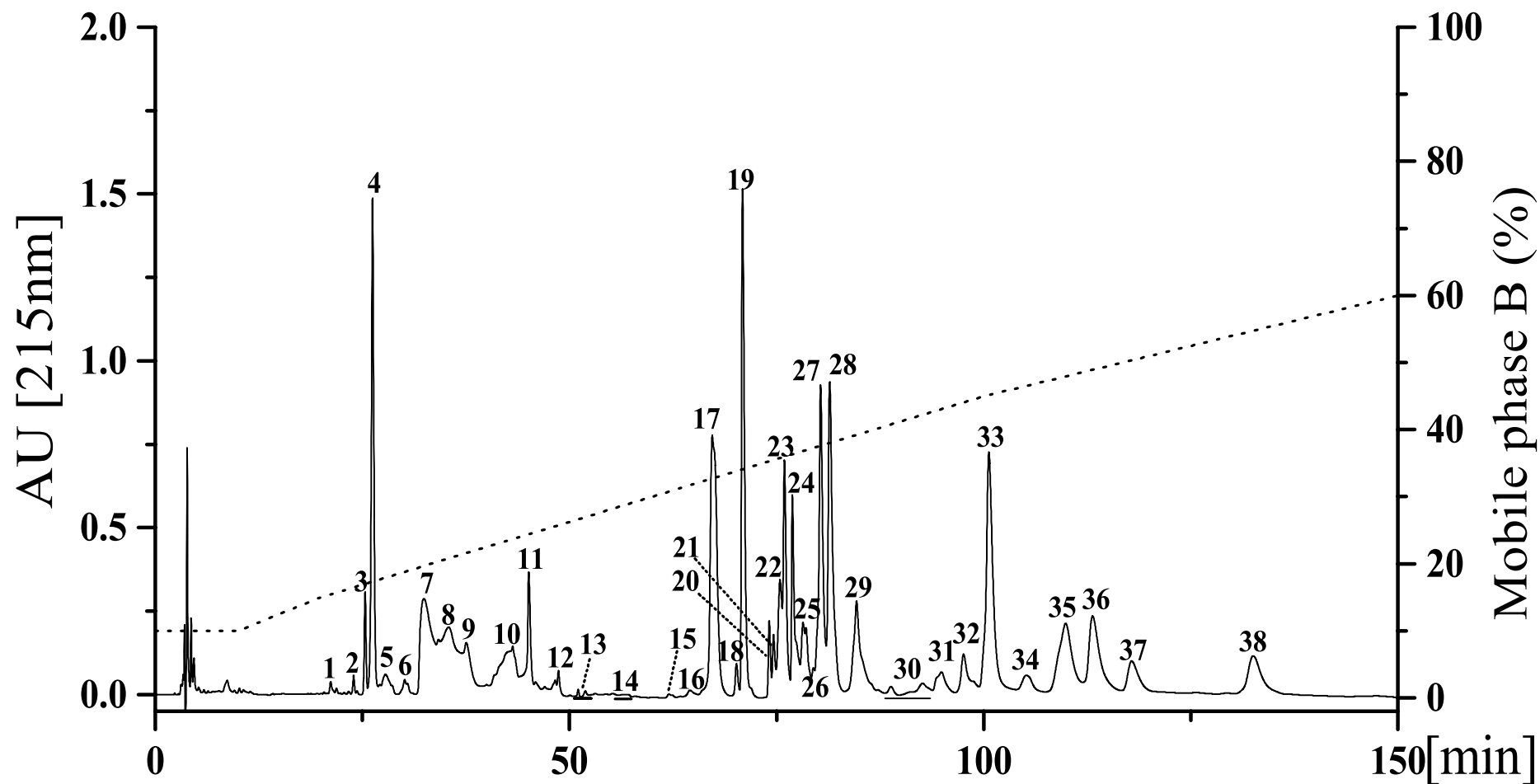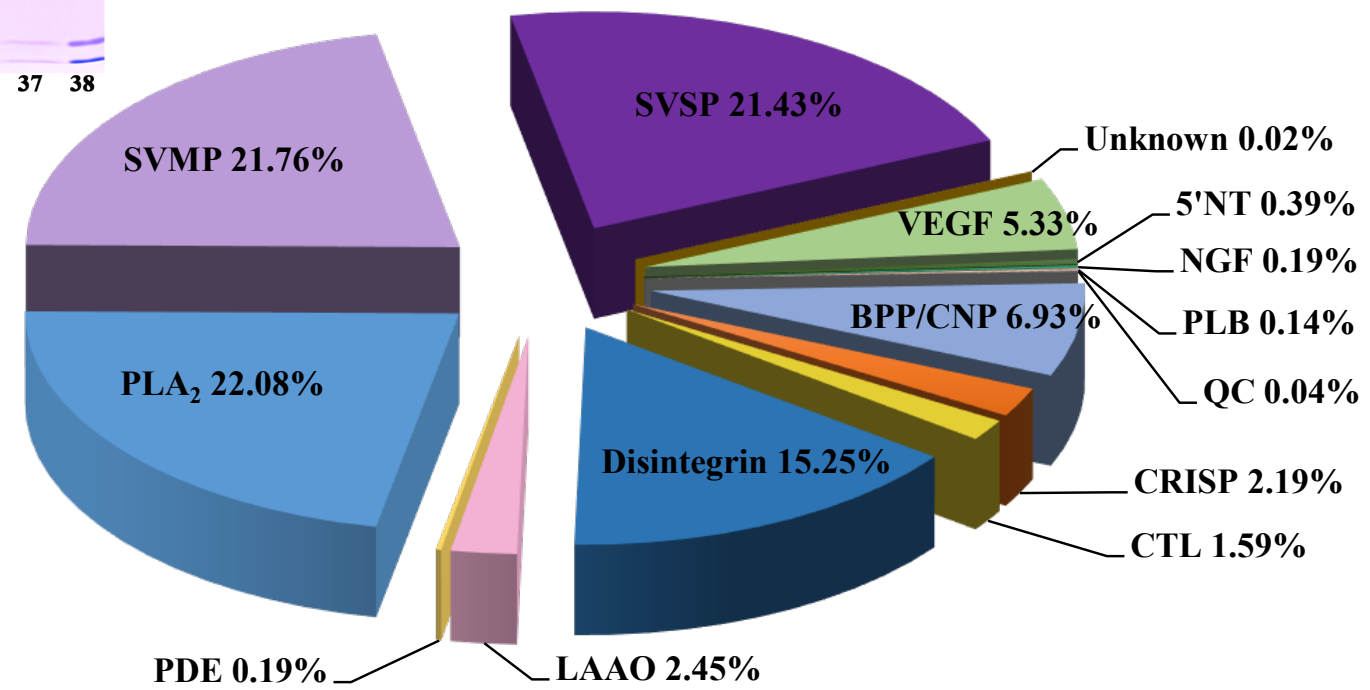

**C**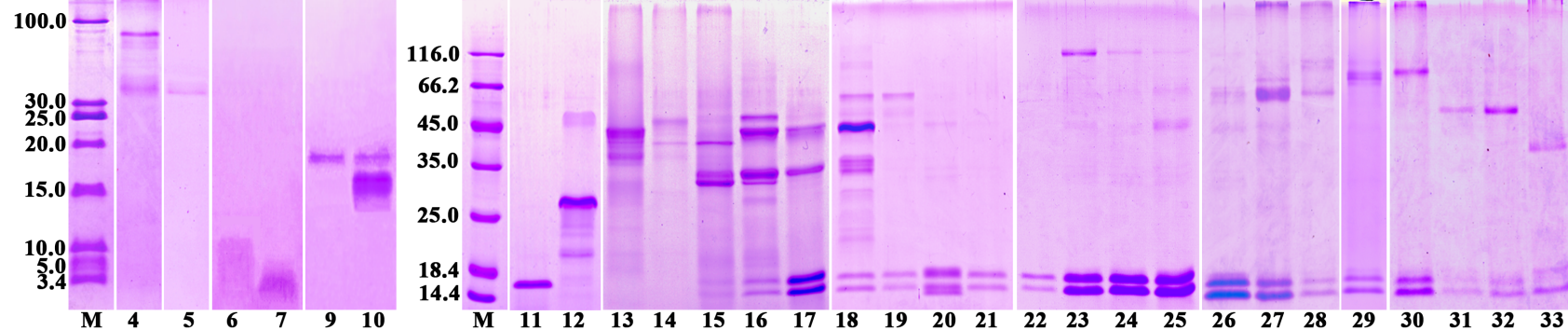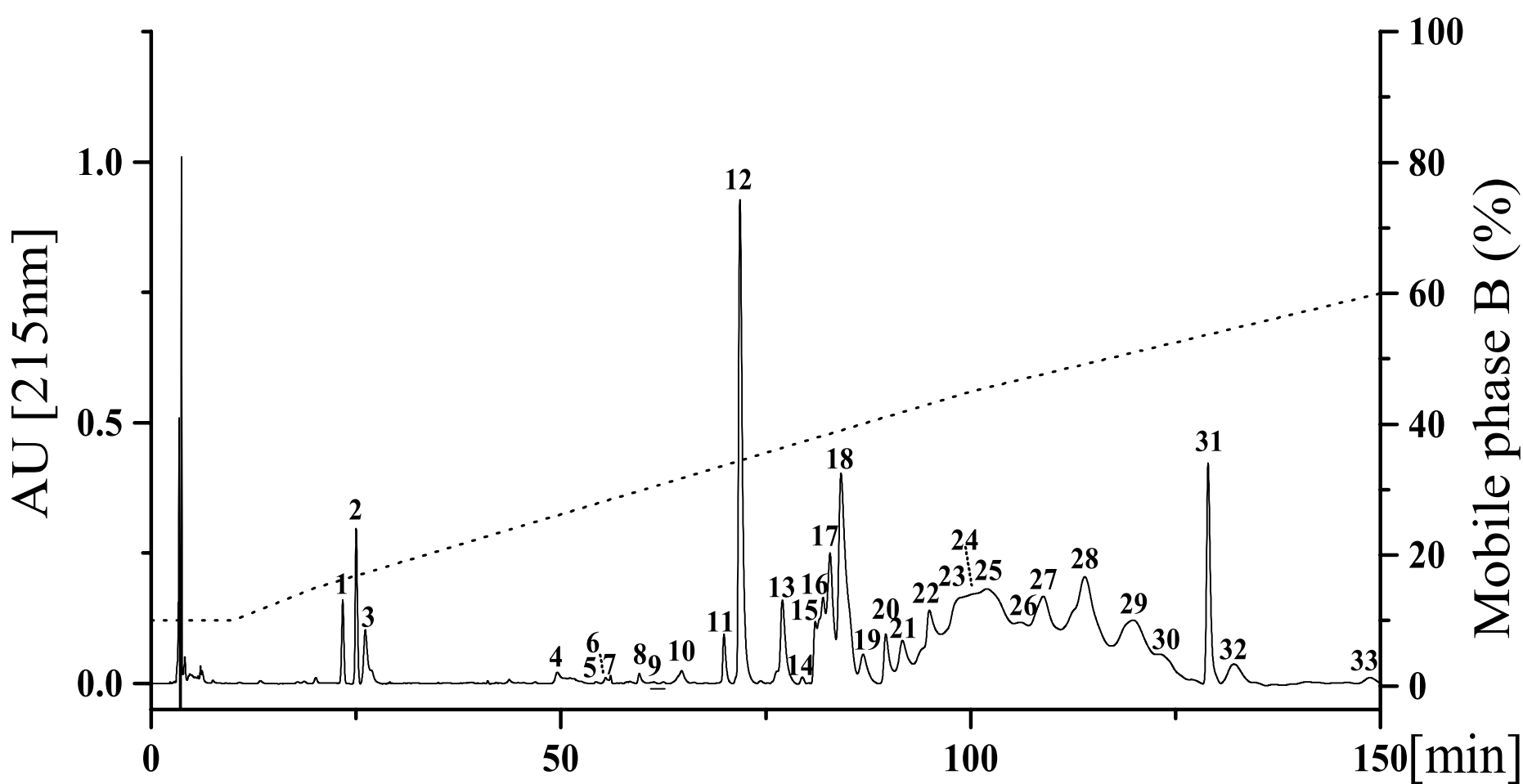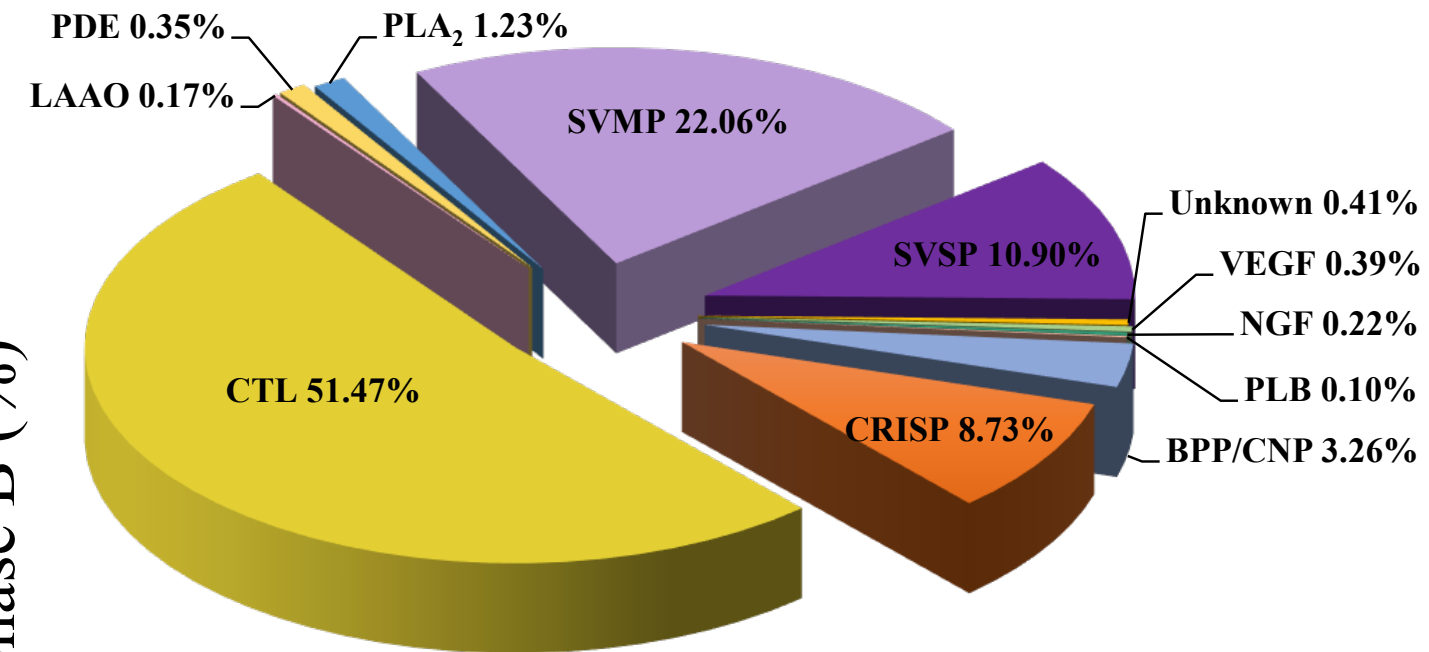

**D**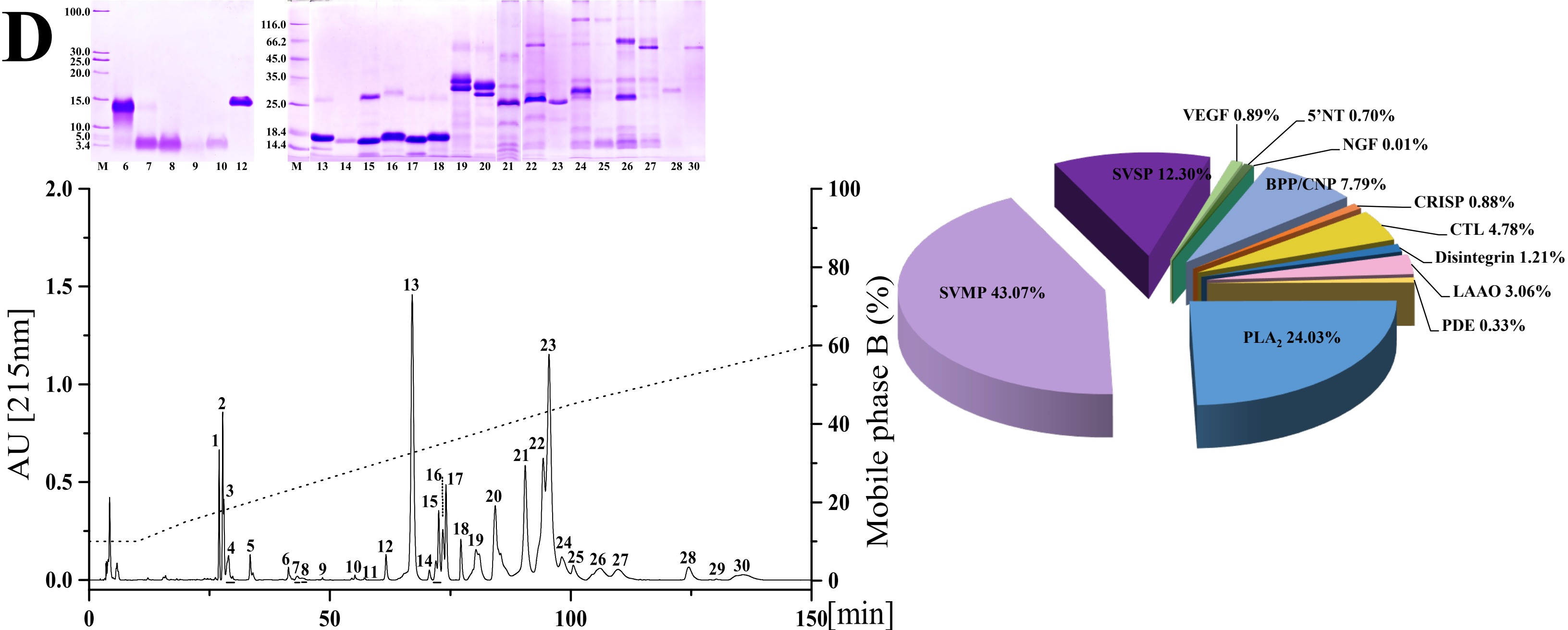

**E**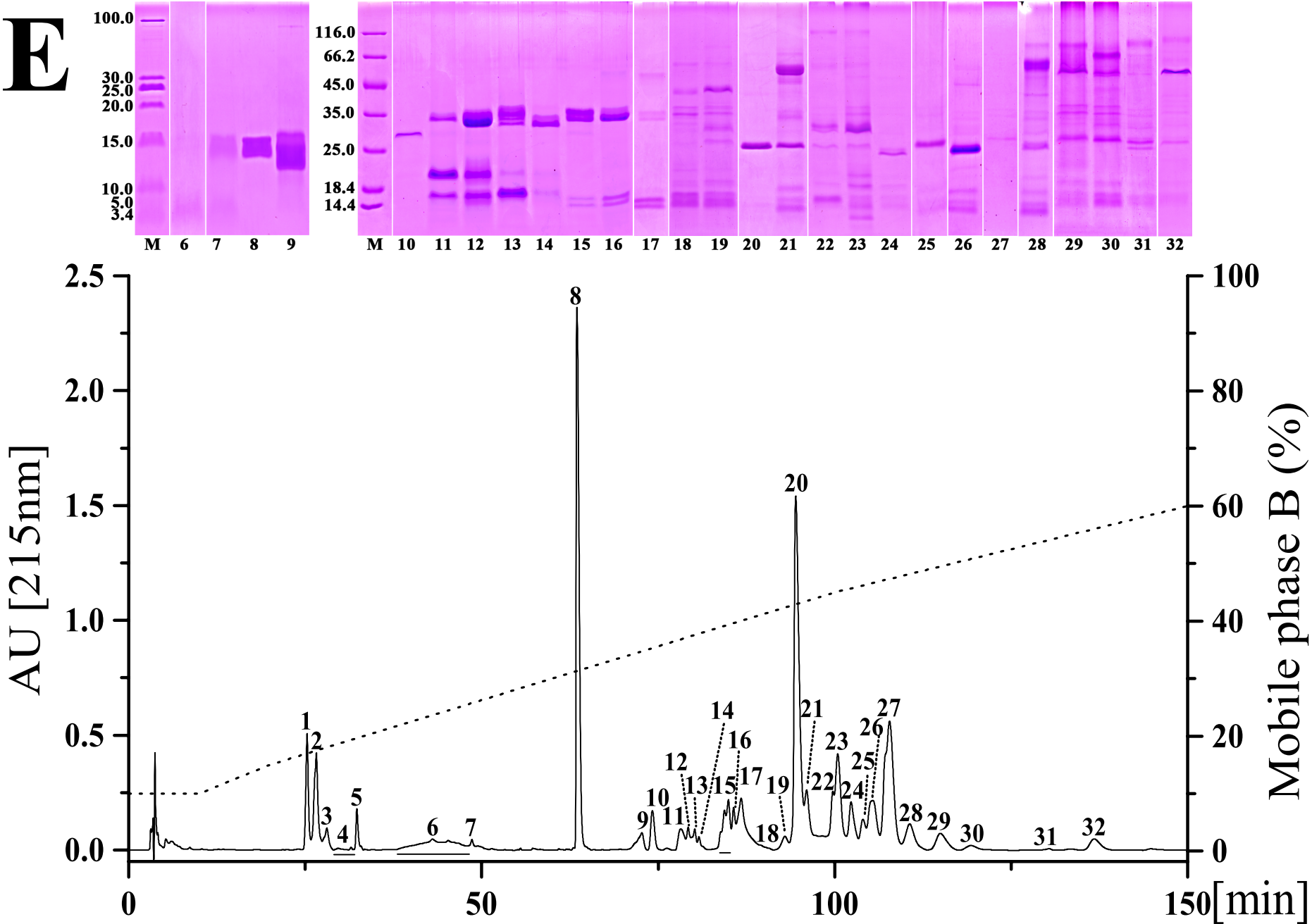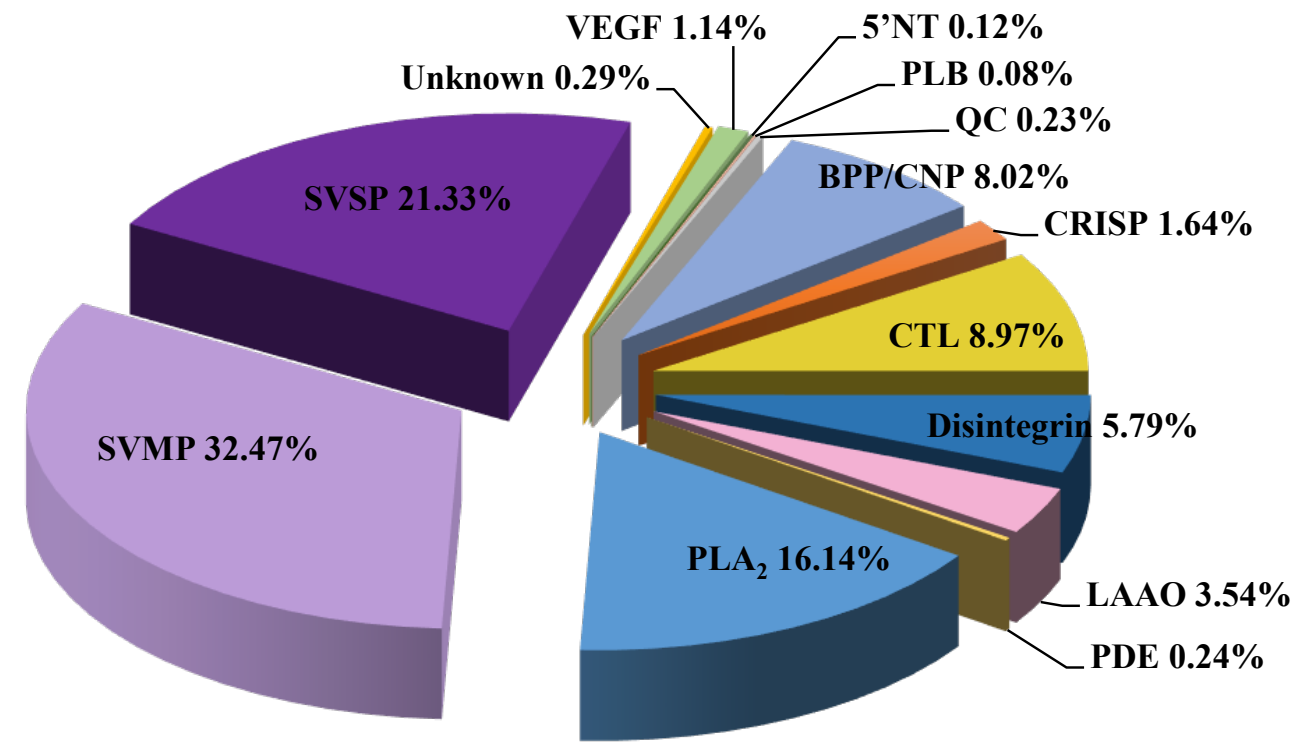

**F**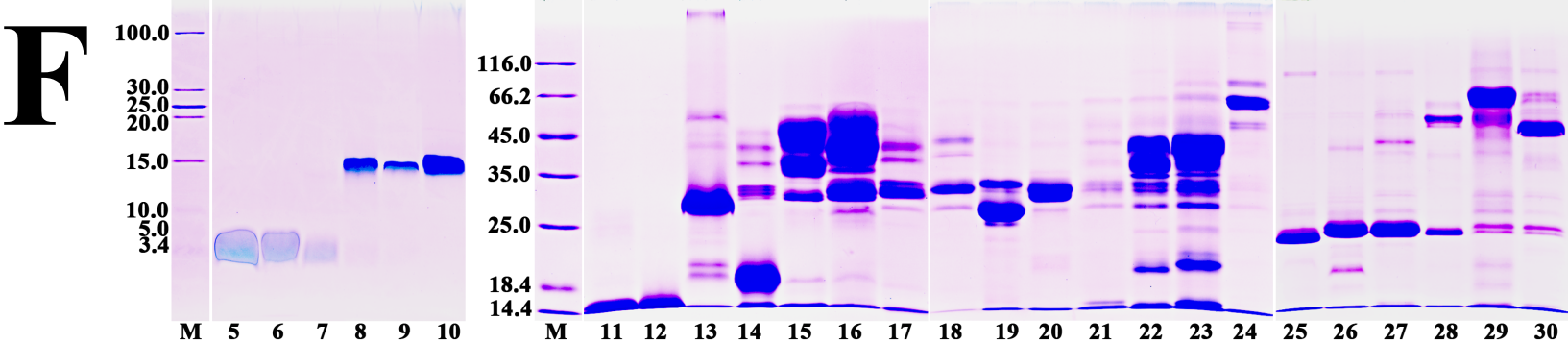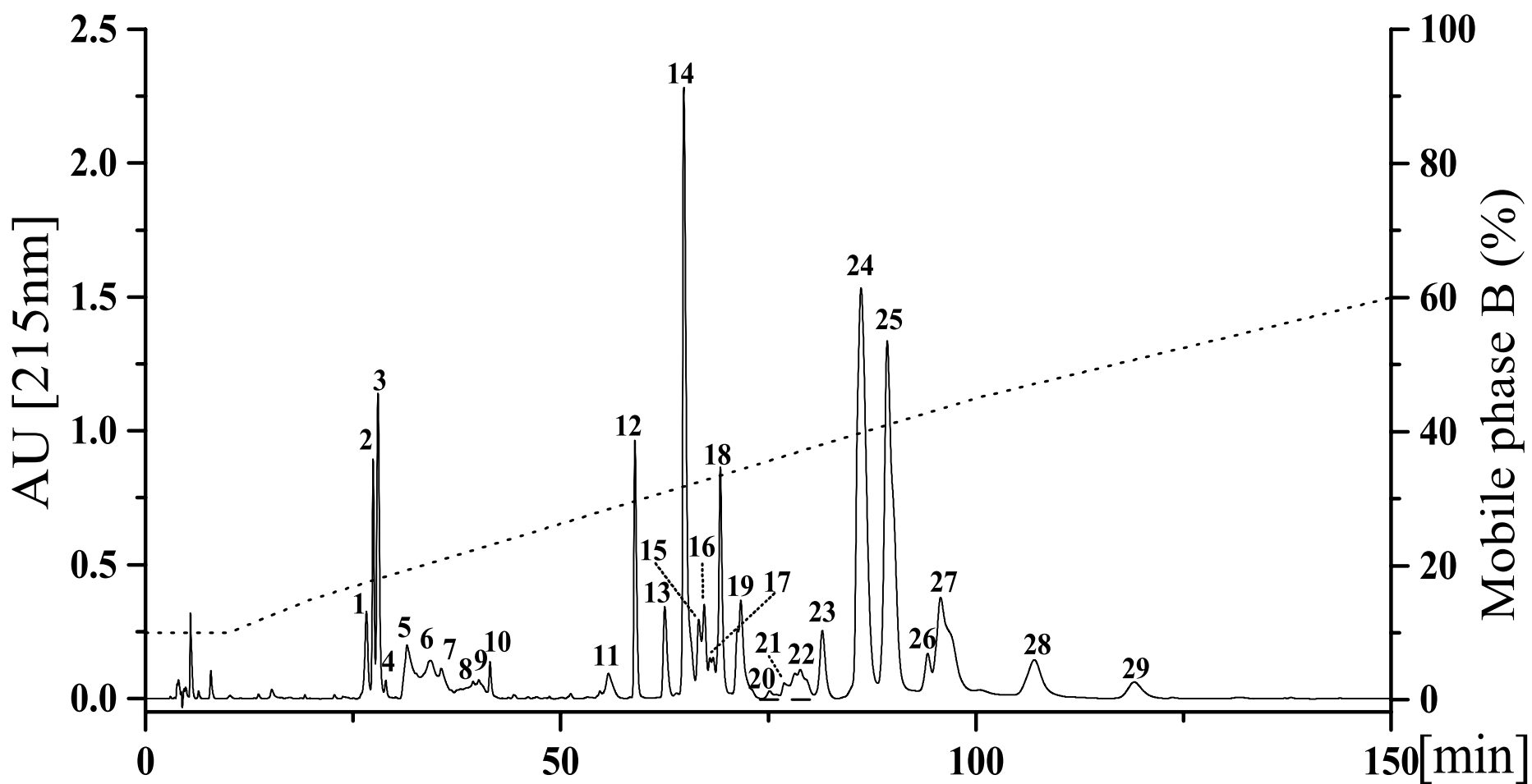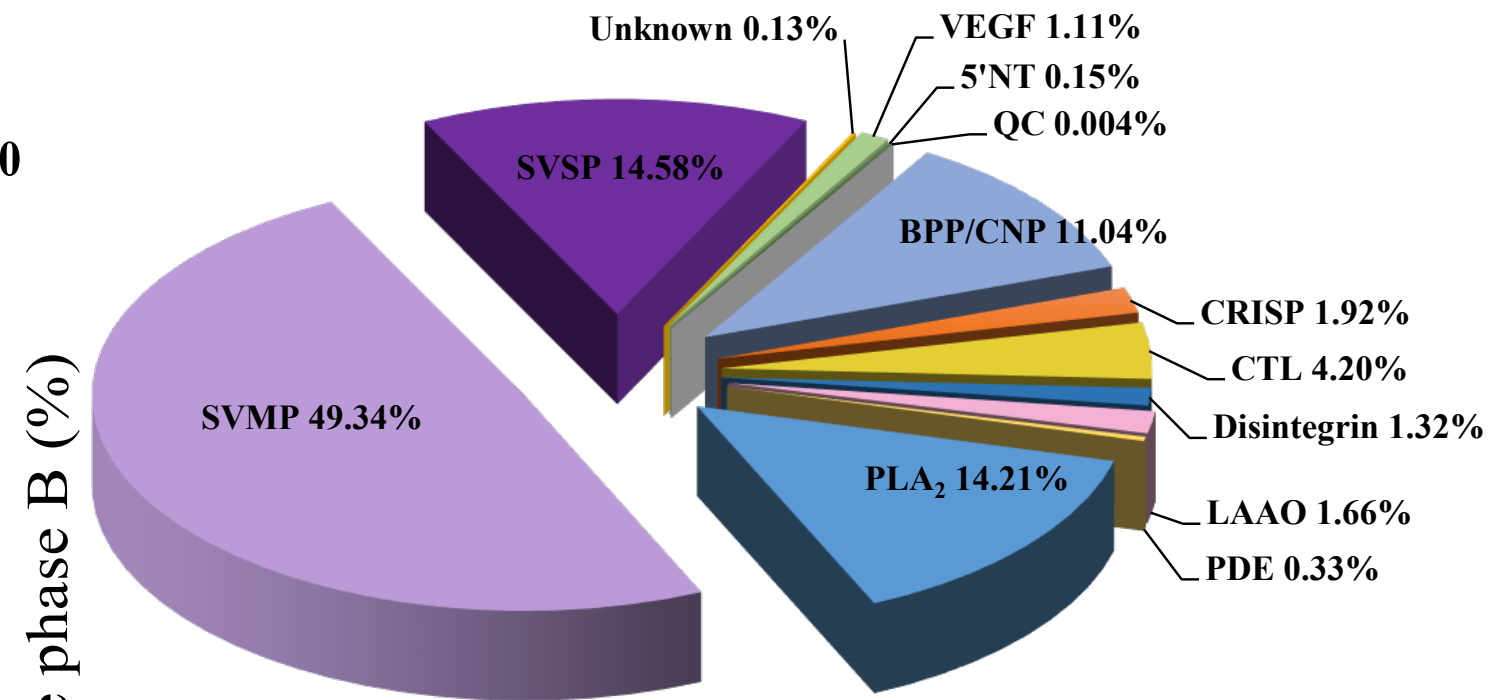

Figure S3

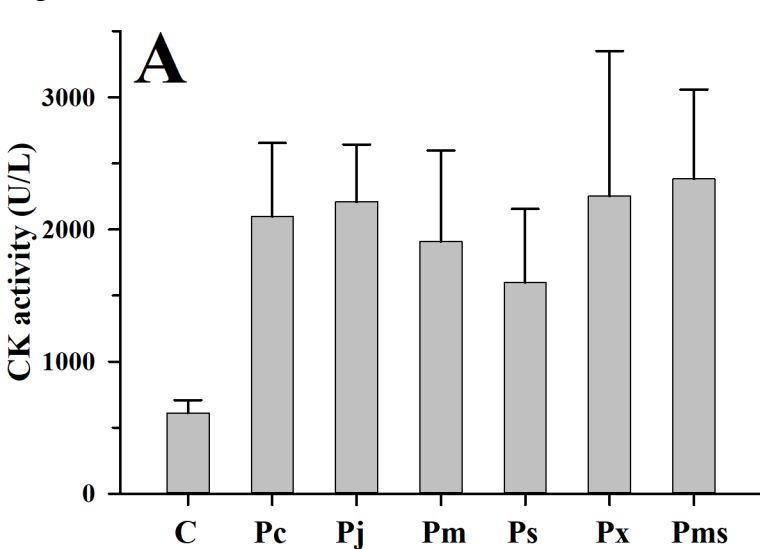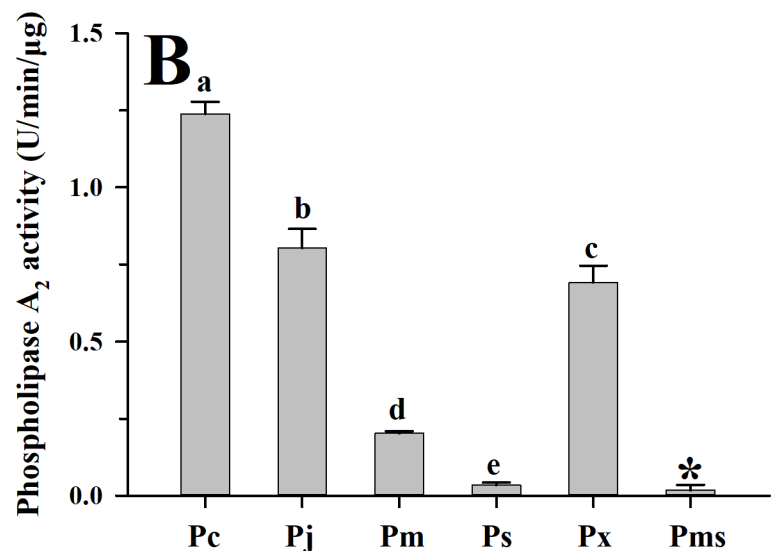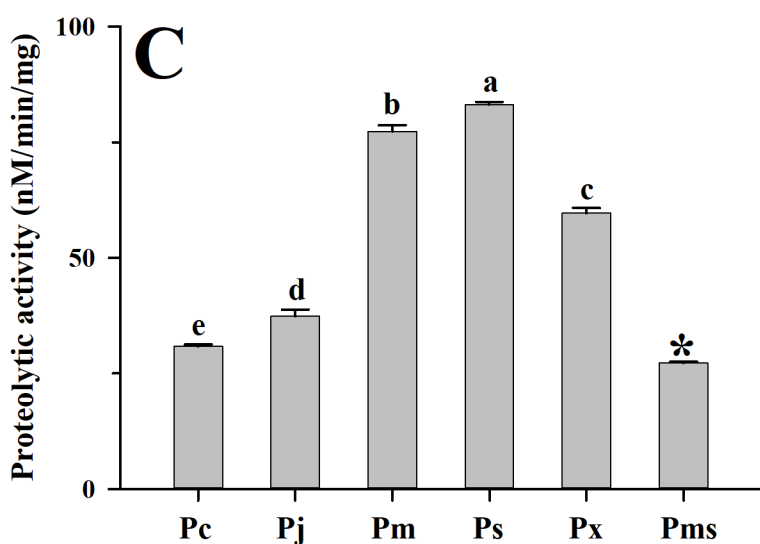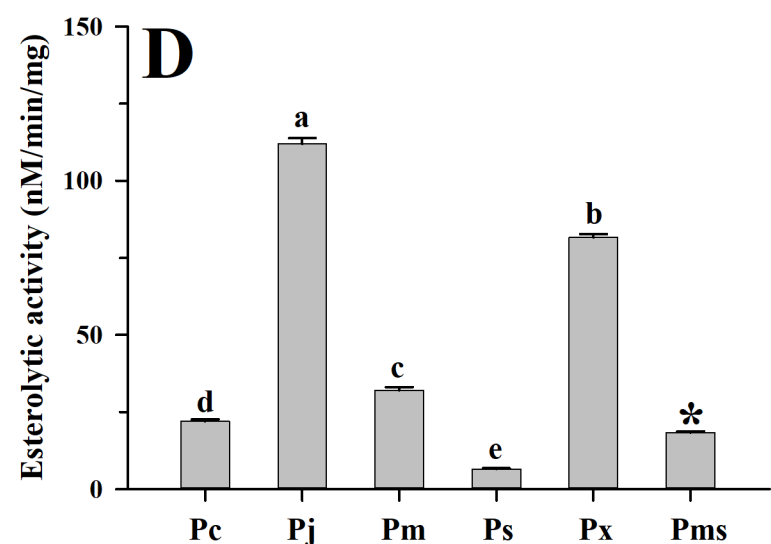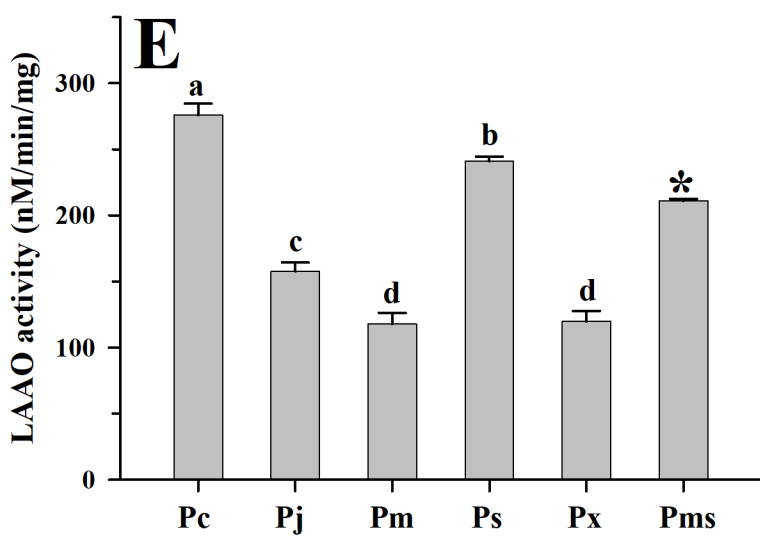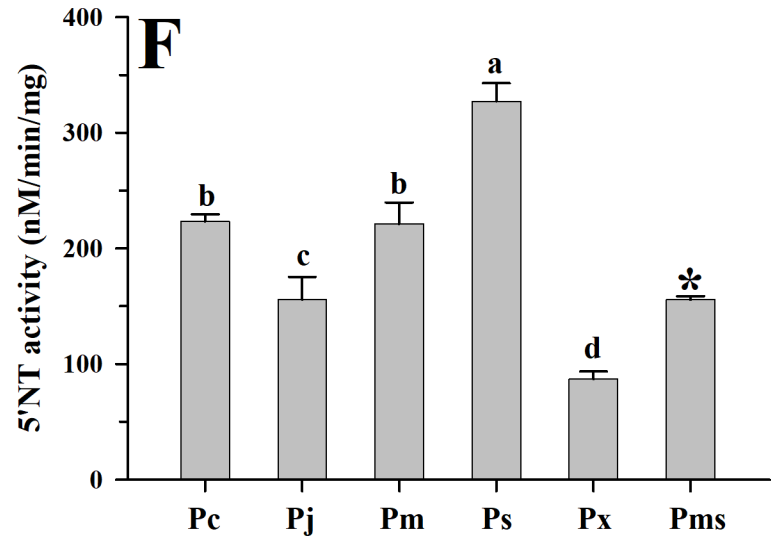

Figure S4

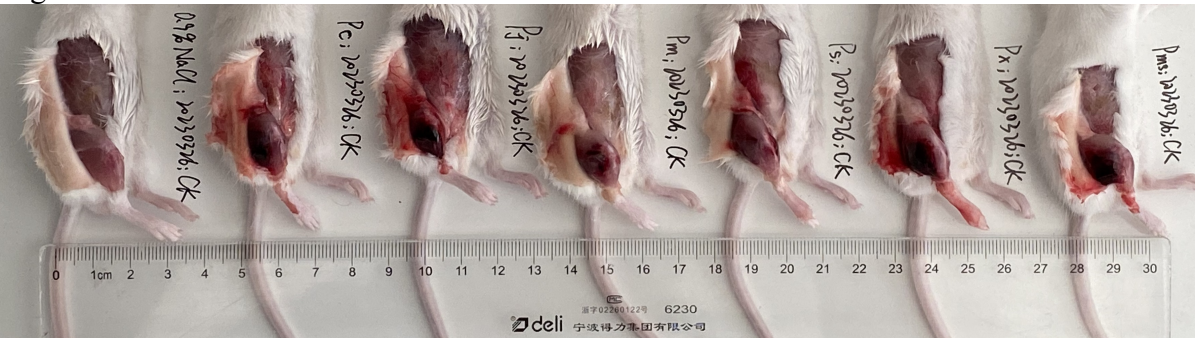

Supplement: Supplementary file 1 [file toxins-15-00350-s001.zip › Supplementary Figures 1-4.pdf]
